# Polyoxypregnane Ester Derivatives and Lignans from *Euphorbia gossypina* var. *coccinea* Pax.

**DOI:** 10.3390/plants11101299

**Published:** 2022-05-13

**Authors:** Reham Hammadi, Norbert Kúsz, Csilla Zsuzsanna Dávid, Peter Waweru Mwangi, Róbert Berkecz, Nikoletta Szemerédi, Gabriella Spengler, Judit Hohmann, Andrea Vasas

**Affiliations:** 1 Department of Pharmacognosy, University of Szeged, Eötvös u. 6, 6720 Szeged, Hungary; reham.hammadi@pharmacognosy.hu (R.H.); kusznorbert@gmail.com (N.K.); davidzsuzsanna88@gmail.com (C.Z.D.); hohmann.judit@szte.hu (J.H.); 2 School of Biological Sciences, University of Nairobi, P.O. Box 30197, Nairobi 00100, Kenya; waweruk2001@gmail.com; 3 Institute of Pharmaceutical Analysis, University of Szeged, 6720 Szeged, Hungary; berkecz.robert@szte.hu; 4 Department of Medical Microbiology, Albert Szent-Györgyi Health Center, Albert Szent-Györgyi Medical School, University of Szeged, 6725 Szeged, Hungary; szemeredi.nikoletta@med.u-szeged.hu (N.S.); spengler.gabriella@med.u-szeged.hu (G.S.); 5 Interdisciplinary Centre of Natural Products, University of Szeged, Eötvös u. 6, 6720 Szeged, Hungary

**Keywords:** Euphorbiaceae, *Euphorbia gossypina* var. *coccinea*, pregnane glycosides, lignans, flavonoids

## Abstract

From the aerial parts of *Euphorbia* *gossypina* var. *coccinea* Pax., eight new pregnane glycosides (euphogossypins A–H, **1**–**8**) of the cynanforidine and deacetylmetaplexigenin aglycons, two new lignans (gossypilignans A and B, **9** and **10**), and four known compounds, namely, the pregnane 12-*O*-benzoyldeaxcylmetaplexigenin (**11**), the lignan 9α-hydroxypinoresinol (**12**), and the flavonoids naringenin (**13**) and quercitrin (**14**) were isolated. The structure elucidation of the new compounds was carried out by a spectroscopic analysis, including HRMS, 1D (^1^H, ^13^C JMOD), and 2D NMR (HSQC, ^1^H–^1^H COSY, HMBC, and NOESY) experiments. The obtained pregnane glycosides were substituted with acetyl and benzoyl ester moieties, and sugar chains containing thevetose, cymarose, digitoxose, and glucose monosaccharides. All of the compounds are described for the first time from *E. gossypina* var. *coccinea*. The isolated pregnanes and lignans were tested for their antiproliferative activity on HeLa cells using the MTT assay; the compounds exerted no significant effect against the tumor cells.

## 1. Introduction

Plants belonging to the genus *Euphorbia* are known to possess considerable chemical, medicinal, and economic importance [1]. Terpenes, including diterpenes and triterpenes, steroids, cerebrosides, glycerols, and phenolic compounds are characteristic constituents of *Euphorbia* species [2]. In the course of our work focusing on the isolation of special metabolites from various *Euphorbia* species, the chemical composition of *Euphorbia gossypina* var. *coccinea* Pax. (Euphorbiaceae) was thoroughly investigated [3]. The aim of our work was the identification of the special metabolites of the plant. This plant is a perennial, much-branched, succulent, herbaceous, evergreen shrub native to Kenya and Tanzania. Preparations of *E. gossypina* have long been used in traditional medicine for the treatment of swollen legs and general body pain. Moreover, it is applied as eye drops in the treatment of conjunctivitis and warts [4]. The diluted latex of small twigs is taken to treat laryngitis [5]. In Somalia, the latex is applied to treat mange in livestock [6]. There is no literature data on the phytochemistry and pharmacology of *E. gossypina* var. *coccinea*.

Herein, we report the isolation and structure elucidation of fourteen compounds, among them being eight new polyoxypregnane ester derivatives (euphogossypins A–H, **1**–**8**), two new lignans (gossypilignans A and B, **9** and **10**), and four known compounds, including the pregnane 12-*O*-benzoyldeacylmetaplexigenin (**11**), the lignan 9α-hydroxypinoresinol (**12**), and the flavonoids naringenin (**13**) and querctirin (**14**) from *E. gossypina* var. *coccinea*.

Pregnane glycosides are C_21_ steroidal natural compounds, in which the pregnane part is attached to different sugars. These compounds demonstrate a fair degree of diversity in their aglycone part with different numbers and types of sugar units being attached to the aglycone at position C-3 [7]. The sugar part of these compounds can be a mono- or disaccharide, or an oligosaccharide chain arranged mainly linearly to the pregnane skeleton through an acetal linkage. The characteristic monosaccharides of pregnane glycosides are d-glucose, l-rhamnose, d-cymarose, d-oleandrose, d-allose, and d-digitoxose. The occurrence of pregnane glyosides is characteristic of the Asclepiadaceae, Apocynaceae, Malpighiaceae, Ranunculaceae, and Zygophyllaceae families [8]. Pregnane glycosides are reported to possess noteworthy pharmacological properties, such as immunosuppressant, cytotoxic, antidepressant, anti-inflammatory, antioxidant, antibacterial, antifungal, and antiproliferative activities [8]. Therefore, the isolated compounds were tested for their antiproliferative activity against the HeLa cell line using the MTT assay.

## 2. Results and Discussion

### 2.1. Isolation of Compounds

**Figure 1:**
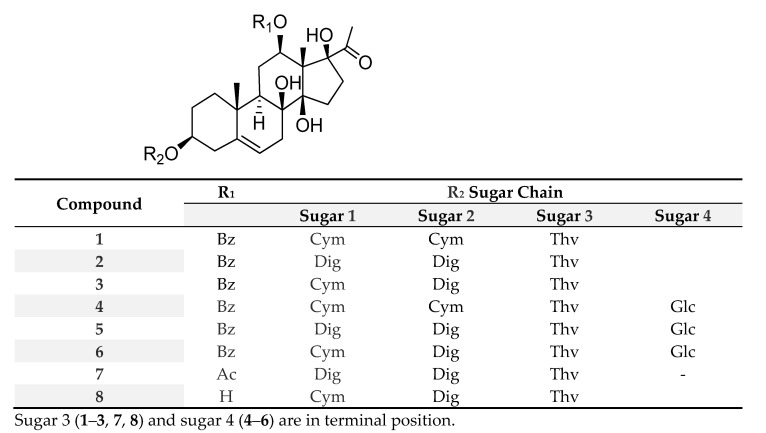

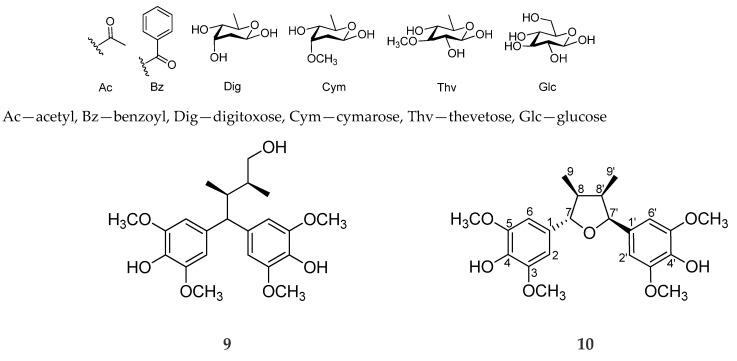
New isolated compounds (**1**–**10**) from *E. gossypina* var. *coccinea*.

The dried and ground aerial parts of the plant were extracted with methanol. After evaporation of the methanol, the extract was dissolved in 50% aqueous methanol, and a solvent–solvent partition was performed with *n*-hexane, CHCl_3_, and EtOAc. The CHCl_3_ extract was separated by normal (NP) and reversed-phase (RP) vacuum liquid chromatography (VLC), and then it was purified by preparative thin layer chromatography (prep. TLC) and high-performance liquid chromatography (HPLC) to yield eight new pregnane glycosides (**1**–**8**), two new lignans (**9**, **10**), one known pregnane and one lignan, and two known flavonoids (Figure 1).

### 2.2. Structure Elucidation of the Compounds

The structure determination of the isolated compounds was carried out by an extensive spectroscopic analysis, including one- and two-dimensional NMR and HRMS measurements.

**Figure 2:**
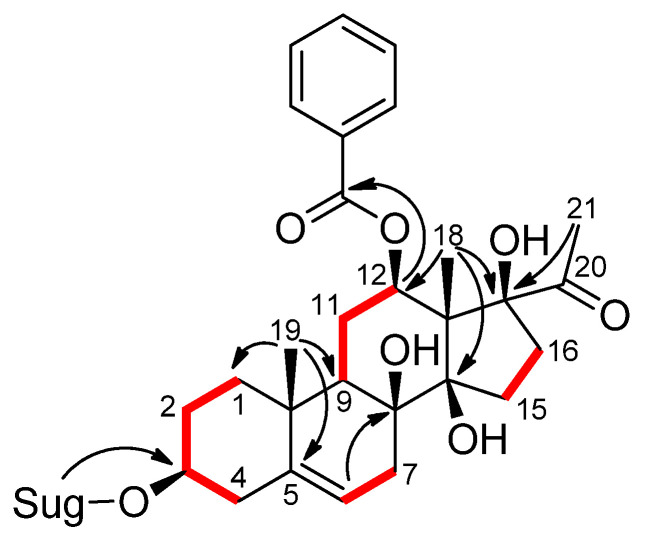
The ^1^H-^1^H COSY (**—**) and key HMBC (→) correlations of **1**.

Compound **1** was obtained as a white amorphous powder. Its molecular formula was determined as C_49_H_72_O_17_ by the HRESIMS ion at *m/z* 955.4660 [M + Na]^+^ (calcd for C_49_H_72_O_17_Na, 955.4662). The ^1^H NMR spectrum of **1** showed the resonances of three anomeric protons at δ_H_ 4.85 (dd, *J* = 2.0 and 9.0 Hz), 4.76 (dd, *J* = 1.8 and 9.5 Hz), and 4.30 (d, *J* = 7.8 Hz), three methoxy groups at δ_H_ 3.42, 3.44, and 3.65 (each 3H, s), and three secondary methyl groups at δ_H_ 1.22 (d, *J* = 6.3 Hz), 1.27 (d, *J* = 6.2 Hz), and 1.31 (d, *J* = 6.2 Hz), suggesting the presence of a trisaccharide unit in **1** composed of deoxymethyl sugars. Moreover, the ^1^H NMR spectrum contained signals ascribable to three methyl groups displayed at δ_H_ 1.12 (3H, s), 1.54 (3H, s), and 2.06 (3H, s), and to an olefinic proton at δ_H_ 5.38 (1H, br s). The proton signals at δ_H_ 7.93 (2H, dd), 7.43 (2H, t), and 7.55 (1H, t) showed the presence of a benzoyl group in the molecule. The JMOD spectrum indicated that compound **1** contained two carbonyls, seven non-protonated carbons (of which three were oxygenated), twenty-two methines (of which eleven were oxygenated), nine methylenes, and nine methyl carbons (of which three were methoxy). The ^1^H and ^13^C JMOD NMR data dictated that **1** was a pregnane glycoside. Of these, 21 carbons were assigned to a pregnane skeleton, 7 to a benzoyl function, and 19 to a trisaccharide moiety. The HMBC correlations between H-19 (δ_H_ 1.12) and C-1 (δ_C_ 38.9), C-5 (δ_C_ 140.8), C-9 (δ_C_ 43.8), and C-10 (δ_C_ 37.3) suggested the position of a double bond at C-5/C-6. The HMBC correlations between H-18 (δ_H_ 1.54) and C-12 (δ_C_ 73.3), C-14 (δ_C_ 88.1), and C-17 (δ_C_ 91.6), between H-21 (δ_H_ 2.06) and C-17 (δ_C_ 91.6), and H-6 (δ_H_ 5.38) and C-8 (δ_C_ 74.4) demonstrated the positions of hydroxy groups at C-8, C-14, and C-17 (Figure 2). The aglycone moiety of **1** was, therefore, determined to be 12*β*-benzoyloxy-3*β*,8*β*,14*β*,17*β*-tetrahydroxypregn-5-ene (cynanforidine), a C/D-*cis*-polyoxypregnane ester [9]. The relative configuration of the molecule was determined by the analysis of NOESY correlations and literature data of similar structures reported previously.

The aglycone of **1** was supposed to have the same configuration as those of pregnanes isolated from *Gymnema sylvestre* [10]. The multiplicity of H-12 (δ_H_ 4.83 (dd, *J* = 4.2, 11.9 Hz)) implied that the configuration of H-12 was axial (α-configuration, Figure 2). The NOESY correlations between H-3 (δ_H_ 3.57), H_α_-1 (δ_H_ 1.89), and H_α_-4 (δ_H_ 2.40), and between H-12 (δ_H_ 4.83) and H-9 (δ_H_ 1.59) determined the configurations of the oxygenated groups at C-3 and C-12 to be β. NOESY correlations detected between H*β*-1 (δ_H_ 1.13) and H*β*-4 (δ_H_ 2.29) showed the α-position of these protons. The C-12 benzoyl group was confirmed by HMBC correlation from H-12 (δ_H_ 4.83) to the benzoyl carbonyl C-1′ (δ_C_ 165.5). The large coupling constants between the H-1 and H-2 of monosaccharide moieties, and the HMBC correlations between Thv H-1 (δ_H_ 4.30) and Cym II C-4 (δ_C_ 82.8), Cym II H-1 (δ_H_ 4.76) and Cym I C-4 (δ_C_ 82.7), and between Cym I H-1 (δ_H_ 4.85) and aglycone C-3 (δ_C_ 78.0) indicated the sugar linkages as *β*-d-thevetopyranosyl-(1→4)-*β*-d-cymaropyranosyl-(1→4)-*β*-d-cymaropyranoside and at C-3 of aglycone. Based on the above evidence, the structure of **1** was elucidated as cynanforidine 3-*O*-*β*-d-thevetopyranosyl-(1→4)-*β*-d-cymaropyranosyl-(1→4)-*β*-d-cymaropyranoside, a new compound named euphogossypin A.

Compound **2** was isolated as a white amorphous powder. Its ^1^H and JMOD spectra exhibited the characteristic signals of a pregnane aglycone, one benzoyl unit, and three sugar moieties (Table 1). In addition, NMR data of **2** were similar to those of euphogossypin A (**1**), except for the difference in sugar units at C-3. A careful analysis of NMR data led to the conclusion that two cymarose units were replaced by two digitoxose monosaccharides. The HMBC correlations between Thv H-1 (δ_H_ 4.82) and Dig II C-4 (δ_C_ 84.1), Dig II H- 1 (δ_H_ 5.41) and Dig I C-4 (δ_C_ 83.9), and between Dig I H-1 (δ_H_ 5.48) and aglycone C-3 (δ_C_ 78.1) confirmed the sugar linkages to be 3-*O*-*β*-d-thevetopyranosyl-(1→4)-*β*-d-digitoxopyranosyl-(1→4)-*β*-d-digitoxopyranoside. Consequently, compound **2** was identified as a new compound, cynanforidine 3-*O*-*β*-d-thevetopyranosyl-(1→4)-*β*-d-digitoxopyranosyl-(1→4)-*β*-d-digitoxopyranoside, named euphogossypin B.

The molecular formula of **3** was determined as C_48_H_70_O_17_ by the HRESIMS molecular ion at *m/z* 941.4508 [M + Na]^+^ (calcd for C_48_H_70_O_17_Na 941.4510). The ^1^H and JMOD data (Table 1) suggested that the structure of **3** was similar to those of **1** and **2**, except for the difference in monosaccharide units in the C-3 sugar chain. The sugar moieties were found to be d-cymarose, d-digitoxose, and d-thevetose. The sugar linkages (*β*-d-thevetopyranosyl-(1→4)-*β*-d-digitoxopyranosyl-(1→4)-*β*-d-cymaropyranoside) to each other and to the pregnane skeleton were confirmed by the HMBC correlations from Thv H-1 (δ_H_ 4.35) to Dig C-4 (δ_C_ 83.8), from Dig H-1 (δ_H_ 4.89) to Cym C-4 (δ_C_ 83.9), and from Cym H-4 (δ_H_ 3.24) to C-3 (δ_C_ 79.3). Similarly to those of **1** and **2**, the aglycone was found to be 12*β*-benzoyloxy-3*β*,8*β*,14*β*,17*β*-tetrahydroxypregn-5-ene (cynanforidine). Consequently, the structure of **3** was determined as cynanforidine 3-*O-β*-d-thevetopyranosyl-(1→4)-*β*-d-digitoxopyranosyl-(1→4)-*β*-d-cymaropyranoside and named euphogossypin C.

HRESIMS and NMR data of compounds **4**–**6** suggested that they were tetrasaccharide derivatives due to the presence of four anomeric carbon and proton signals, one more than observed for compounds **1**–**3**. The additional sugar unit was identified as d-glucopyranose (Table 2). The polyoxypregnane ester aglycone was the same as in compounds **1**–**3**. The large coupling constant between H-1 and H-2 of the glucose unit, and the HMBC correlations between Glc H-1 (δ_H_ 4.43) and Thv C-4 (δ_C_ 82.8) in **4**–**6**, indicated the sugar linkages as *β*-d-glucopyranosyl-(1→4)-*β*-d-thevetopyranosyl-(1→4)-*β*-d-cymaropyranosyl-(1→4)-*β*-d-cymaropyranoside in **4**, *β*-d-glucopyranosyl-(1→4)-*β*-d-thevetopyranosyl-(1→4)-*β*-d-digitoxopyranosyl-(1→4)-*β*-d-digitoxopyranoside in **5**, and *β*-d-glucopyranosyl-(1→4)-*β*-d-thevetopyranosyl-(1→4)-*β*-d-digitoxopyranosyl-(1→4)-*β*-d-cymaropyranoside in **6**. Based on the above evidence, the structure of **4** was deduced to be cynanforidine *β*-d-glucopyranosyl-(1→4)-*β*-d-thevetopyranosyl-(1→4)-*β*-d-cymaropyranosyl-(1→4)-*β*-d-cymaropyranoside, and was named euphogossypin D, **5** was determined as cynanforidine *β*-d-glucopyranosyl-(1→4)-*β*-d-thevetopyranosyl-(1→4)-*β*-d-digitoxopyranosyl-(1→4)-*β*-d-digitoxopyranoside and named euphogossypin E, and **6** was characterized as cynanforidine *β*-d-glucopyranosyl-(1→4)-*β*-d-thevetopyranosyl-(1→4)-*β*-d-digitoxopyranosyl-(1→4)-*β*-d-cymaropyranoside and named euphogossypin F.

The NMR data of compound **7** were very similar to that of **2**, with the only difference between them being the replacement of the benzoyl moiety into an acetyl substituent at C-12. Therefore, the aglycone of **7** was identified as metaplexigenin (Table 3) [11]. The connecting sugar chain was determined as *β*-d-thevetopyranosyl-(1→4)-*β*-d-digitoxopyranosyl-(1→4)-*β*-d-digitoxopyranoside based on the 1D and 2D NMR spectral data. Compound **7** was, thus, characterized as metaplexigenin *β*-d-thevetopyranosyl-(1→4)-*β*-d-digitoxopyranosyl-(1→4)-*β*-d-digitoxopyranoside and named euphogossypin G.

Compound **8** wore the same C-3 trisaccharide sugar chain, *β*-d-thevetopyranosyl-(1→4)-*β*-d-digitoxopyranosyl-(1→4)-*β*-d-cymaropyranoside, as **3** (Table 3). By the NMR data, it was determined to be a deacetylmetaplexigenin derivative and named euphogossypin H.

**Figure 3:**
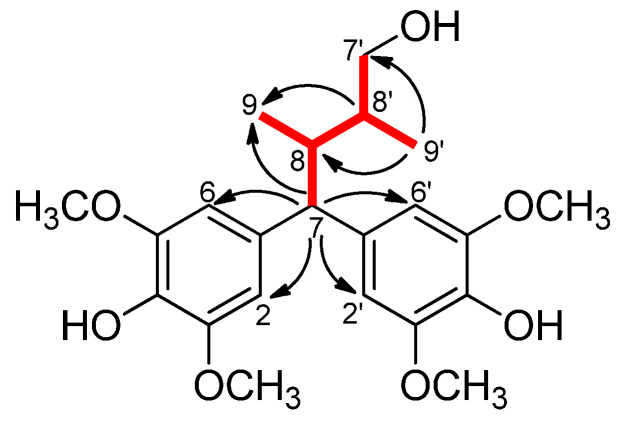
Selected ^1^H-^1^H COSY (**—**) and HMBC (→) correlations of **9**.

Gossypilignan A (**9**) was obtained as a pale yellow, amorphous solid. The molecular formula of **9** was determined as C_22_H_30_O_7_ from its HRESIMS ion observed at *m/z* 429.1889 [M + Na]^+^ (calcd for C_22_H_30_O_7_Na, 429.1884). The ^1^H and ^13^C JMOD NMR data indicated the presence of four methoxy (δ_H_ 2 × 3.82, s, and 2 × 3.83, s; δ_C_ 4 × 56.8), two methyl (δ_H_ 0.68, d, *J* = 6.9 Hz, and 0.76, d, *J* = 7.0 Hz; δ_C_ 10.0 and 12.1), one methylene (δ_H_ 3.35 and 3.45; δ_C_ 67.3), and three methine groups (δ_H_ 1.77, m, 2.62, m, 3.52, d; δ_C_ 37.0, 37.2, and 57.9) (Table 4). Additionally, the ^1^H NMR spectrum of **9** showed aromatic protons at δ_H_ 6.64, s and 6.66, s (2H each), implying that the aromatic rings were tetrasubstituted. The ^13^C NMR spectrum of **9** also supported the presence of six oxygenated aromatic carbons at δ_C_ 149.1, 149.2, and 134.7 (each 2C). According to the HMBC correlations from H-2, H-6, H-2′, and H-6′ to C-7, both aromatic rings were attached to C-7. The ^1^H-^1^H COSY correlations of H-7/H-8/H-8′/H-7′, H-8/H-9, and H-8′/H-9′, as well as the HMBC correlations from H-7 to C-8, C-8′, and C-9, indicated the presence of a 2,3-dimethylbutane moiety (Figure 3). By comparison, the skeleton of **9** was found to be the same as that of kadangustin J [12]. According to the chemical shift of δ_C_ 67.3 (C-7′), a hydroxy group should be placed at C-7′. Four methoxy groups were located at C-3, C-5, C-3′, and C-5, respectively, while C-4 and C-4′ were substituted with hydroxy groups. Thus, the structure of compound **9** was determined to be 4,4-di-(4-hydroxy-3,5-dimethoxyphenyl)-2,3-dimethylbutanol and named gossypilignan A. As compound **9** was a 7,7-diaryl-8,8′-dimethylbutan-1-ol lignan, it had two chiral centers. Based on the investigation of Davidson et al., *syn*- and *anti*-isomers of such compounds can be distinguished based on the significant differences between their ^1^H NMR data [13]; therefore, as in the case of **9**, both methyl groups were *β*-oriented, proving that this compound was a *syn*-isomer.

The molecular formula of **10** was determined as C_22_H_28_O_7_ by the HRESIMS pseudo-ion at *m/z* 427.1734 [M + Na]^+^. The ^1^H NMR spectrum of **10** showed four aromatic hydrogens as two similar systems, one at δ_H_ 6.69 (2H, br s), and another at 6.63 (2H, br s), indicating the presence of two 1,3,4,5-tetrasubstituted benzene rings (Table 4). The chemical shifts observed for aromatic hydrogens along with the presence of four singlets corresponding to methoxy hydrogens at δ_H_ 2 × 3.84 and 2 × 3.86 (s, 3H each) indicated the presence of 3,5-dimethoxy-4-hydroxyphenyl groups in this compound. The ^13^C NMR data corroborated the structural determination of these aromatic rings. Moreover, ^1^H NMR spectral data suggested a nonsymmetric tetrahydrofuran lignan, through signals corresponding to two methyl groups at δ_H_ 1.00 (d, *J* = 6.4 Hz) and 0.63 (d, *J* = 7.0 Hz) in addition to two oxybenzyl methines at δ_H_ 4.64 (d, *J* = 9.3) and 5.47 (d, *J* = 4.4 Hz). The attachment of aromatic rings to the tetrahydrofuran ring was determined by an HMBC experiment. In the HMBC spectrum, interactions were observed between H-2′ and C-7′ and C-4′, between H-6′/C-4′ and C-2′, and between H-2 and C-7. These data indicated the presence of the two 3,5-dimethoxy-4-hydroxyphenyl structural parts at C-7 and C-7′, respectively. Based on previous literature data [14,15], the coupling constant of 9.3 Hz for the doublet at δ_H_ 4.64 (H-7) indicated that this hydrogen was in a *trans* configuration with the adjacent H-8, while the coupling constant of 4.4 Hz of H-7′ demonstrated its *cis* relationship with H-8′. NOE correlations confirmed the relative stereochemistry at the tetrahydrofurane ring as *trans* (C-7/C-8), *cis* (C-8/C-8′), and *cis* (C-8′/C-7′). Moreover, NOE effects proved the *cis* configuration of the aromatic group at C-7′ and methyl groups at C-9 and C-9′. These data permitted the establishment of the structure of the new tetrahydrofuran lignan **10** as *rel*-(7*S*,8*R*,7′*S*,8′*S*)-3,5,3′,5′-tetramethoxy-4,4′-dihydroxy-7,7′-epoxylignan and named gossypilignan B.

The known compounds were identified as 12-*O*-benzoyldeacylmetaplexigenin, 9*α*-hydroxypinoresinol [16], naringenin [17], and quercitrin [18]. All compounds were isolated for the first time from the plant. Such polyoxypregnane ester derivatives were isolated previously, mainly from Asclepiadaceae species (e.g., *Cynanchum wilfordi* and *Leptadenia hastata*) [9]. Though *Euphorbia* species are frequently characterized by the abundant presence of various terpenoids, this is only the second report of pregnanes from a plant belonging to the Euphorbiaceae family. Previously, two pregnane glycosides were identified from the aerial parts of *Croton ruizianus*. Their aglycon was 3*β*,14*β*,15*β*,16*α*-tetrahydroxypregnan-20-one, while the connecting sugar parts were either 3-*O*-*β*-d-Glu-(1→4)-*β*-d-Ole-(1→4)-*β*-d-Ole-(1→4)-*β*-d-Dig-(1→4)-*β*-d-Ole or 3-*O*-*β*-d-Ole-(1→4)-*β*-d-Ole-(1→4)-*β*-d-Dig-(1→4)-*β*-d-Ole (Ole—oleandrose; Dig—digitoxose) [19]. Pregnane glycosides isolated from *E. gossypina* var. *coccinea* were substituted with thevetose, cymarose, digitoxose, and glucose-containing linear sugar chains.

The 7,7-diarylbutanol *seco*-lignans are an interesting class of lignans with both chemical and pharmacological importance. Cytotoxic, anti-HIV-1, and antioxidant activities have been previously reported for this class of natural products [12,20,21].

Pregnane glycosides are reported to possess various biological activities, an antiproliferative effect being one of the most important ones [22]. Amplexoeside A, isolated from an Asclepiadaceae species *Asclepias amplexcicaulis*, showed antiproliferative activity in the KB assay. Condurangoglycoides identified from *Marsdenia congurango* were proved to be active against Sarcoma 180 and Ehrlich sarcoma in mice [23,24]. Pregnane glycosides (Ap and Ao) from *Dregea uolubilis* showed activity against Ehrlich carcinoma [25]. Moreover, compounds with an antitumor effect were isolated from *Aspidopterys obcordata*, *Caralluma dalzielii*, *C. negevensis*, *C. quadrangular*, *Cynanchum wilfordi*, *Desmidorchis flava*, *Gelsemium sempervirens*, *Marsdenia tenacissima*, *Periploca sepium*, *Solenostemma argel*, and *Stizophyllum riparium* [8,26,27,28]. Although the molecular backbone of lignans consists of only two phenylpropane (C6–C3) units, they show an enormous structural diversity, and promising pharmacological effects. A considerable number of plant lignans (e.g., podophyllotoxin, sesamin, diphyllin, enterolactone, arctiin, matairesinol, and taxiresinol) were identified to possess antiproliferative activity [29]. Since, previously, several pregnane glycosides and lignans were reported to display strong antiproliferative activities, all isolated compounds belonging to these groups of special metabolites were tested for their antiproliferative properties against the HeLa cell line using the MTT assay. Doxorubicin and cisplatin were used as positive controls (IC_50_s 0.02 ± 0.003 μM and 2.07 ± 0.07 μM, respectively). Among the tested compounds, only euphogossypin A (**1**) exerted weak antiproliferative activity (IC_50_ 52.4 ± 0.23 µM), while the others proved to be inactive (Table S1).

## 3. Materials and Methods

### 3.1. General Experimental Procedures

NMR spectra were recorded in CDCl_3_, CD_3_OD, and C_5_D_5_N on a Bruker Avance DRX 500 spectrometer (Billerica, MA, USA) at 500 MHz (^1^H) and 125 MHz (^13^C). The signals of the deuterated solvents were taken as reference. The chemical shift values (*δ*) were given in ppm and coupling constants (*J*) were in Hz. Two-dimensional (2D) experiments were performed with standard Bruker software. In the COSY, HSQC, and HMBC experiments, gradient-enhanced versions were used. High-resolution MS spectra were acquired on a Thermo Scientific Q-Exactive Plus Orbitrap mass spectrometer (Waltham, MA, USA) equipped with ESI ion source in positive ionization mode. The data were acquired and processed with MassLynx software.

Column chromatography (CC) was performed on polyamide (MP Biomedicals, Irvine, CA, USA). Normal-phase vacuum liquid chromatography (VLC) was carried out on silica gel (Kieselgel 60 GF_254_, 15 μm, Merck, Darmstadt, Germany). Thin-layer chromatography was performed on Kieselgel 60 RP-18 F_254_ and Kieselgel 60 F_254_ (Merck, Darmstadt, Germany). Spots were detected under UV light (245 nm and 336 nm) and made visible with vanillin sulfuric acid reagent and heating at 105 °C for 1 min. The high-performance liquid chromatography (HPLC) separation was carried out on a Waters HPLC (Waters 600 controller, Waters 600 pump, and Waters 2998 photodiode array detector), using RP (LiChrospher RP-18, 5 μm, 250 × 4 mm, Merck, Darmstadt, Germany) column. In the case of the RP-HPLC separation, the mobile phase consisted of MeOH (solvent A) and H_2_O (solvent B). The flow rate was 1 mL/min. The data were acquired and processed with the Empower software.

All solvents used for CC were of at least analytical grade (VWR Ltd., Szeged, Hungary). Ultra-pure water was prepared with a Milli-Q water purification system (Millipore, France).

### 3.2. Plant Material

Aerial parts of *Euphorbia gossypina* var. *coccinea* Pax. were collected in Kenya (GPS coordinates 1°24′24.31777″ S, 36°42′53.86125″ E), Africa, in July 2018, and identified by Patrick Chalo Mutiso, a taxonomist (Department of Biological Sciences, Faculty of Science and Technology, University of Nairobi). A voucher specimen (no. UON 2018/249) was deposited at the Herbarium of the School of Biological Sciences, University of Nairobi, Kenya.

### 3.3. Isolation of Compounds

The dried and ground aerial parts of *E. gossypina* var. *coccinea* (1 kg) were extracted with methanol at room temperature. After concentration, the extract (45 g) was dissolved in 50% aqueous methanol, and solvent–solvent partitions were performed with hexane, CHCl_3,_ and EtOAc. The CHCl_3_ fraction (28.6 g) was purified by polyamide column chromatography using MeOH–H_2_O (4:1) to remove chlorophyll. Thereafter, the yielded fraction (22.8 g) was purified by VLC on silica gel with a gradient system of cyclohexane–EtOAc–EtOH (from 8:2:0 to 6:3:1) and CHCl_3_–MeOH (from 9:1 to 1:1). The TLC determination and combination of the fractions afforded 20 main fractions (Fr. 1–20). Fraction 9 (1.4 g) was further chromatographed by RP-VLC using MeOH–H_2_O gradient solvent system (8:2–2:8), and 9 fractions (Fr. 9/1–9) were obtained. Fraction 9/3 (87.9 mg) was subjected to preparative TLC using CHCl_3_–MeOH (8:2) as eluent, and was, subsequently, purified by RP-HPLC. Gradient elution was applied, starting at 10% A (methanol) and 90% B (H_2_O) for 1 min, then linearly increased to 50% A (in 10 min) to yield compound **10** (*t*_R_ = 3.1 min, 2.6 mg). Fraction 10 (1.6 g) was separated by NP-VLC using a toluene–acetone gradient system (from 9:1 to 6:4) to obtain 9 subfractions (Fr. 10/1–9). Fr. 10/4 (371 mg) was chromatographed by NP-VLC using cyclohexane–EtOAc–EtOH gradient system (from 9:1:0 to 6:3:1) to yield 6 fractions (Fr. 10/4/1–6). 9*α*-hydroxypinoresinol (5.8 mg) was isolated from Fr. 10/4/3 (87 mg) by preparative TLC using CHCl_3_–MeOH (95:5) as mobile phase. Fr. 10/5 (188 mg) was purified by RP-TLC using MeOH–H_2_O (8:2) as eluent, and further purified by RP-HPLC by using gradient elution, started at 10% A (methanol) and 90% B (H_2_O) for 1 min, then linearly increased to 50% A (in 10 min) to yield compound **9** (*t*_R_ = 7.3 min, 12.1 mg). Fr. 10/6 (123 mg) was separated by RP-TLC using MeOH–H_2_O (8:2) as solvent system and compound **1** (11.2 mg) was obtained. Fraction 11 (4.9 g) was chromatographed by NP-VLC using the gradient system of toluene–acetone (from 9:1 to 1:1) to yield 11 subfractions (Fr. 11/1–11). Fraction 11/9 (193.5 mg) was purified by RP-TLC using MeOH–H_2_O (8:2) as an eluent to yield compound **2** (19.7 mg) and two subfractions (Fr. 11/9/1 (8.1 mg) and 2 (6.9 mg)). Both fractions were purified by RP-HPLC (the gradient started at MeOH–H_2_O (2:8) for 1 min, then linearly ramped up to 1:1 in 10 min and held 0.5 min, then returned to the initial conditions within 1 min, and kept for 2 min) to yield compounds **8** (*t*_R_ = 5.2 min, 6.4 mg) and **7** (*t*_R_ = 6.1 min, 5.1 mg). Fr. 13 (165 mg) was purified by NP-TLC using CHCl_3_–MeOH (85:15) as mobile phase to isolate compound **4** (12.7 mg). Fraction 15 (5.9 g) was separated by RP-VLC using a gradient system of MeOH–H_2_O (from 1:9 to 9:1) to yield 7 subfractions (Fr. 15/1–7). Fraction 15/3 (50 mg) was subjected to an NP-TLC using toluene–acetone (1:1) as an eluent to obtain naringenin (5 mg). Fraction 15/6 (75 mg) was subjected to RP-TLC using MeOH–H_2_O (7:3) to yield quercitrin (8.9 mg). Fraction 15/7 (329 mg) was purified by RP-HPLC using MeOH–H_2_O (8:2) isocratic solvent system to obtain 12-*O*-benzoyldeacylmetaplexigenin (*t*_R_ = 11 min, 47.1 mg). Fraction 15/8 (75 mg) was purified by RP-HPLC (the gradient started at MeOH–H_2_O (2:8) for 1 min, then linearly ramped up to 1:1 in 10 min and held 0.5 min, then returned to the initial conditions within 1 min and kept for 2 min) to yield compound **6** (*t*_R_ = 5.5 min, 13.9 mg). Fr. 16 (1.6 g) was subjected to an NP-VLC using CHCl_3_–MeOH gradient system (from 98:2 to 1:1) to yield 9 subfractions (Fr. 16/1–9). Fr. 16/6 (106 mg) was further purified by RP-HPLC using MeOH–H_2_O (7:3) isocratic solvent system to obtain compound **5** (*t*_R_ = 7.2 min, 24.3 mg).

#### 3.3.1. Euphogossypin A (**1**)

White amorphous powder; [α${]}_{D}^{25}$ +12 (c 0.1, MeOH); HRESIMS *m/z*: 955.4660 [M + Na]^+^ (calcd for C_49_H_72_O_17_Na, 955.4662); ^1^H (CDCl_3_, 500 MHz) and ^13^C NMR (CDCl_3_, 125 MHz) data, see Table 1.

#### 3.3.2. Euphogossypin B (**2**)

White amorphous powder; [α${]}_{D}^{25}$ +24 (c 0.2, MeOH); HRESIMS *m/z*: 927.4352 [M + Na]^+^ (calcd for C_47_H_68_O_17_Na, 927.4349); ^1^H (C_5_D_5_N, 500 MHz) and ^13^C NMR ((C_5_D_5_N, 125 MHz) data, see Table 1.

#### 3.3.3. Euphogossypin C (**3**)

White amorphous powder; [α${]}_{D}^{25}$ +5 (c 0.1, MeOH); HRESIMS *m/z*: 941.4508 [M + Na]^+^ (calcd for C_48_H_70_O_17_Na 941.4510); ^1^H (CD_3_OD, 500 MHz) and ^13^C NMR (CD_3_OD, 125 MHz) data, see Table 1

#### 3.3.4. Euphogossypin D (**4**)

White amorphous powder; [α${]}_{D}^{25}$ +63 (c 0.15, MeOH); HRESIMS *m/z*: 1103.5029 [M + Na]^+^ (calcd for C_54_H_80_O_22_Na, 1103.5033); ^1^H (CD_3_OD, 500 MHz) and ^13^C NMR (CD_3_OD, 125 MHz) data, see Table 2.

#### 3.3.5. Euphogossypin E (**5**)

White amorphous powder; [α${]}_{D}^{25}$ 0 (c 0.15, MeOH); HRESIMS *m/z*: 1089.4869 [M + Na]^+^ (calcd for C_53_H_78_O_22_Na, 1089.4877); ^1^H (CD_3_OD, 500 MHz) and ^13^C NMR (CD_3_OD, 125 MHz) data, see Table 2.

#### 3.3.6. Euphogossypin F (**6**)

White amorphous powder; [α${]}_{D}^{25}$ +3 (c 0.15, MeOH); HRESIMS *m/z*: 1103.5029 [M + Na]^+^ (calcd for C_54_H_80_O_22_Na, 1103.5033); ^1^H (CD_3_OD, 500 MHz) and ^13^C-NMR (CD_3_OD, 125 MHz) data, see Table 2.

#### 3.3.7. Euphogossypin G (**7**)

White amorphous powder; [α${]}_{D}^{25}$ +12 (c 0.05, MeOH); HRESIMS *m/z*: 865.4188 [M + Na]^+^ (calcd for C_42_H_66_O_17_Na, 865.4192); ^1^H (CDCl_3_, 500 MHz) and ^13^C NMR (CDCl_3_, 125 MHz) data, see Table 3.

#### 3.3.8. Euphogossypin H (**8**)

White amorphous powder; [α${]}_{D}^{25}$ +33 (c 0.15, MeOH); HRESIMS *m/z*: 837.4238 [M + Na]^+^ (calcd for C_41_H_66_O_16_Na, 837.4243); ^1^H (CDCl_3_, 500 MHz) and ^13^C NMR (CDCl_3_, 125 MHz) data, see Table 3.

#### 3.3.9. Gossypilignan A (**9**)

Pale yellow amorphous solid; [α${]}_{D}^{25}$ −13 (c 0.1, MeOH); HRESIMS *m/z*: 429.1889 [M + Na]^+^ (calcd for C_22_H_30_O_7_Na, 429.1884); ^1^H (CD_3_OD, 500 MHz) and ^13^C NMR (CD_3_OD, 125 MHz) data, see Table 4.

#### 3.3.10. Gossypilignan B (**10**)

White amorphous powder; [α${]}_{D}^{25}$ −5 (c 0.05, MeOH); HRESIMS *m/z*: 427.1734 [M + Na]^+^ (calcd for C_22_H_28_O_7_Na, 427.1727); ^1^H (CD_3_OD, 500 MHz) and ^13^C NMR (CD_3_OD, 125 MHz) data, see Table 4.

#### 3.3.11. 12-*O*-Benzoyldeacylmetaplexigenin

^1^H NMR (in CDCl_3_) δ_H_ 1.15 (3H, s, Me-19), 1.15, 1.90 (2H, m, H-1), 1.55 (3H, s. Me-18), 1.56, 1.83 (2H, m, H-2), 1.60 (1H, m H-9), 1.96 (2H, m, H-11), 2.03 (2H, m, H-15), 2.07 (3H, s, Me-21), 2.25 (2H, m, H-7), 2.30–2.39 (2H, m, H-4), 1.92, 2.86 (2H, m, H-16), 3.57 (1H, m, H-3), 4.86 (1H, dd, *J* = 10.2, 5.6 Hz, H-12), and 5.39 (1H, br s, H-6); ^13^C NMR see Table 1.

### 3.4. Antiproliferative Assays

#### 3.4.1. Cell Line

Human cervix carcinoma (HeLa) cells were cultured in Eagle’s Minimal Essential Medium (EMEM, containing 4.5 g/L glucose) supplemented with a non-essential amino acid mixture, a selection of vitamins, and 10% heat-inactivated fetal bovine serum. The cell line was detached with 0.25% trypsin and 0.02% EDTA for 5 min at 37 °C. The cell line was purchased from LGC Promochem, Teddington, England.

#### 3.4.2. Antiproliferative Assay

The antiproliferative assay of the isolated compounds (**1**–**3**, **5**–**12**) against the human cervix carcinoma (HeLa) cell line was performed by MTT, using cisplatin and doxorubicin as positive controls. This assay was carried out as previously described [30,31].

## 4. Conclusions

In this article, nine polyoxypregnane ester derivatives, including euphogossypins A–H (**1**–**8**) as new natural products, two new lignans (gossypilignans A (**9**) and B (**10**)), and 12-*O*-benzoyldeaxcylmetaplexigenin (**11**), 9α-hydroxypinoresinol (**12**), naringenin (**13**), and quercitrin (**14**) as known compounds, were characterized from the aerial parts of *E. gossypina* var. *coccinea*. Their planar structures were elucidated by comprehensive spectroscopic data. All compounds were isolated for the first time from the plant. Although previously several pregnane glycosides and lignans have been found to be active as antiproliferative agents, none of the compounds isolated from *E. gossypina* var. *coccinea* showed remarkable antiproliferative activity against the Hela cells. Our findings enrich the knowledge of special metabolites of *Euphorbia* species.

## Figures and Tables

**Table 1:** The ^1^H and ^13^C NMR data for compounds **1**–**3**.

Atom	1 *		2 *		3 *	
	**δ_H_ (*J* in Hz)**	**δ_C_**	**δ_H_ (*J* in Hz)**	**δ_C_**	**δ_H_ (*J* in Hz)**	**δ_C_**
1	1.13, m, α/1.89, m, β	38.9, CH_2_	1.14, m, α/1.83, m, β	39.4, CH_2_	1.14, m, α/1.83 m, β	39.8, CH_2_
2	1.62, m, β/1.92, m, α	29.1, CH_2_	1.82, m, β/2.11, m, α	30.3, CH_2_	1.58, m, β/1.87 m, α	30.2, CH_2_
3	3.57, m	78.0, CH	3.89, m	78.1, CH	3.54, m	79.3, CH
4	2.29, m, β/2.40, m, α	38.9, CH_2_	2.43, m, * β/2.55, m, α	39.7, CH_2_	2.22, m; 2.38 dd (3.4, 12.7)	39.8, CH_2_
5	-	140.8, C	-	139.9, C	-	140.3, C
6	5.38, br s	117.8, CH	5.31, br s	119.6, CH	5.36, br d (4.7)	119.7, CH
7	2.22, m	34.4, CH_2_	2.38, m/2.50, m	35.2, CH_2_	2.12–2.22 m	35.2, CH_2_
8	-	74.4, C	-	74.8, C	-	75.2, C
9	1.59, m	43.8, CH	1.80, m	44.9, CH	1.59 m	45.1, CH
10	-	37.3, C	-	37.9, C	-	38.2, C
11	1.94, m	24.3, CH_2_	2.21, m, α/2.36, m, β	25.5, CH_2_	1.81, m, α/2.00, m, β	25.5, CH_2_
12	4.83, dd (4.2, 11.9)	73.3, CH	5.36, dd (4.3, 11.5)	77.5, CH	4.83 dd (4.3, 11.9)	74.7, CH
13	-	58.5, C	-	58.8, C	-	59.1, C
14	-	88.1, C	-	90.0, C	-	90.0, C
15	2.03, m	33.4, CH_2_	2.14–2.21, m	34.3, CH_2_	1.92, m; 2.06, * m	34.3, CH_2_
16	1.92, m, β/2.85, m, α	32.1, CH_2_	2.07, m/3.27, m	33.7, CH_2_	1.73, m, β/2.87, m, α	33.5, CH_2_
17	-	91.6, C	-	93.0, C	-	93.1, C
18	1.54, s	9.7, CH_3_	2.10, s	11.3, CH_3_	1.67, s	10.6, CH_3_
19	1.12, s	18.7, CH_3_	1.34, s	18.6, CH_3_	1.16, s	18.6, CH_3_
20	-	209.5, C	-	210.6, C	-	212.2, C
21	2.06, s	27.5, CH_3_	2.37, s	28.2, CH_3_	2.05, s	27.8, CH_3_
Bz						
1′	-	165.5, C	-	165.8, C	-	166.7, C
2′	-	130.1, C	-	131.8, C	-	131.6, C
3′,7′	7.93, dd (1.1, 8.2)	129.7, CH	8.31, d (7.8)	130.4, CH	7.95, d (7.9)	130.5, CH
4′,6′	7.43, t (8.0)	128.6, CH	7.49, t (7.7)	129.4, CH	7.48, dd (7.9, 7.4)	129.6, CH
5′	7.55, t (8.1)	133.3, CH	7.59, t (7.7)	133.7, CH	7.61, t (7.4)	134.3, CH
	**Cym I**		**Dig I**		**Cym**	
1	4.85, dd (2.0, 9.0)	96.2, CH	5.48, d (9.4)	96.9, CH	4.87, dd (1.9, 8.9)	97.2, CH
2	1.58, m, a/2.08, m, e	35.7, CH_2_	2.05, m; 2.43, m *	39.5, CH_2_	1.54, m, a/2.06, m, * e	36.7, CH_2_
3	3.80, m	77.2, * CH	4.65, m	68.0, CH	3.85, m	78.6, CH
4	3.21, dd (3.0, 9.6)	82.7, CH	3.53, m	83.9, CH	3.24, m *	83.9, CH
5	3.84, m	68.7, CH	4.31, m	69.1, CH	3.81, m	70.0, CH
6	1.22, d (6.3)	18.3, CH_3_	1.45, d (6.0)	19.1, CH_3_	1.20, d (6.2)	18.5, CH_3_
3-OMe	3.42, s *	58.1, ^#^ CH_3_			3.44, s	58.5, CH_3_
	**Cym II**		**Dig II**		**Dig**	
1	4.76, dd (1.8, 9.5)	99.7, CH	5.41, d (9.6)	100.3, CH	4.89, dd (1.7, 9.1)	101.0, CH
2	1.65, m, a/2.16, m, e	35.3, CH_2_	2.00, m, a/2.43, m, * e	39.4, CH_2_	1.71, m, a/2.02, m, e	38.8, CH_2_
3	3.78, m	77.1,* CH	4.70, m	68.3, CH	4.21, m	68.6, CH
4	3.26, dd (2.9, 9.6)	82.8, CH	3.60, m	84.1, CH	3.24, * m	83.8, CH
5	3.90, m	68.4, CH	4.37, m	69.5, CH	3.87, m	69.5, CH
6	1.27, d (6.2)	18.6, CH_3_	1.59, d (6.1)	19.0, CH_3_	1.31, d (6.2)	18.6, CH_3_
3-OMe	3.44, s *	58.2, ^#^ CH_3_				
	**Thv**		**Thv**		**Thv**	
	4.30, d (7.8)	104.5, CH	4.82, d (7.9)	106.3, CH	4.35, d (7.9)	105.5, CH
	3.51, m	74.8, CH	3.85, m	75.3, CH	3.28, m	75.2, CH
	3.10, t (9.0)	85.4, CH	3.61, m	88.3, CH	3.01, m	87.5, CH
	3.18, t (9.2)	74.8, CH	3.58, m	76.3, CH	3.04, m	76.5, CH
	3.36, dd (6.2, 9.2)	71.8, CH	3.73, m	73.2, CH	3.31, ^+^ m	73.2, CH
	1.31, d (6.2)	17.9, CH_3_	1.53, d (6.0)	18.9, CH_3_	1.25, d (6.1)	18.1, CH_3_
3-OMe	3.65, s	60.8, CH_3_	3.91, s	61.4, CH_3_	3.63, s	61.1, CH_3_

* in CDCl_3_ (**1**), pyridine-*d*_5_ (**2**), methanol-*d*_4_ (**3**), ^#^ interchangeable signals; ^+^ overlaps with solvent signal.

**Table 2:** The ^1^H and ^13^C NMR data for compounds **4**–**6** in CD_3_OD.

Atom	4		5		6	
	**δ_H_ (*J* in Hz)**	**δ_C_**	**δ_H_ (*J* in Hz)**	**δ_C_**	**δ_H_ (*J* in Hz)**	**δ_C_**
1	1.14, m, α/1.85, m, β	39.8, * CH_2_	1.16, m, α/1.85 m, β	39.8, CH_2_	1.16, m, α/1.84, m, β	39.8, CH_2_
2	1.60, m, * β/1.88, m, α	30.2, CH_2_	1.61, m, * β/1.88 m, α	30.1, CH_2_	1.59, m, β/1.87, m, α	30.2, CH_2_
3	3.55, m	79.3, CH	3.56, m	79.3, CH	3.54, m	79.3, CH
4	2.23, m, β/2.38, m, α	39.8, * CH_2_	2.23, m; 2.40 dd (3.5, 12.7)	39.8, CH_2_	2.22, m, β/2.38, m, α	39.8, CH_2_
5	-	140.2, C	-	140.3, C	-	140.3, C
6	5.37, br d (4.6)	119.7, CH	5.37, br d (4.6)	119.6, CH	5.36, br s	119.7, CH
7	2.13–2.22, m	35.2, CH_2_	2.14–2.22 m	35.2, CH_2_	2.14–2.21, m	35.2, CH_2_
8	-	75.0, C	-	75.0, C	-	75.0, C
9	1.61, m *	45.1, CH	1.61 m *	45.1, CH	1.60, m	45.1, CH
10	-	38.2, C	-	38.2, C	-	38.2, C
11	1.83, m, α/2.02, m, β	25.5, CH_2_	1.83, m, α/2.02, m, β	25.4, CH_2_	1.82, m, α/2.01, m, β	25.4 CH_2_
12	4.83, dd ^+^	74.7, CH	4.82 dd (4.3, 11.9)	74.7, CH	4.82, dd (overlaps)	73.3, CH
13	-	59.1, C	-	59.1, C	-	59.1, C
14	-	90.0, C	-	90.0, C	-	90.0, C
15	1.94, m; 2.07, m	34.3, CH_2_	1.94, m; 2.08, m	34.3, CH_2_	1.93, m; 2.07, m	34.3, CH_2_
16	1.75, m, β/2.87, m, α	33.5, CH_2_	1.75, m, β/2.87, m, α	33.5, CH_2_	1.74, m, β/2.87, m, α	33.5, CH_2_
17	-	93.1, C	-	93.1, C	-	93.1, C
18	1.67, s	10.6, CH_3_	1.67, s	10.6, CH_3_	1.67, s	10.6, CH_3_
19	1.16, s	18.6, CH_3_	1.16, s	18.6, CH_3_	1.16, s	18.6, CH_3_
20	-	212.3, C	-	212.2, C	-	212.2, C
21	2.06, s	27.8, CH_3_	2.06, s	27.8, CH_3_	2.05, s	27.8, CH_3_
Bz						
1′	-	166.7, C	-	166.7, C	-	166.7, C
2′	-	131.5, C	-	131.5, C	-	131.5, C
3′,7′	7.95, d (7.9)	130.5, CH	7.95, d (7.9)	130.5, CH	7.95, d (8.2)	130.5, CH
4′,6′	7.48, t (7.8)	129.6, CH	7.48, t (7.9)	129.5, CH	7.48, t (7.9)	129.6, CH
5′	7.60, t (7.5)	134.3, CH	7.61, t (7.8)	134.3, CH	7.60, t (7.9)	134.3, CH
	**Cym I**		**Dig I**		**Cym**	
1	4.87, dd (1.6, 9.6)	97.2, CH	4.96, dd (1.7, 9.7)	97.0, CH	4.87, dd (1.9, 8.9)	97.2, CH
2	1.55, m, a/2.07, m, * e	36.6, CH_2_	1.68, m, a/1.96, m, e	38.9, CH_2_	1.54, m, a/2.06, m, e	36.7, CH_2_
3	3.85, m ^#^	78.6, CH	4.24, m *	68.4, CH	3.85, m	78.6, CH
4	3.24, m *	83.9, CH	3.23, m *	83.7, CH	3.26, m *	83.8, * CH
5	3.81, m	70.0, CH	3.81, m	69.5, CH	3.82, m	70.0, CH
6	1.19, d (6.3)	18.5,^#^ CH_3_	1.21, d (6.2)	18.5, * CH_3_	1.20, d (6.1)	18.5, CH_3_
3-OMe	3.43, s ^#^	58.4, CH_3_			3.44, s	58.5, CH_3_
	**Cym II**		**Dig II**		**Dig**	
1	4.80, m ^+^	101.1, CH	4.93, dd (1.6, 9.7)	100.4, CH	4.89, dd (1.7, 9.1)	101.0, CH
2	1,59, m, a/2.14, m, * e	36.4, CH_2_	1.76, m, a/2.03, m, e	38.7, CH_2_	1.71, m, a/2.02, m, e	38.8, CH_2_
3	3.84, m *	78.6, CH	4.22, m *	68.5, CH	4.22, m	65.6, CH
4	3.28, m	84.1, CH	3.27, m	83.8, CH	3.26, m *	83.9, * CH
5	3.88, m *	70.1, CH	3.91, m	69.7, CH	3.88, m	69.5, CH
6	1.30, d (6.3)	18.7, CH_3_	1.31, d (6.2)	18.5, CH_3_	1.31, d (6.2)	18.6, CH_3_
3-OMe	3.44, s ^#^	58.6, CH_3_				
	**Thv**		**Thv**		**Thv**	
	4.34, d (7.8)	106.1, CH	4.37, d (7.8)	105.5, CH	4.37, d (7.8)	105.5, CH
	3.30, m	75.0, CH	3.34, m	74.7, CH	3.33, m	74.8, CH
	3.19, m *	86.1, CH	3.20, m	86.0, CH	3.19, m	86.0, CH
	3.37, m	82.8, CH	3.38, m	82.8, CH	3.37, m	82.8, CH
	3.47, m	72.5, CH	3.48, m	72.6, CH	3.48, m	72.6, CH
	1.37, d (6.1)	18.5, ^#^ CH_3_	1.36, d (6.2)	18.6, CH_3_	1.36, d (6.2)	18.5, CH_3_
3-OMe	3.63, s	61.2, CH_3_	3.63, s	61.3, CH_3_	3.62, s	61.3, CH_3_
	**Glc**		**Glc**		**Glc**	
1	4.43, d (7.7)	104.3, CH	4.43, d (7.8)	104.3, CH	4.43 d (7.8)	104.3, CH
2	3.18, m *	75.7, CH	3.18, m	75.7, CH	3.18, m	75.7, CH
3	3.35, m	78.0, CH	3.35, m	78.0, CH	3.35, m	78.0, CH
4	3.23, m *	71.9, CH	3.23, m *	71.9, CH	3.22, m	71.9, CH
5	3.26, m	78.4, CH	3.26, m	78.4, CH	3.26, m *	78.4, CH
6	3.64, dd (6.4, 12.0); 3.87, m	63.2, CH_2_	3.64, dd (6.4, 12.0); 3.87, dd (2.0, 12.0)	63.2, CH_2_	3.64, m; 3.87, m	63.2, CH_2_

*^,^^#^ interchangeable signals; ^+^ overlaps with residual water.

**Table 3:** The ^1^H and ^13^C NMR data for compounds **7** and **8** in CDCl_3_.

Atom	7		8	
	**δ_H_ (*J* in Hz)**	**δ_C_**	**δ_H_ (*J* in Hz)**	**δ_C_**
1	1.10, m, α/1.86, m, β	39.0, * CH_2_	1.08, m, α/1.87, m, β	39.0, * CH_2_
2	1.64, m, β/1.93, m, * α	29.0, CH_2_	1.66, m, β/1.94, m, α	29.2, CH_2_
3	3.57, m	78.1, CH	3.55, m	78.1, CH
4	2.29, m, β/2.41, m, α	39.0, * CH_2_	2.30, m, β/2.38, m, α	39.1, * CH_2_
5	-	141.4, C	-	140.8, C
6	5.35, br s	117.5, CH	5.36, br s	117.9, CH
7	2.20, m	34.3, CH_2_	2.18, m	34.5, CH_2_
8	-	74.7, C	-	74.5, C
9	1.52, m	43.9, CH	1.46, dd (3.2, 13.1)	44.4, CH
10	-	37.4, C	-	37.3, C
11	1.78, m	24.4, CH_2_	1.60, m, α/1.90, m, β	28.2, CH_2_
12	4.51,dd (5.8, 10.3)	72.7, CH	5.68, m	69.7, CH
13	-	57.8, C	-	61.1, C
14	-	88.3, C	-	88.0, C
15	1.93, m *	32.8, CH_2_	1.94, m *	34.3, CH_2_
16	1.83, m, β/2.87, m, α	32.4, CH_2_	1.92, m, β/2.75, m, α	33.7, CH_2_
17	-	91.9, C	-	92.1, C
18	1.42, s	9.4, CH_3_	1.27, s	7.9, CH_3_
19	1.12, s	18.9, CH_3_	1.16, s	18.9, CH_3_
20	-	209.4, C	-	213.9, C
21	2.24, s	27.4, CH_3_	2.34, s	28.4, CH_3_
12-OAc		170.0, C		
	1.95, s	20.8, CH_3_		
14-OH	3.94, s		4.12, br s	
17-OH	4.42, s		4.61, br s	
	**Dig I**		**Cym**	
1	4.92, dd (1.7, 9.3)	96.1, CH	4.85, br d (9.5) *	96.3, CH
2	1.72, m, a/2.08, m, e	37.3, CH_2_	1.59, m; 2.09, m	35.9, CH_2_
3	4.24, m *	66.7, CH	3.81, m	77.3, CH
4	3.23, dd (3.0, 9.4)	82.8, CH	3.24, dd (2.9, 9.6)	82.9, CH
5	3.79, dq (6.3, 9.4)	68.3, CH	3.85, m ^#^	68.7, CH
6	1.23, d (6.3)	18.4, CH_3_	1.22, d (6.2)	18.4, CH_3_
3-OMe			3.45, s	58.2, CH_3_
	**Dig II**		**Dig**	
1	4.91, dd (1.7, 9.3)	98.5, CH	4.85, br d (9.5) *	99.6, CH
2	1.75, m, a/2.14, m, e	37.0, CH_2_	1.77, m, a/2.15, m, * e	37.1, CH_2_
3	4.23, m *	66.8, CH	4.21, m	66.9, CH
4	3.26, dd (2.9, 9.4)	83.2, CH	3.27, dd (3.0, 9.4)	83.4, CH
5	3.90, dq (6.2, 9.4)	68.2, CH	3.86, m ^#^	67.9, CH
6	1.29, d (6.2)	18.5, CH_3_	1.29, d (6.2)	18.5, CH_3_
3-OMe				
	**Thv**		**Thv**	
	4.35, d (7.7)	103.5, CH	4.34, d (7.7)	103.5, CH
	3.47, m	74.7, CH	3.45, m	74.7, CH
	3.11, t (9.0)	85.4, CH	3.10, t (9.0)	88.3, CH
	3.19, t (9.0)	74.8, CH	3.19, t (9.0)	74.8, CH
	3.40, m	72.2, CH	3.39, dd (6.1, 9.0)	72.2, CH
	1.32, d (6.1)	17.9, CH_3_	1.31, d (6.1)	17.9, CH_3_
3-OMe	3.66, s	60.9, CH_3_	3.66, s	60.8, CH_3_

*^,^^#^ interchangeable signals.

**Table 4:** The ^1^H and ^13^C NMR data for compounds **9** and **10** in CD_3_OD.

Atom	9		10	
	**δ_H_ (*J* in Hz)**	**δ_C_**	**δ_H_ (*J* in Hz)**	**δ_C_**
1	-	137.0, C	-	136.2, C
2,6	6.66, s	106.4, CH	6.69, br s	104.7, CH
3,5	-	149.1/149.2, C	-	149.3, CH
4	-	134.7, C	-	134.7, C
7	3.52, d (11.8)	57.9, CH	4.64, d (9.3)	87.6, CH
8	2.62, m	37.2, C	2.49, m *	48.6, C
9	0.68, d (6.9)	12.1, CH	1.00, d (6.4)	12.1, CH
3,5-OMe	3.82, s/3.83, s	56.8, CH_3_	3.86, s	56.7/56.8, CH_3_
1′	-	137.8, C	-	132.5, C
2′,6′	6.64, s	106.3, CH	6.63, br s	104.3, CH
3′,5′	-	149.1/149.2, C	-	149.1, C
4′	-	134.7, C	-	135.4, C
7′	3.35, dd (6.6, 10.7) 3.45, dd (8.3, 10.7)	67.3, C	5.47, d (4.4)	86.5, CH
8′	1.77, m	37.0, CH_3_	2.48, m *	44.6, CH
9′	0.76, d (7.0) s	10.0, CH_3_	0.63, d (7.0)	9.8, CH_3_
3′,5′-OMe	3.82, s/3.83, s	56.8, C	3.84, s	56.7/56.8, CH_3_

* interchangeable signals.

## Data Availability

Data is contained within the article and Supplementary Material.

## Supplementary Materials

The following supporting information can be downloaded at: https://www.mdpi.com/article/10.3390/plants11101299/s1, Figures S1–S58: One-dimensional and two-dimensional NMR spectra of isolated compounds (**1**–**10**); Table S1: Antiproliferative activity of the isolated compounds (**1**–**3**, **5**–**12**) in HeLa cell line.
