# Supplementary material for: Polyoxypregnane Ester Derivatives and Lignans from Euphorbia gossypina var. coccinea Pax
Source: Plants (Basel). 2022 May 13;11(10):1299. doi: 10.3390/plants11101299 (PMC9146146; doi:10.3390/plants11101299)
Supplement: Supplementary file 1 [file plants-11-01299-s001.zip › plants-1714850-supplementary.pdf]

SUPPORTING INFORMATION

Polyoxypregnane Ester Derivatives and Lignans from *Euphorbia gossypina* var. *coccinea* Pax.

Reham Hammadi [1], Norbert Kúsz [1], Csilla Zsuzsanna Dávid [1], Peter Waweru Mwangi [2], Róbert Berkecz [3], Nikoletta Szemerédi [4], Gabriella Spengler [4], Judit Hohmann [1,5] and Andrea Vasas [1,*]

[1] Department of Pharmacognosy, Interdisciplinary Excellence Centre, University of Szeged, 6720 Szeged, Eötvös u. 6, Hungary; reham.hammadi@pharmacognosy.hu (R.H.); kusznorbert@gmail.com (N.K.); davidzsuzsanna88@gmail.com (C.Z.D.); hohmann.judit@szte.hu (J.H.)
[2] School of Biological Sciences, University of Nairobi, P.O. Box 30197-00100 Nairobi, Kenya; waweruk2001@gmail.com
[3] Institute of Pharmaceutical Analysis, University of Szeged, 6720 Szeged, Hungary; berkecz.robert@szte.hu
[4] Department of Medical Microbiology, Albert Szent-Györgyi Health Center and Albert Szent-Györgyi Medical School, University of Szeged, 6725 Szeged, Hungary; szemeredi.nikoletta@med.u-szeged.hu (N.S.); spengler.gabriella@med.u-szeged.hu (G.S.)
[5] Interdisciplinary Centre of Natural Products, University of Szeged, 6720 Szeged, Eötvös u. 6, Hungary; hohmann.judit@szte.hu
* Correspondence: vasas.andrea@szte.hu; Tel.: +36-62546451

TABLE OF CONTENTS

Figure S1. ^{1}H NMR spectrum of compound **1** (500 MHz, in $CDCl_3$). 4
Figure S2. ^{13}C (JMOD) NMR spectrum of compound **1** (125 MHz, in $CDCl_3$). 4
Figure S3. HSQC spectrum of compound **1** (in $CDCl_3$). 5
Figure S4. ^{1}H-^{1}H COSY spectrum of compound **1** (in $CDCl_3$). 5
Figure S5. HMBC spectrum of compound **1** (in $CDCl_3$). 6
Figure S6. NOESY spectrum of compound **1** (in $CDCl_3$). 6
Figure S7. ^{1}H NMR spectrum of compound **2** (in C_5D_5N). 7
Figure S8. ^{13}C (JMOD) NMR spectrum of compound **2** (in C_5D_5N). 7
Figure S9. HSQC spectrum of compound **2** (in C_5D_5N). 8
Figure S10. ^{1}H-^{1}H COSY spectrum of compound **2** (in C_5D_5N). 8
Figure S11. HMBC spectrum of compound **2** (in C_5D_5N). 9
Figure S12. NOESY spectrum of compound **2** (in C_5D_5N). 9
Figure S13. ^{1}H NMR spectrum of compound **3** (500 MHz, in CD_3OD). 10
Figure S14. ^{13}C (JMOD) NMR spectrum of compound **3** (125 MHz, in CD_3OD). 10
Figure S15. HSQC spectrum of compound **3** (in CD_3OD). 11
Figure S16. ^{1}H-^{1}H COSY spectrum of compound **3** (in CD_3OD). 11
Figure S17. HMBC spectrum of compound **3** (in CD_3OD). 12
Figure S18. NOESY spectrum of compound **3** (in CD_3OD). 12
Figure S19. ^{1}H NMR spectrum of compound **4** (500 MHz, in CD_3OD). 13
Figure S20. ^{13}C (JMOD) NMR spectrum of compound **4** (125 MHz, in CD_3OD). 13
Figure S21. HSQC spectrum of compound **4** (in CD_3OD). 14
Figure S22. ^{1}H-^{1}H COSY spectrum of compound **4** (in CD_3OD). 14
Figure S23. HMBC spectrum of compound **4** (in CD_3OD). 15
Figure S24. NOESY spectrum of compound **4** (in CD_3OD). 15
Figure S25. ^{1}H NMR spectrum of compound **5** (500 MHz, in CD_3OD). 16
Figure S25. ^{13}C (JMOD) NMR spectrum of compound **5** (125 MHz, in CD_3OD). 16
Figure S26. HSQC spectrum of compound **5** (in CD_3OD). 17
Figure S27. ^{1}H-^{1}H COSY spectrum of compound **5** (in CD_3OD). 17
Figure S28. HMBC spectrum of compound **5** (in CD_3OD). 18
Figure S29. NOESY spectrum of compound **5** (in CD_3OD). 18
Figure S30. ^{1}H NMR spectrum of compound **6** (500 MHz, in CD_3OD). 19
Figure S31. ^{13}C (JMOD) NMR spectrum of compound **6** (125 MHz, in CD_3OD). 19
Figure S32. HSQC spectrum of compound **6** (in CD_3OD). 20
Figure S33. ^{1}H-^{1}H COSY spectrum of compound **6** (in CD_3OD). 20
Figure S34. HMBC spectrum of compound **6** (in CD_3OD). 21
Figure S35. NOESY spectrum of compound **6** (in CD_3OD). 21

Figure S36. ^{1}H NMR spectrum of compound **7** (500 MHz, in $CDCl_3$). 22
Figure S37. ^{13}C (JMOD) spectrum of compound **7** (125 MHz, in $CDCl_3$). 22
Figure S38. HSQC spectrum of compound **7** (in $CDCl_3$). 23
Figure S39. ^{1}H-^{1}H COSY spectrum of compound **7** (in $CDCl_3$). 23
Figure S40. HMBC spectrum of compound **7** (in $CDCl_3$). 24
Figure S41. NOESY spectrum of compound **7** (in $CDCl_3$). 24
Figure S42. ^{1}H NMR spectrum of compound **8** (500 MHz, in $CDCl_3$). 25
Figure S43. ^{13}C (JMOD) NMR spectrum of compound **8** (125 MHz, in $CDCl_3$). 25
Figure S44. HSQC spectrum of compound **8** (in $CDCl_3$). 26
Figure S45. ^{1}H-^{1}H COSY spectrum of compound **8** (in $CDCl_3$). 26
Figure S46. HMBC spectrum of compound **8** (in $CDCl_3$). 27
Figure S47. NOESY spectrum of compound **8** (in $CDCl_3$). 27
Figure S48. ^{1}H NMR spectrum of compound **9** (500 Mz, in CD_3OD). 28
Figure S49. ^{13}C (JMOD) NMR spectrum of compound **9** (125 MHz, in CD_3OD). 28
Figure S50. HSQC spectrum of compound **9** (in CD_3OD). 29
Figure S51. ^{1}H-^{1}H COSY spectrum of compound **9** (in CD_3OD). 29
Figure S52. HMBC spectrum of compound **9** (in CD_3OD). 30
Figure S53. NOESY spectrum of compound **9** (in CD_3OD). 30
Figure S54. ^{1}H NMR spectrum of compound **10** (500 Mz, in CD_3OD). 31
Figure S55. ^{13}C (JMOD) NMR spectrum of compound **10** (125 MHz, in CD_3OD). 31
Figure S56. HSQC spectrum of compound **10** (in CD_3OD). 32
Figure S57. ^{1}H-^{1}H COSY spectrum of compound **10** (in CD_3OD). 32
Figure S58. HMBC spectrum of compound **10** (in CD_3OD). 33
Figure S59. NOESY spectrum of compound **10** (in CD_3OD). 33
Table S1. Antiproliferative activity of the isolated compounds (**1**-**3**, **5**-**12**) in HeLa cell line. 34

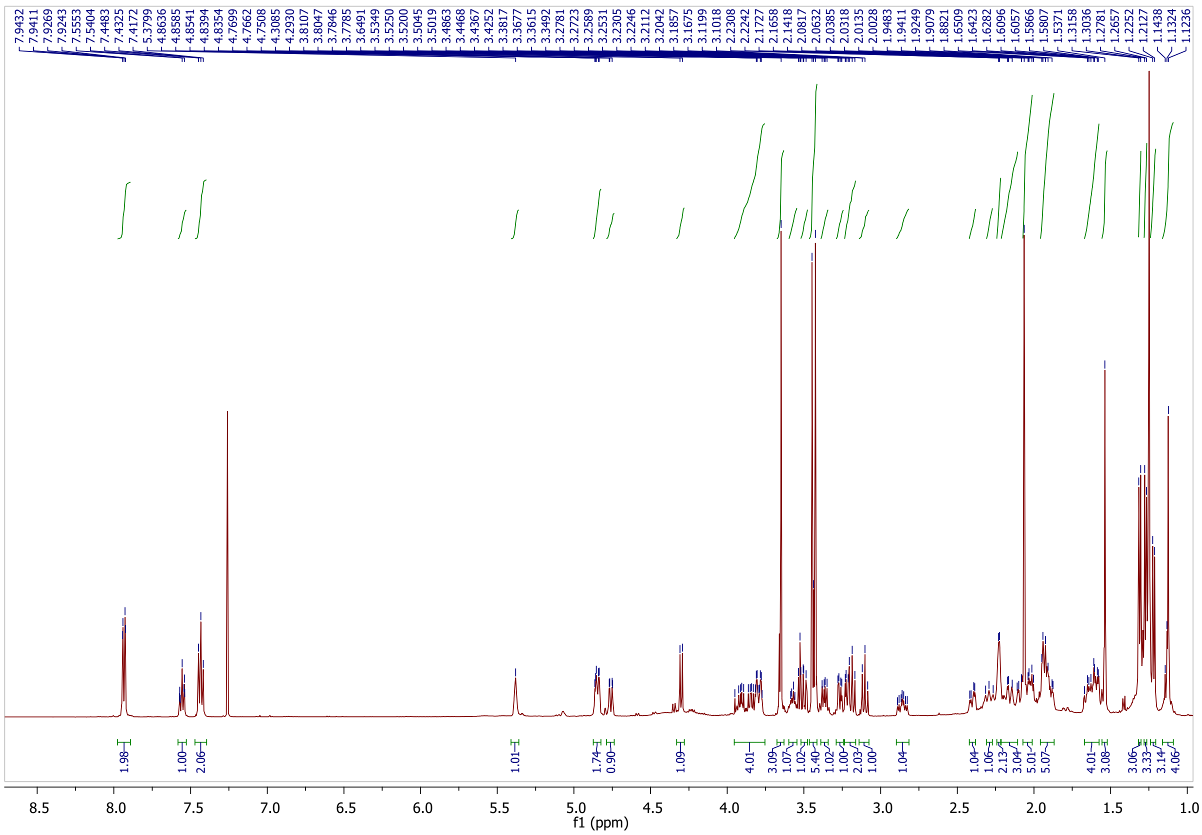

Figure S1. ^{1}H NMR spectrum of compound **1** (500 MHz, in $CDCl_3$).

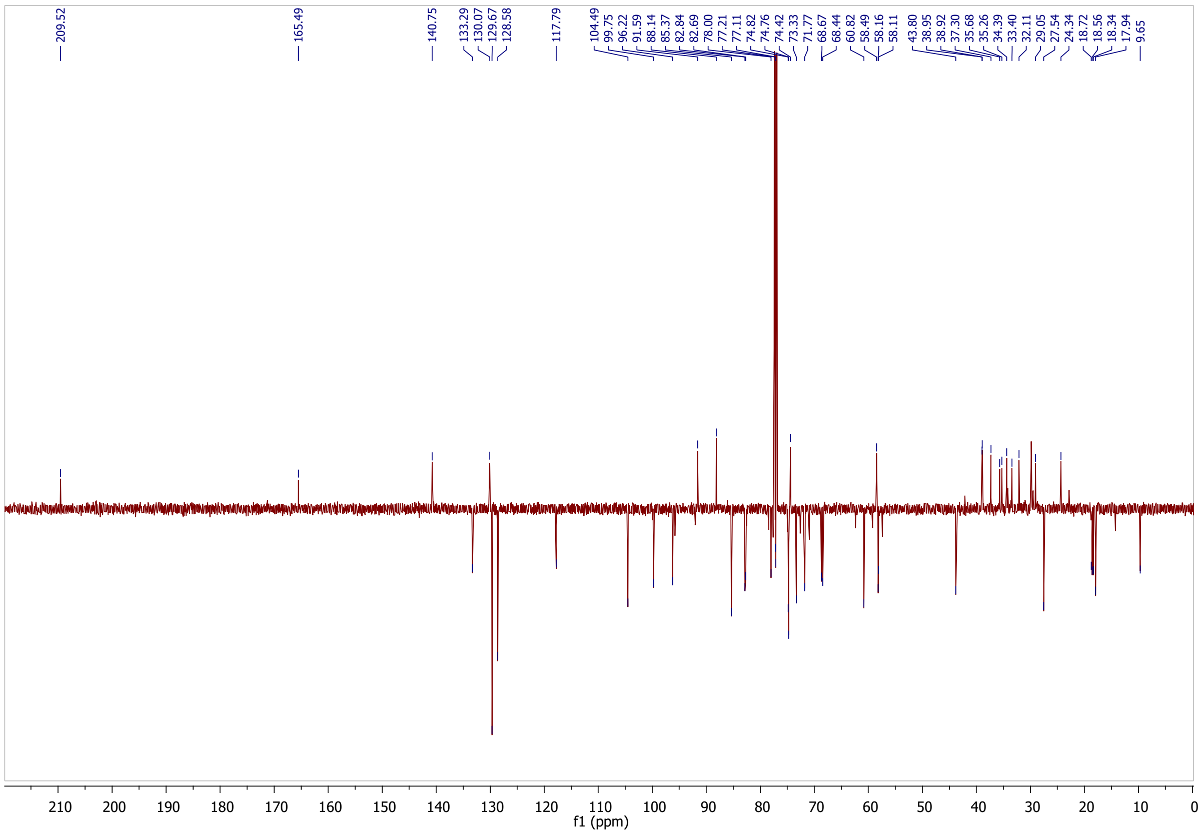

Figure S2. ^{13}C (JMOD) NMR spectrum of compound **1** (125 MHz, in $CDCl_3$).

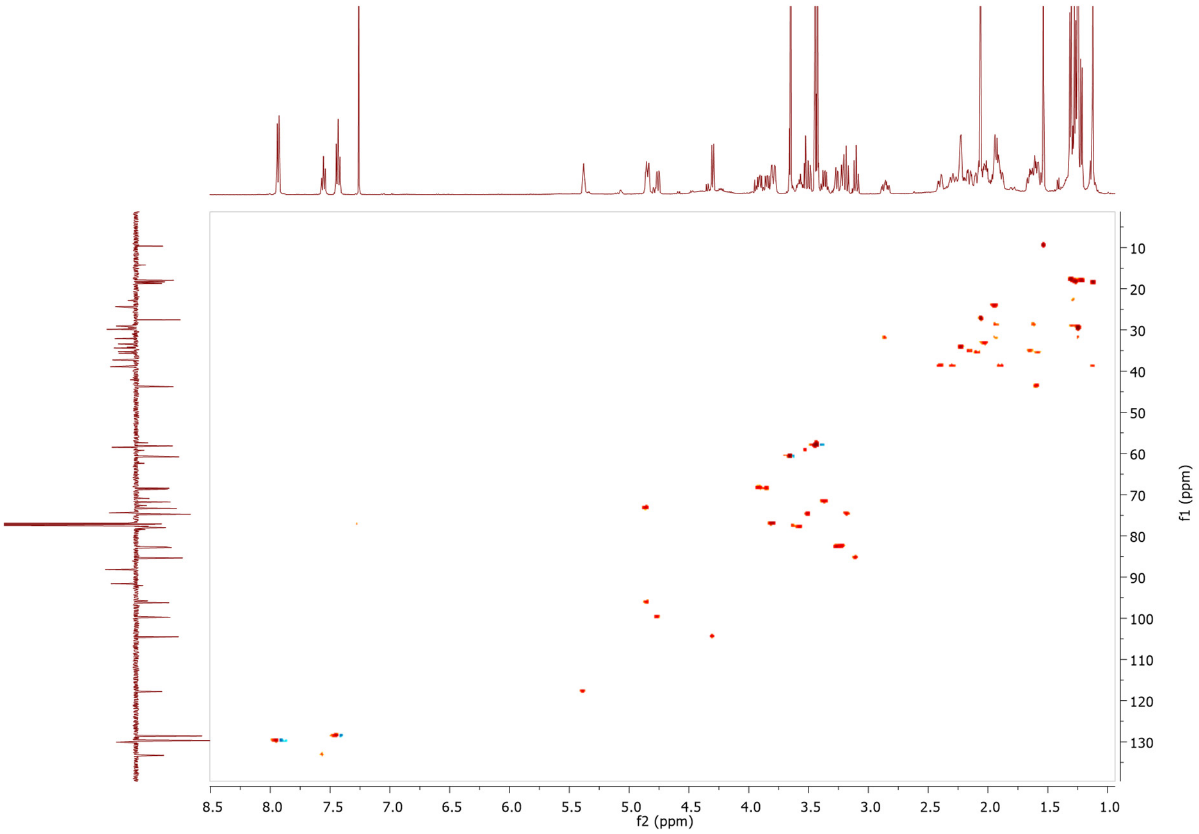

Figure S3. HSQC spectrum of compound **1** (in $CDCl_3$).

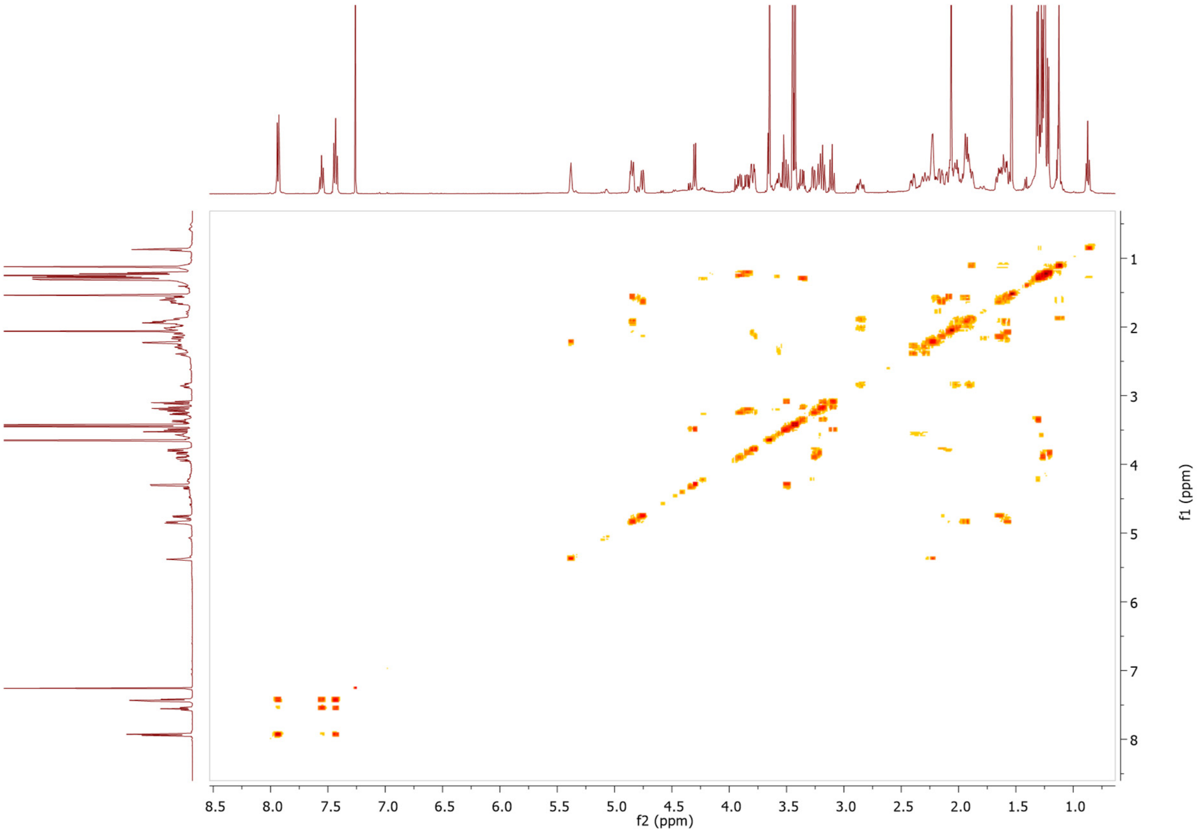

Figure S4. ^{1}H-^{1}H COSY spectrum of compound **1** (in $CDCl_3$).

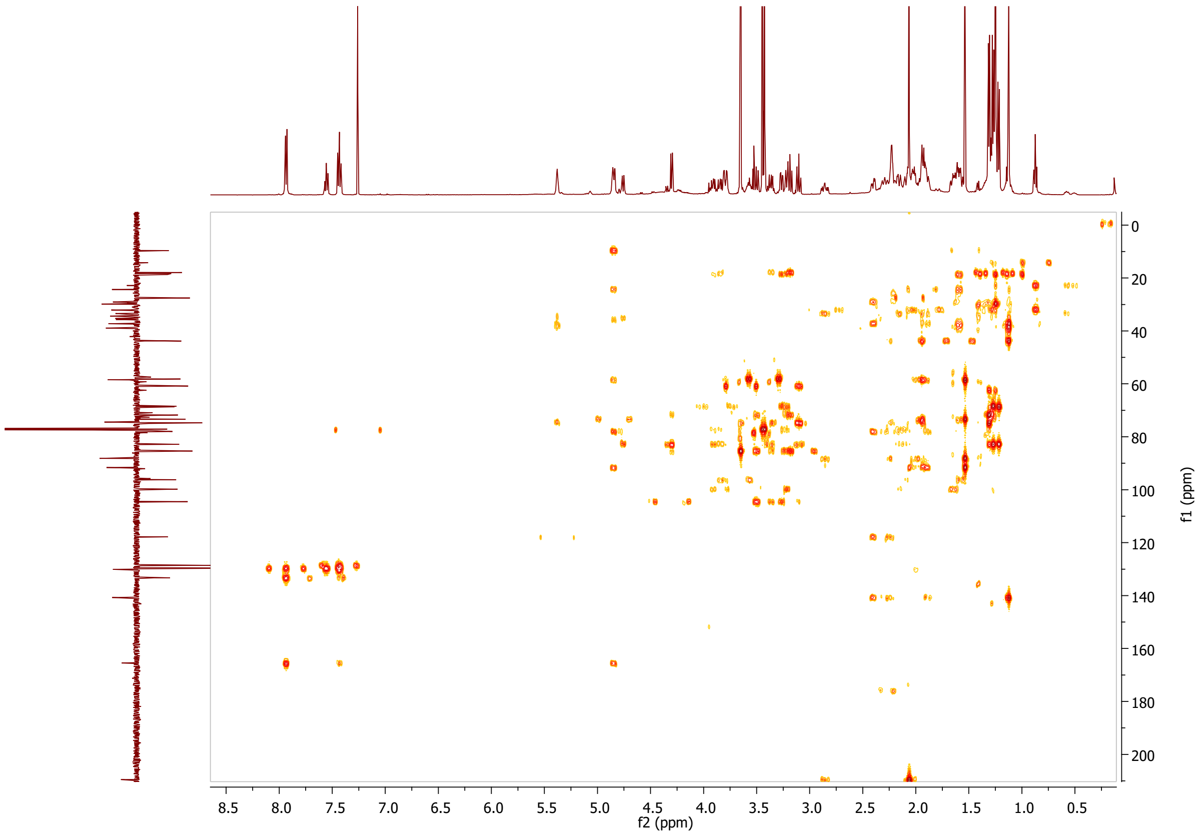

Figure S5. HMBC spectrum of compound **1** (in $CDCl_3$).

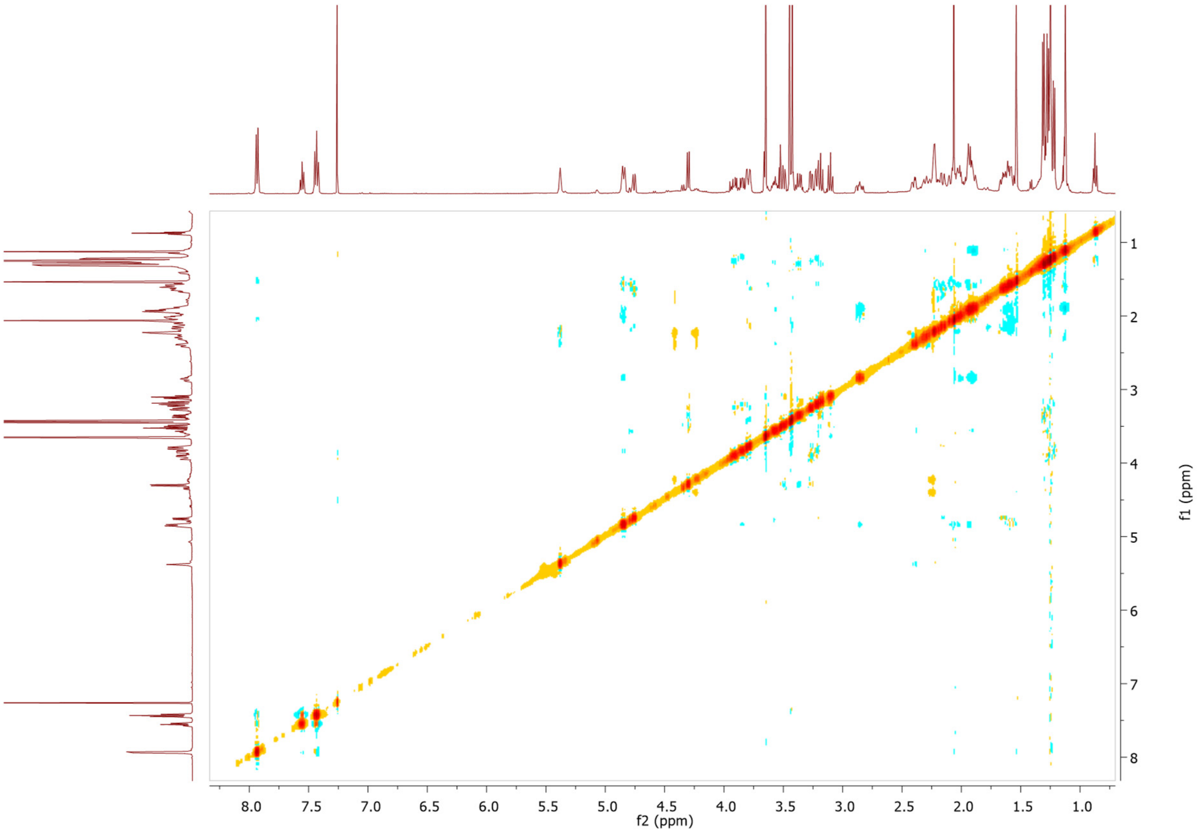

Figure S6. NOESY spectrum of compound **1** (in $CDCl_3$).

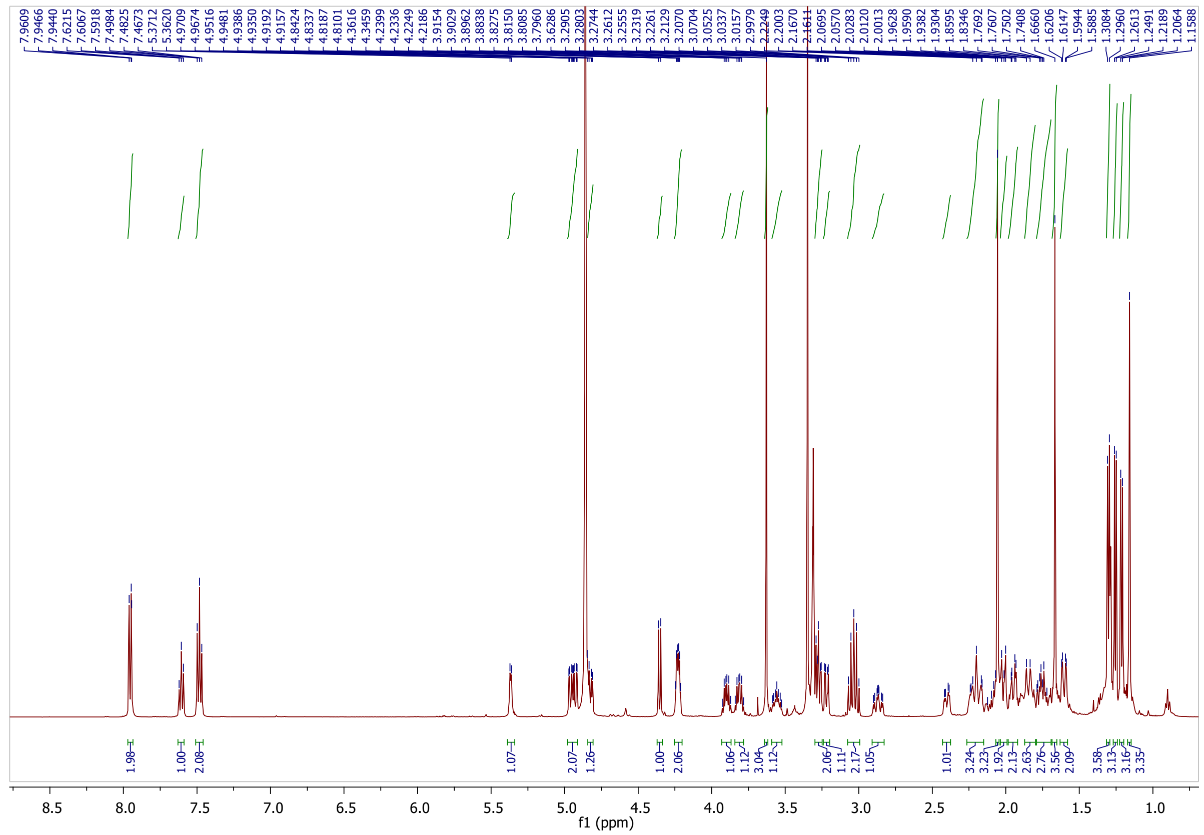

Figure S7. ^{1}H NMR spectrum of compound **2** (in C_5D_5N).

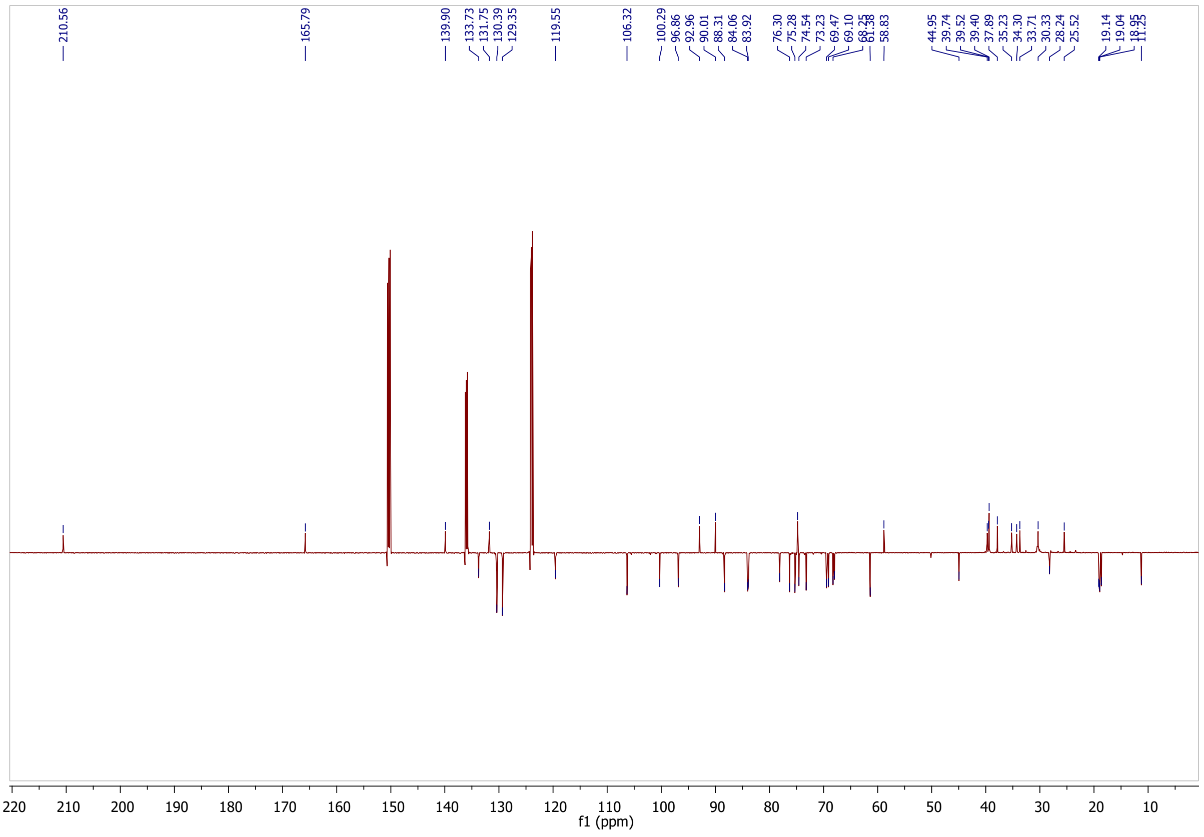

Figure S8. ^{13}C (JMOD) NMR spectrum of compound **2** (in C_5D_5N).

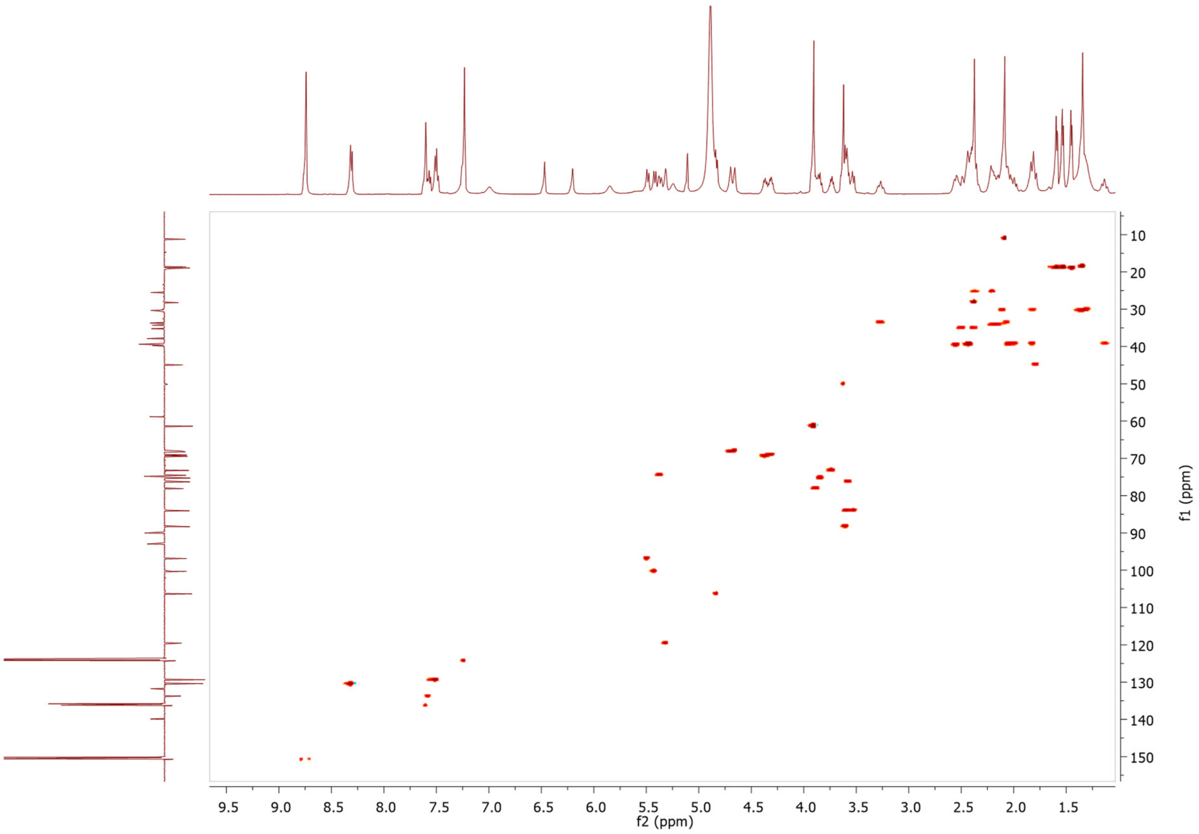

Figure S9. HSQC spectrum of compound **2** (in C_5D_5N).

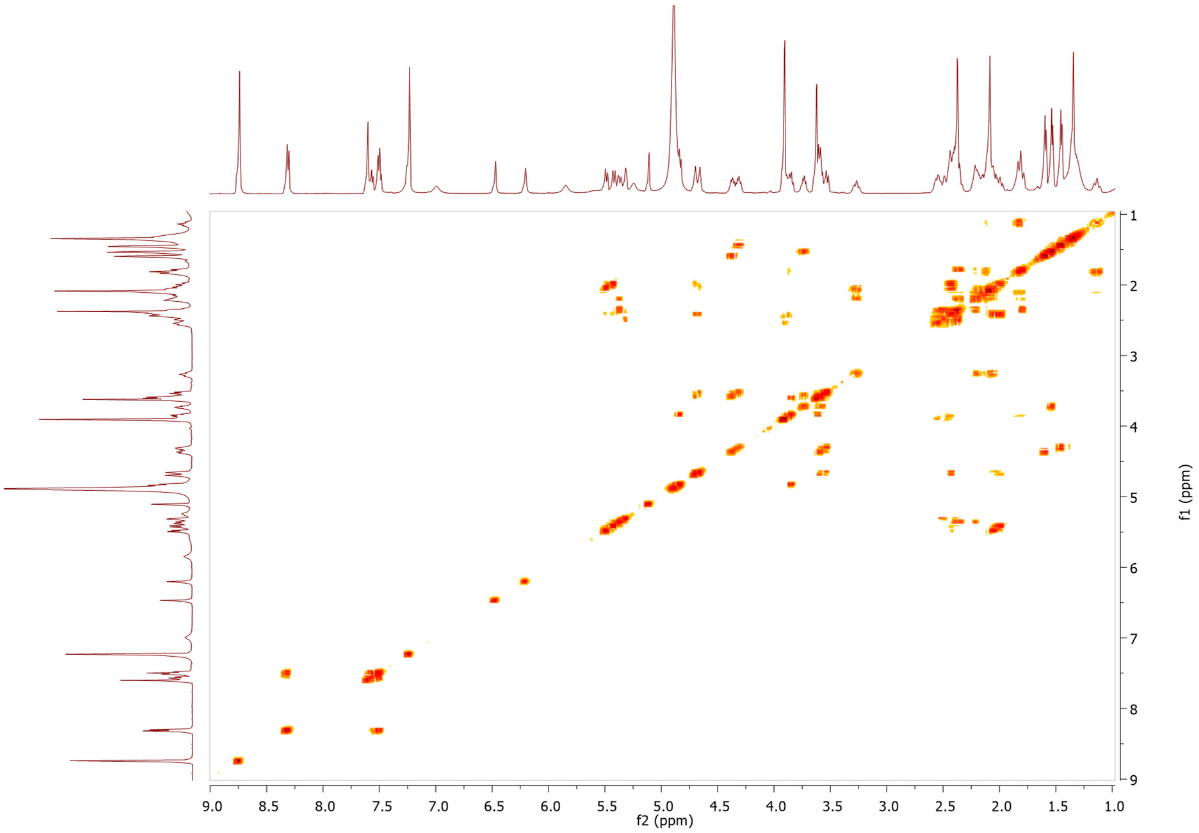

Figure S10. ^1H-^1H COSY spectrum of compound **2** (in C_5D_5N).

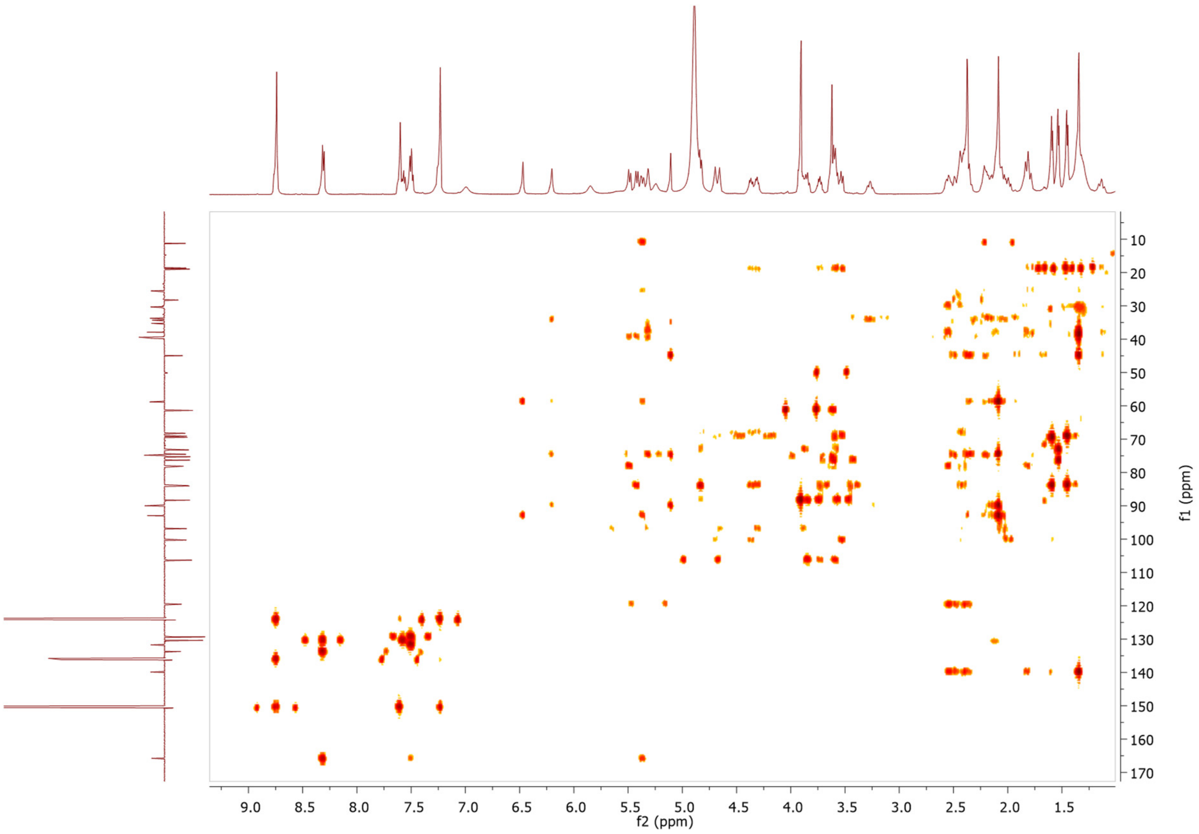

Figure S11. HMBC spectrum of compound **2** (in C_5D_5N).

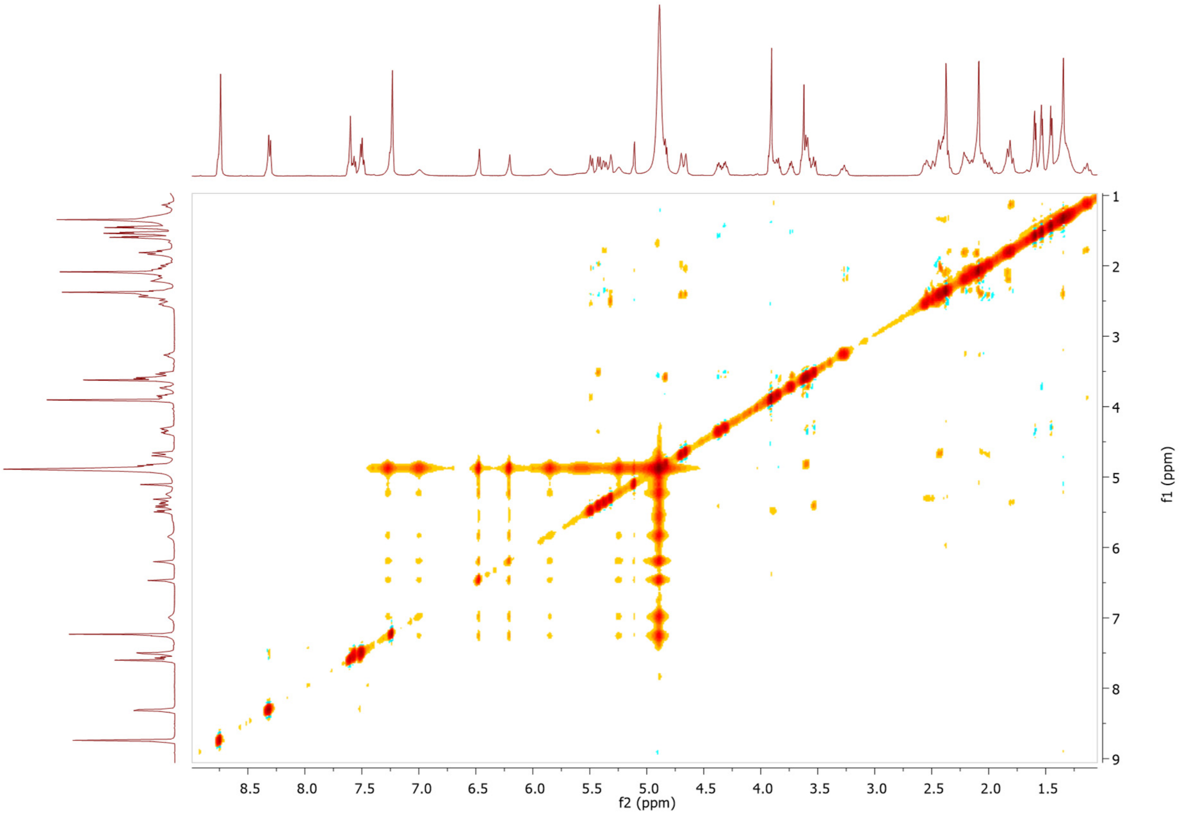

Figure S12. NOESY spectrum of compound **2** (in C_5D_5N).

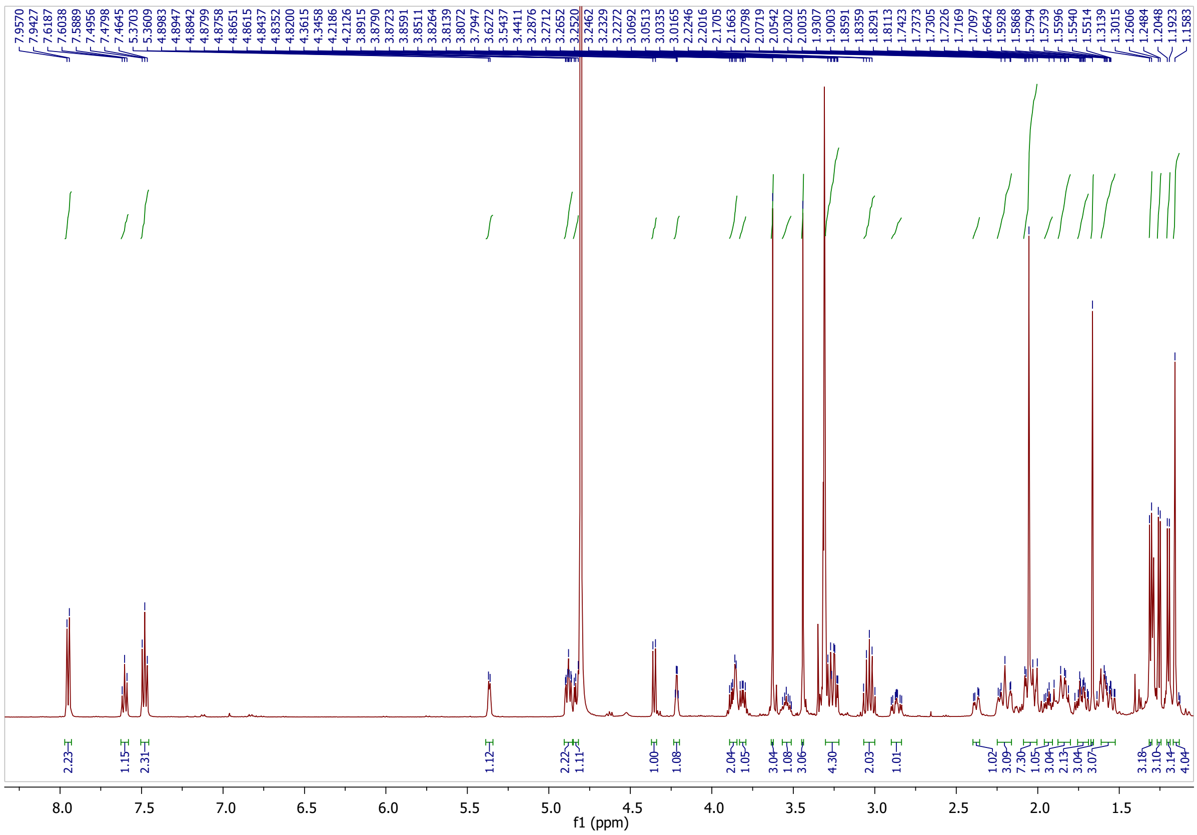

Figure S13. ^{1}H NMR spectrum of compound **3** (500 MHz, in CD_3OD).

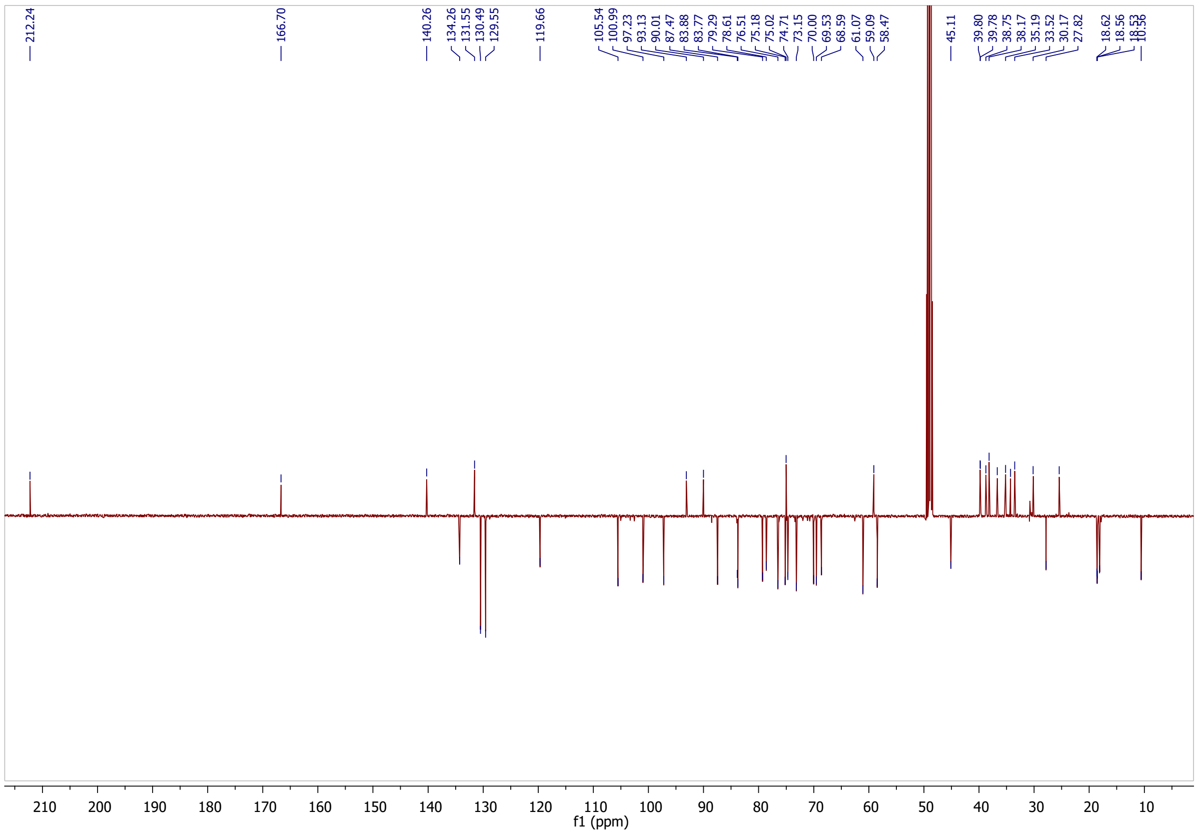

Figure S14. ^{13}C (JMOD) NMR spectrum of compound **3** (125 MHz, in CD_3OD).

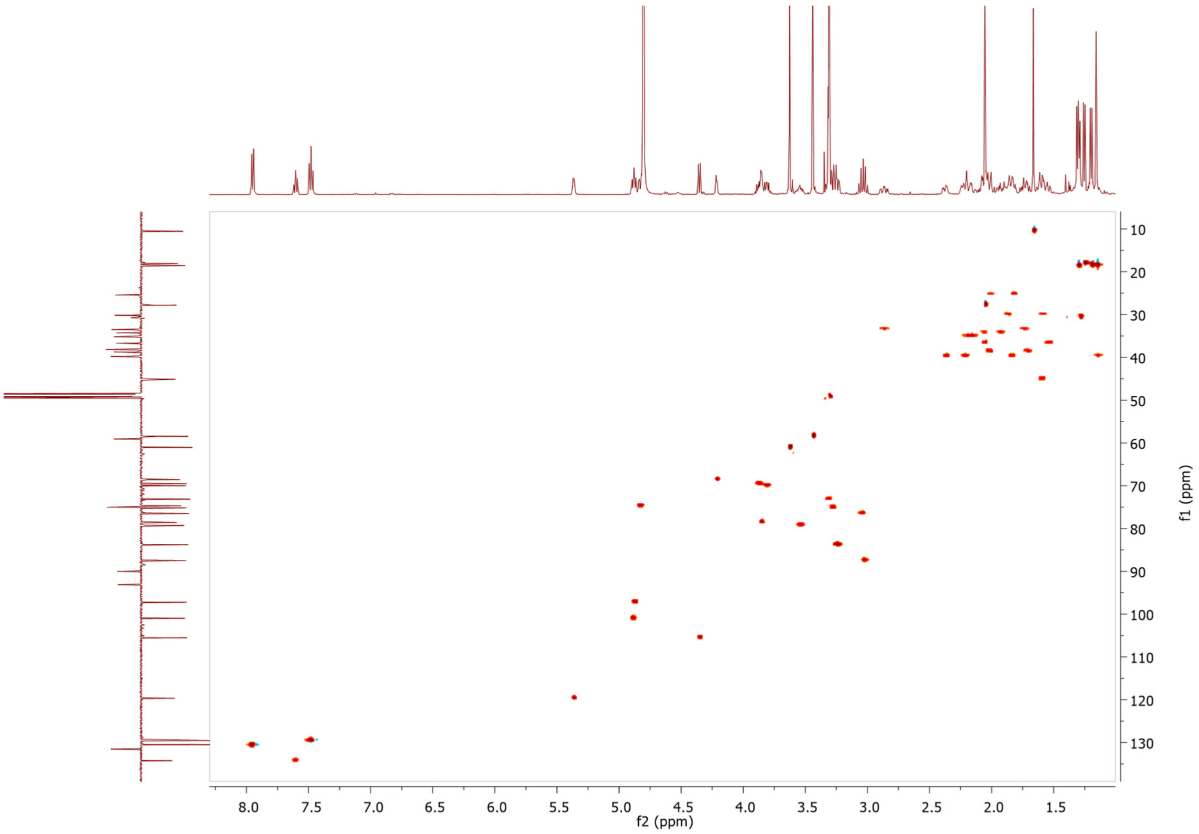

Figure S15. HSQC spectrum of compound **3** (in CD_3OD).

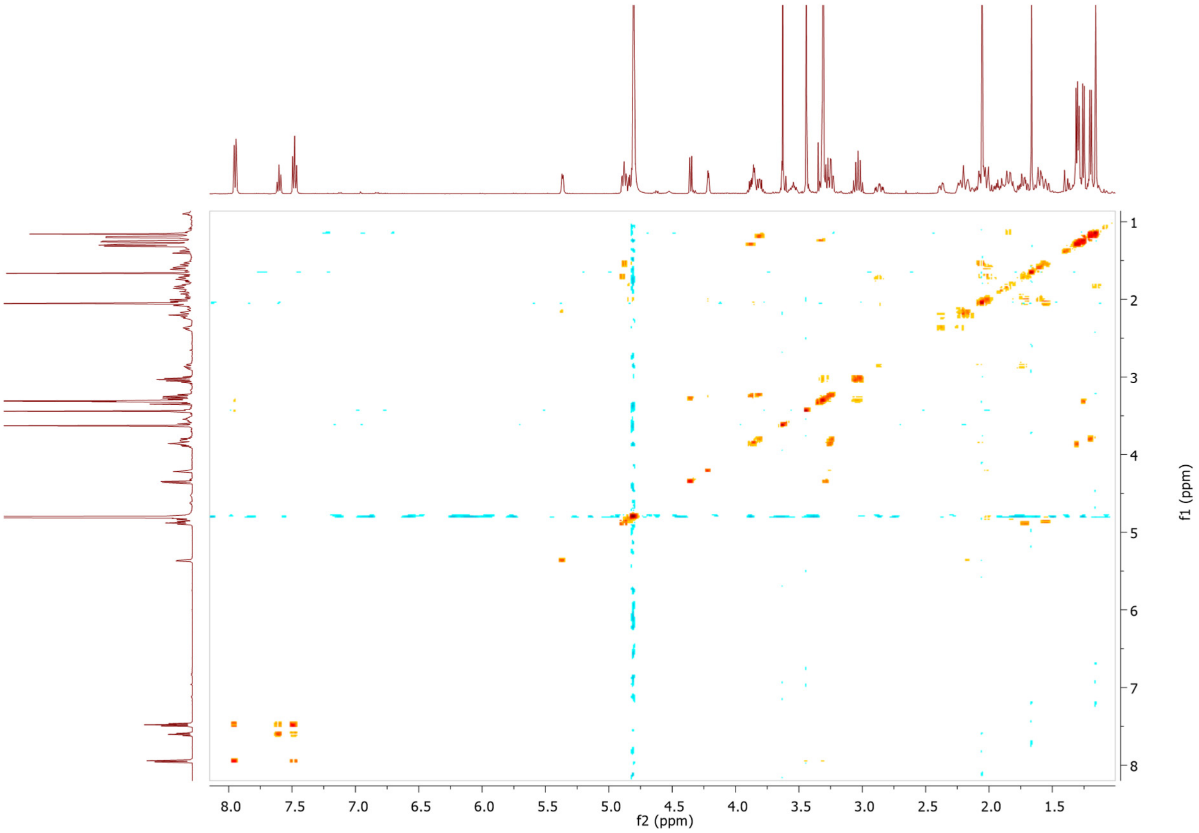

Figure S16. ^1H-^1H COSY spectrum of compound **3** (in CD_3OD).

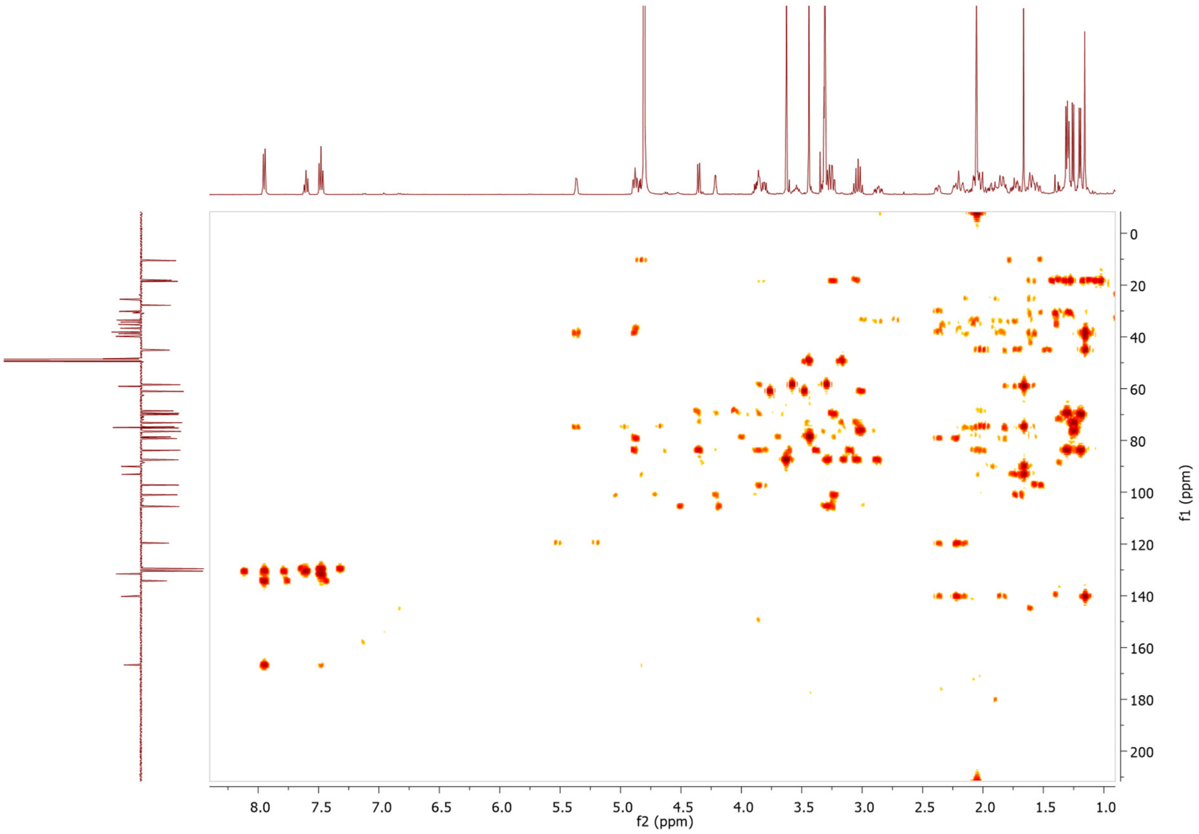

Figure S17. HMBC spectrum of compound **3** (in CD_3OD).

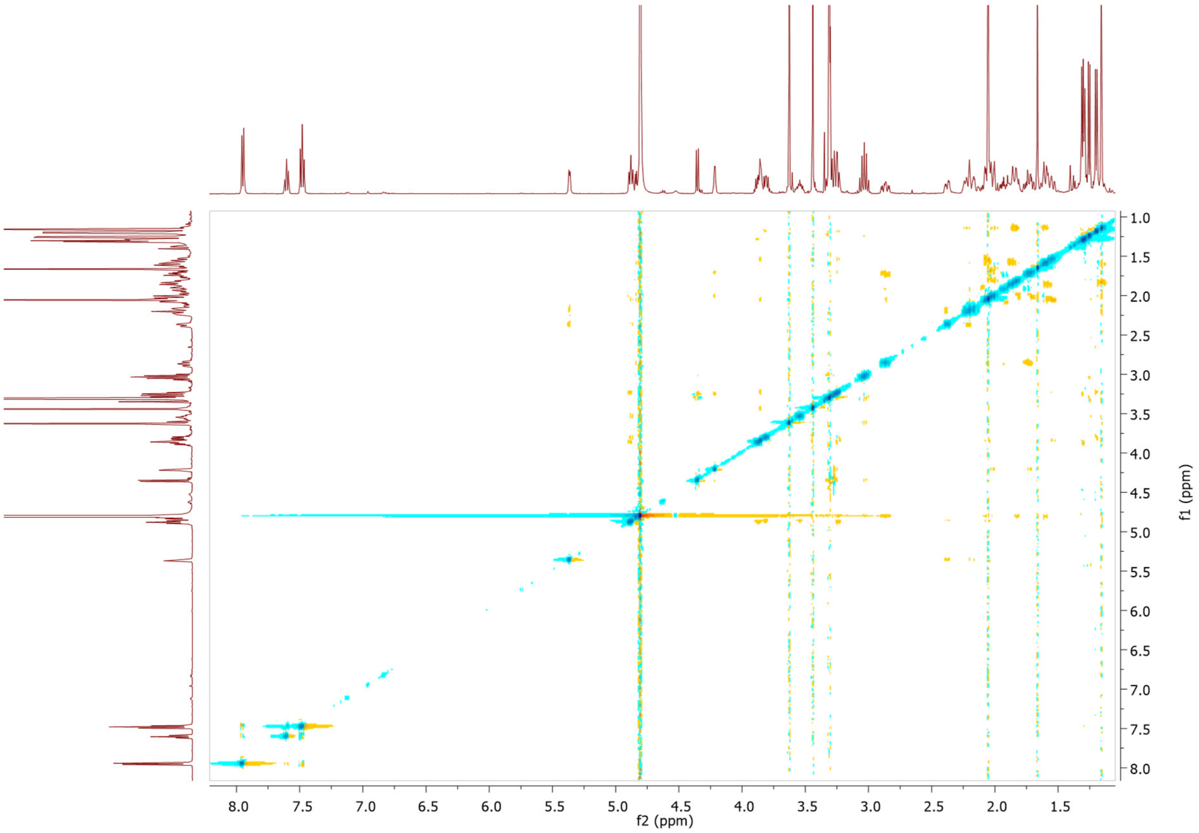

Figure S18. NOESY spectrum of compound **3** (in CD_3OD).

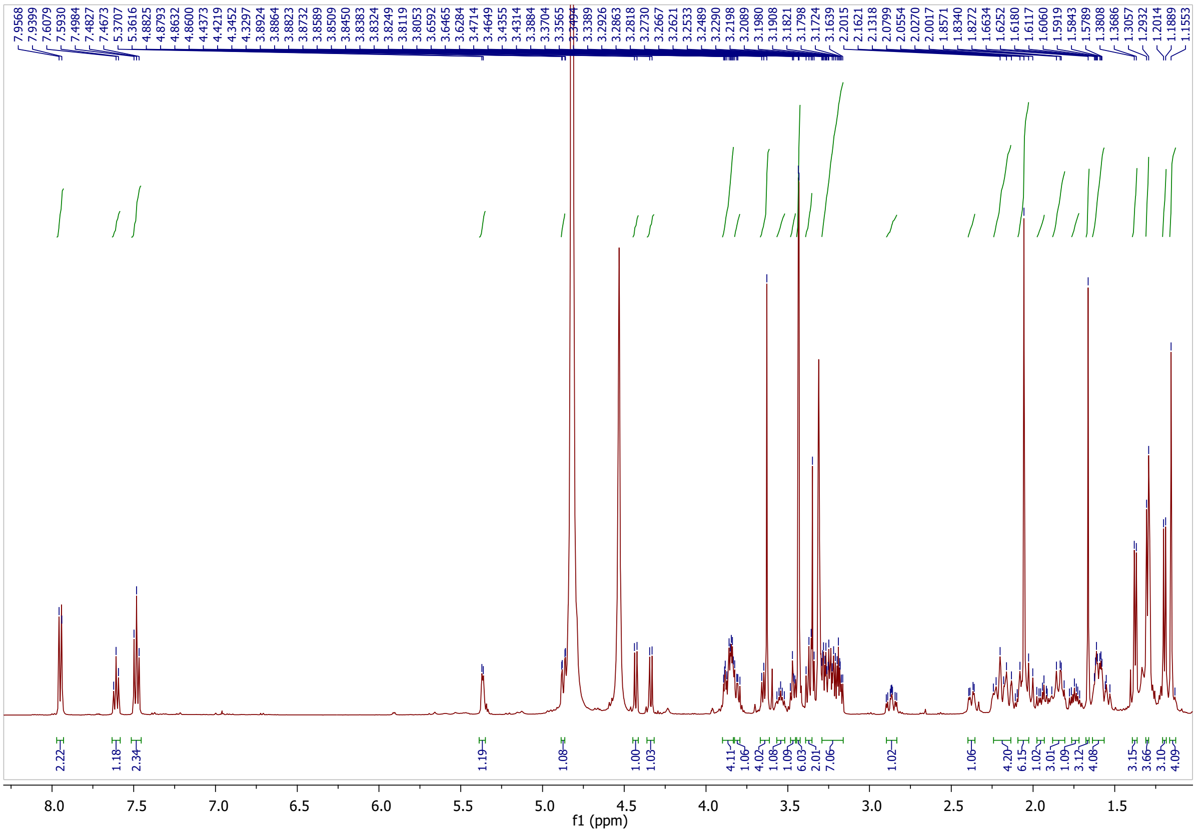

Figure S19. ^{1}H NMR spectrum of compound **4** (500 MHz, in CD_3OD).

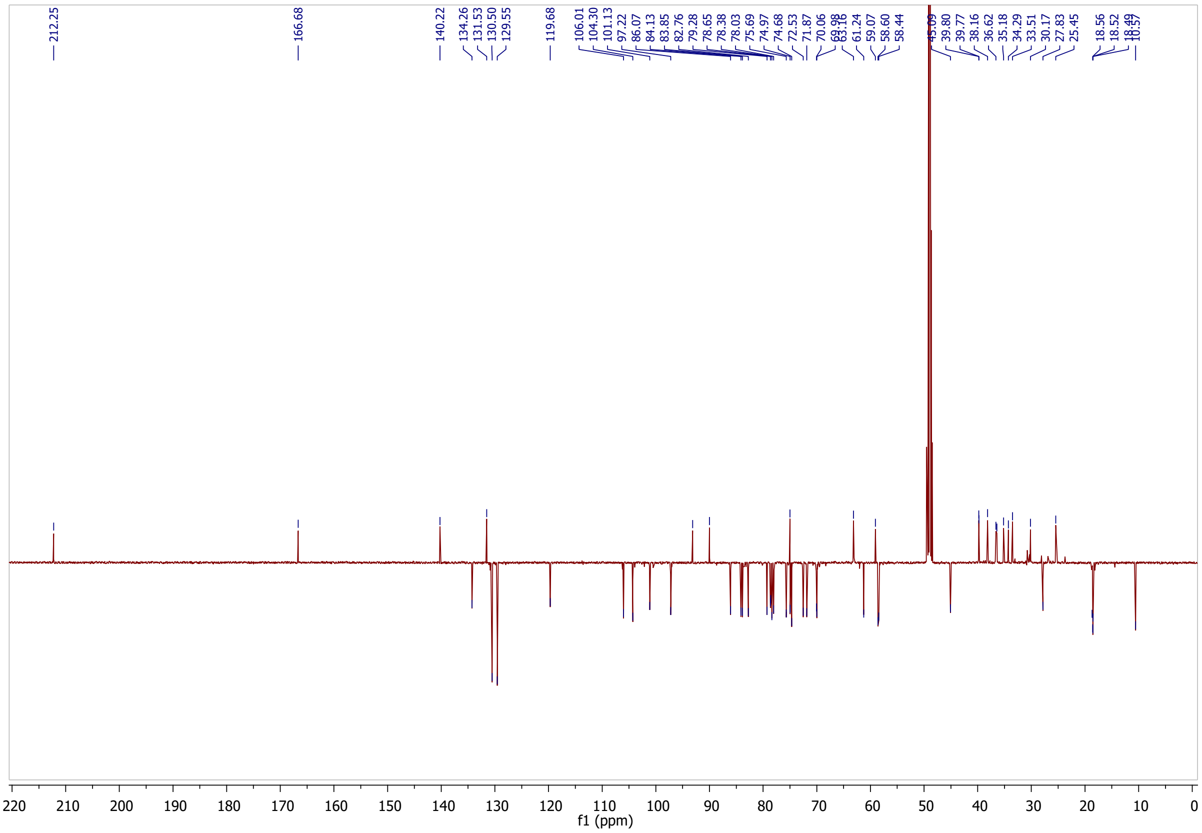

Figure S20. ^{13}C (JMOD) NMR spectrum of compound **4** (125 MHz, in CD_3OD).

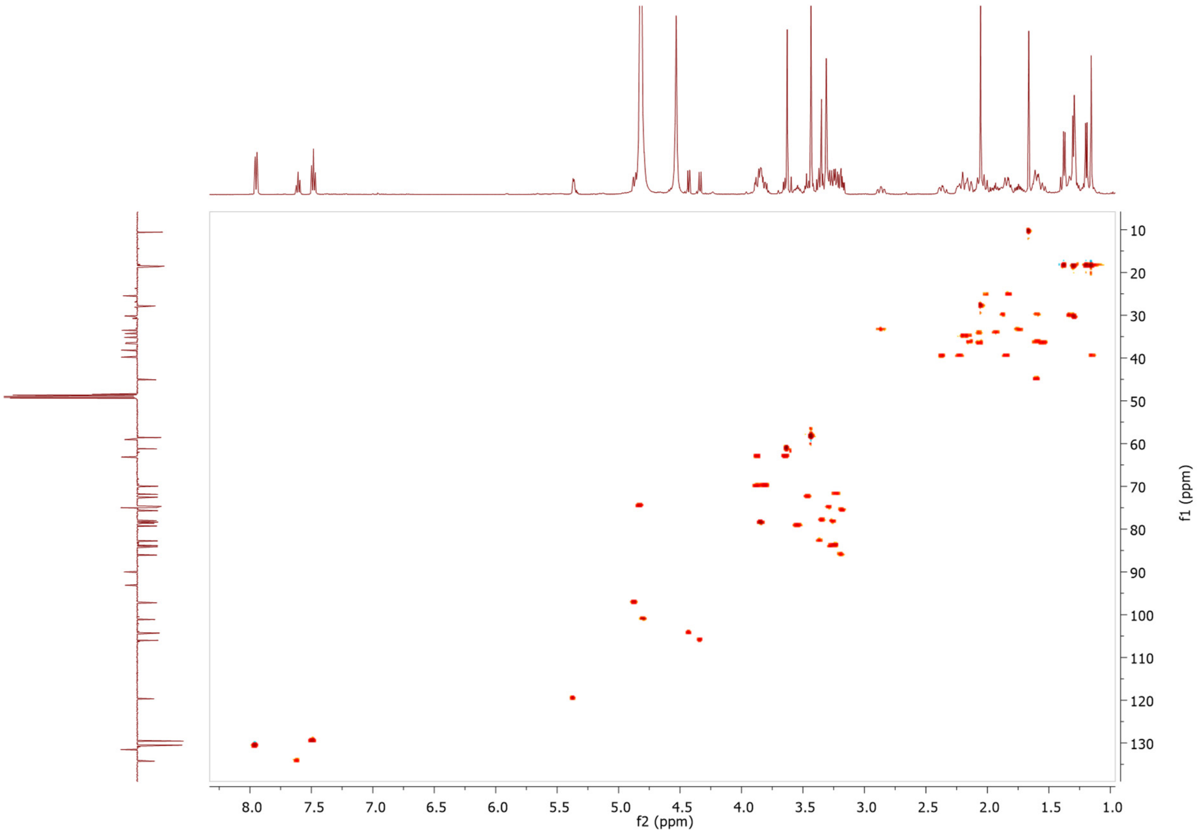

Figure S21. HSQC spectrum of compound **4** (in CD_3OD).

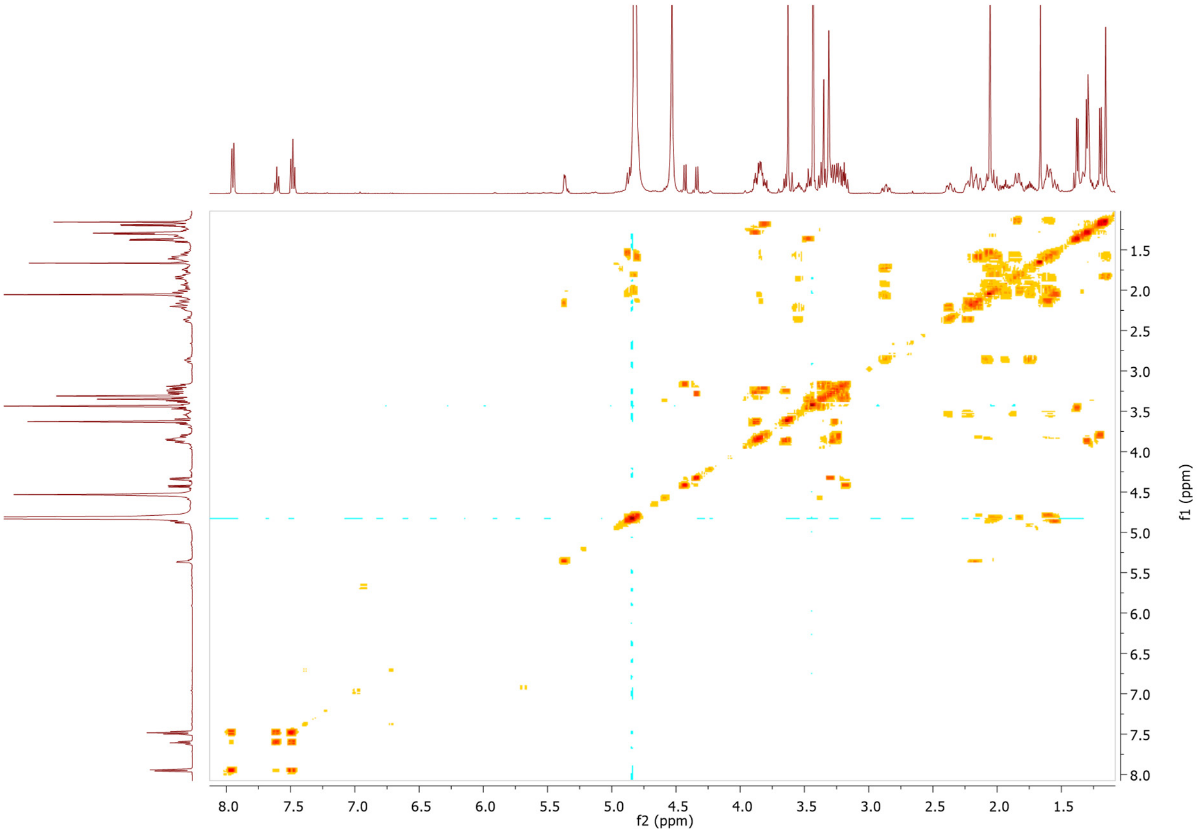

Figure S22. ^1H-^1H COSY spectrum of compound **4** (in CD_3OD).

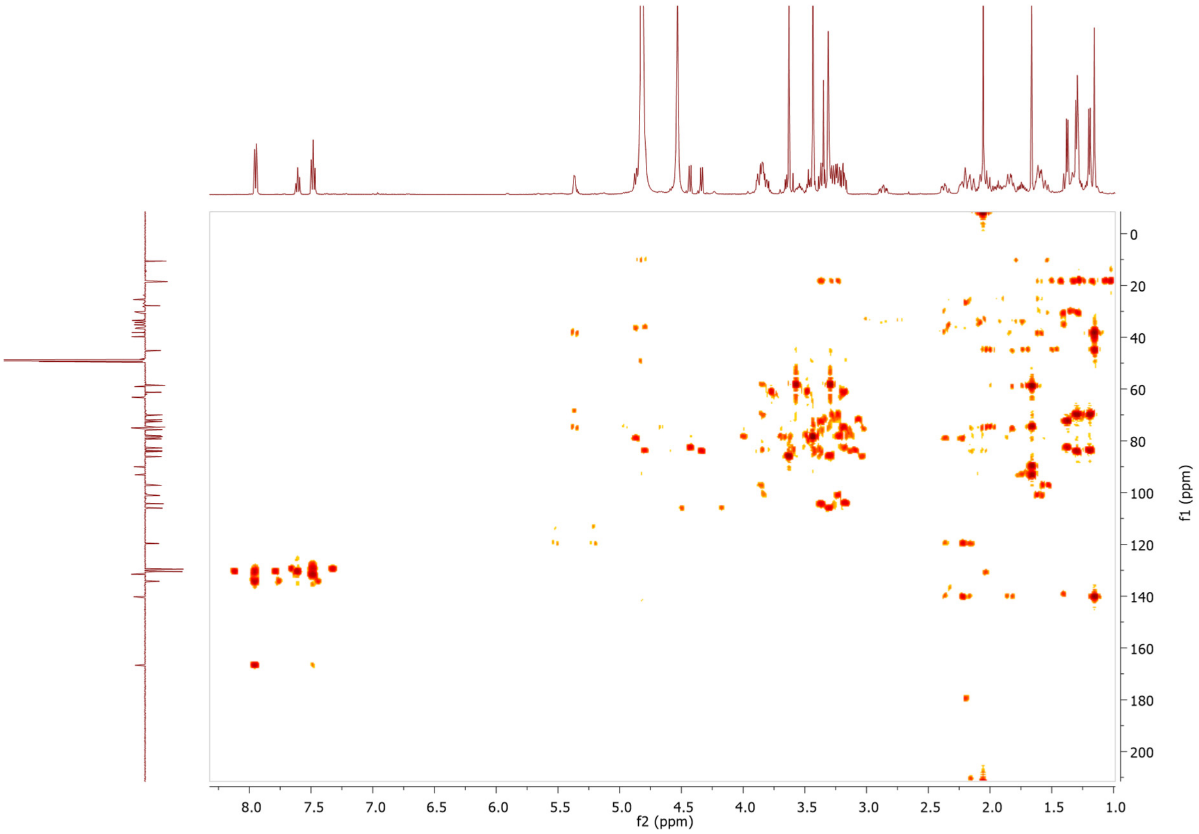

Figure S23. HMBC spectrum of compound **4** (in CD_3OD).

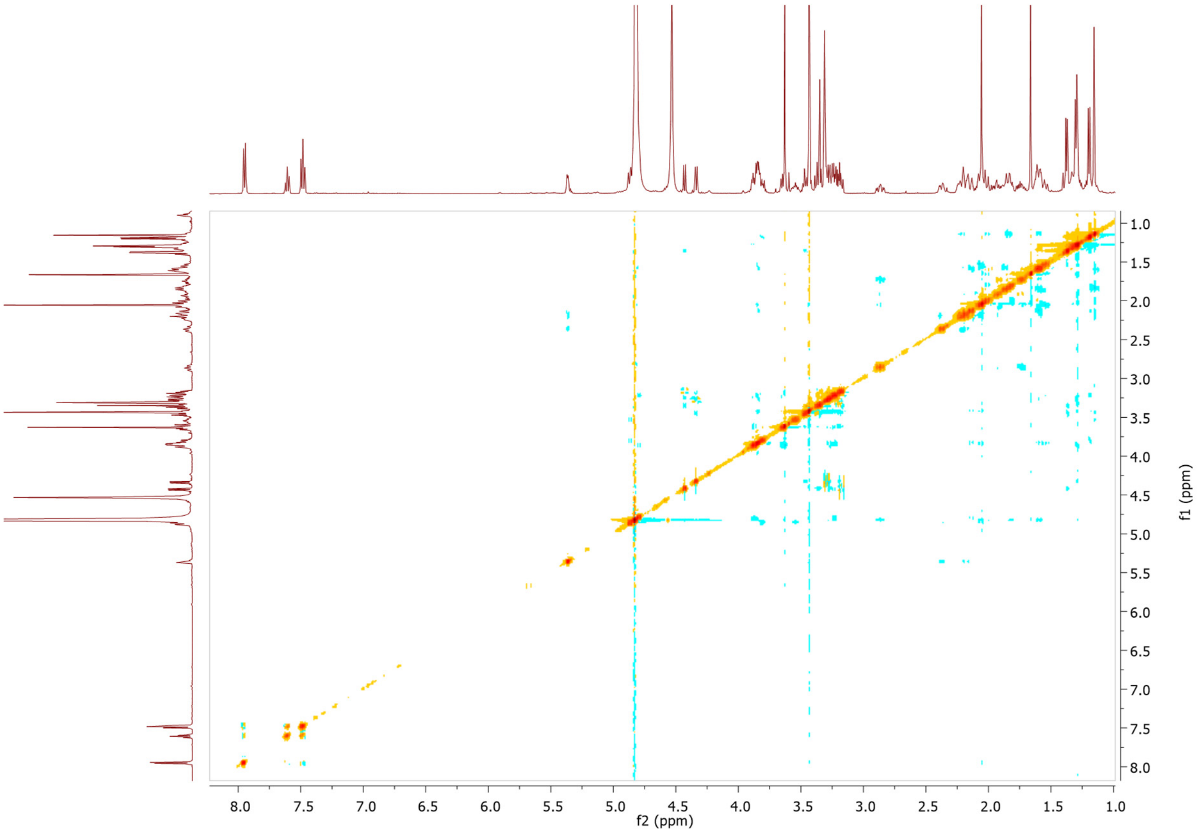

Figure S24. NOESY spectrum of compound **4** (in CD_3OD).

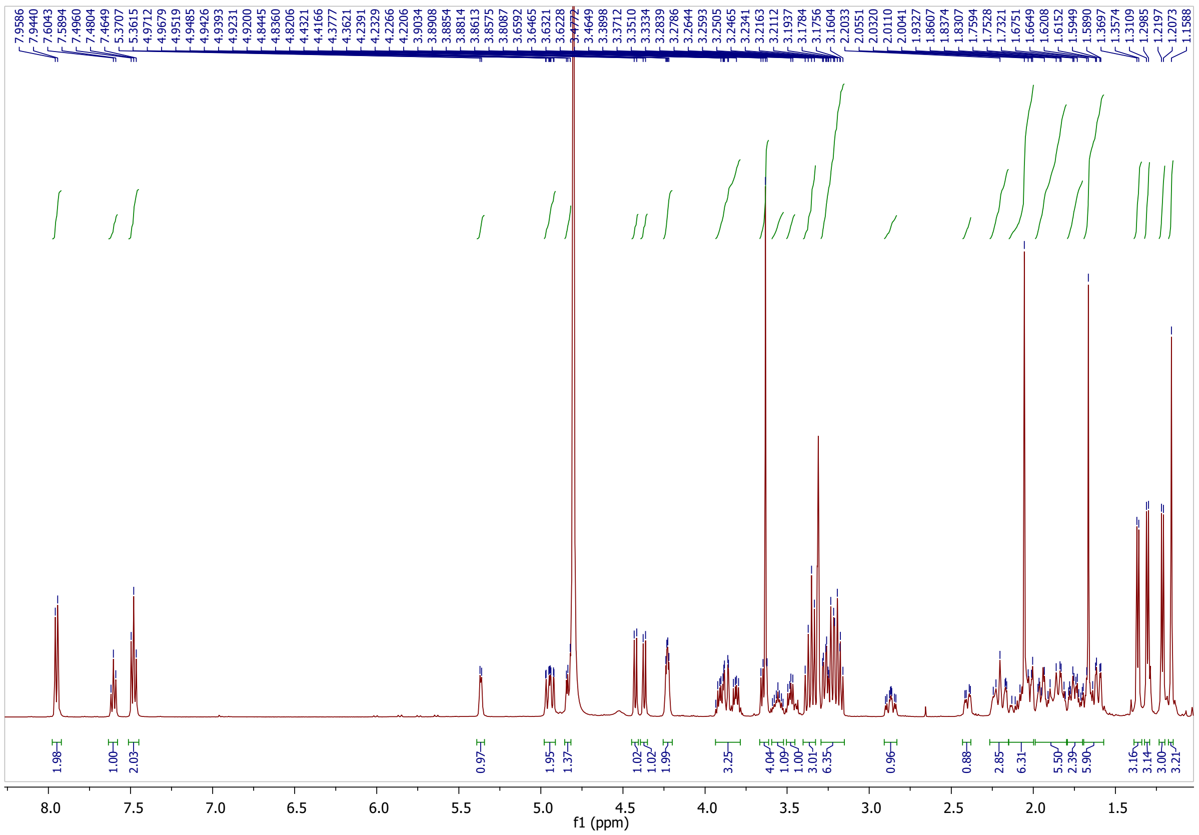

Figure S25. ^{1}H NMR spectrum of compound **5** (500 MHz, in CD_3OD).

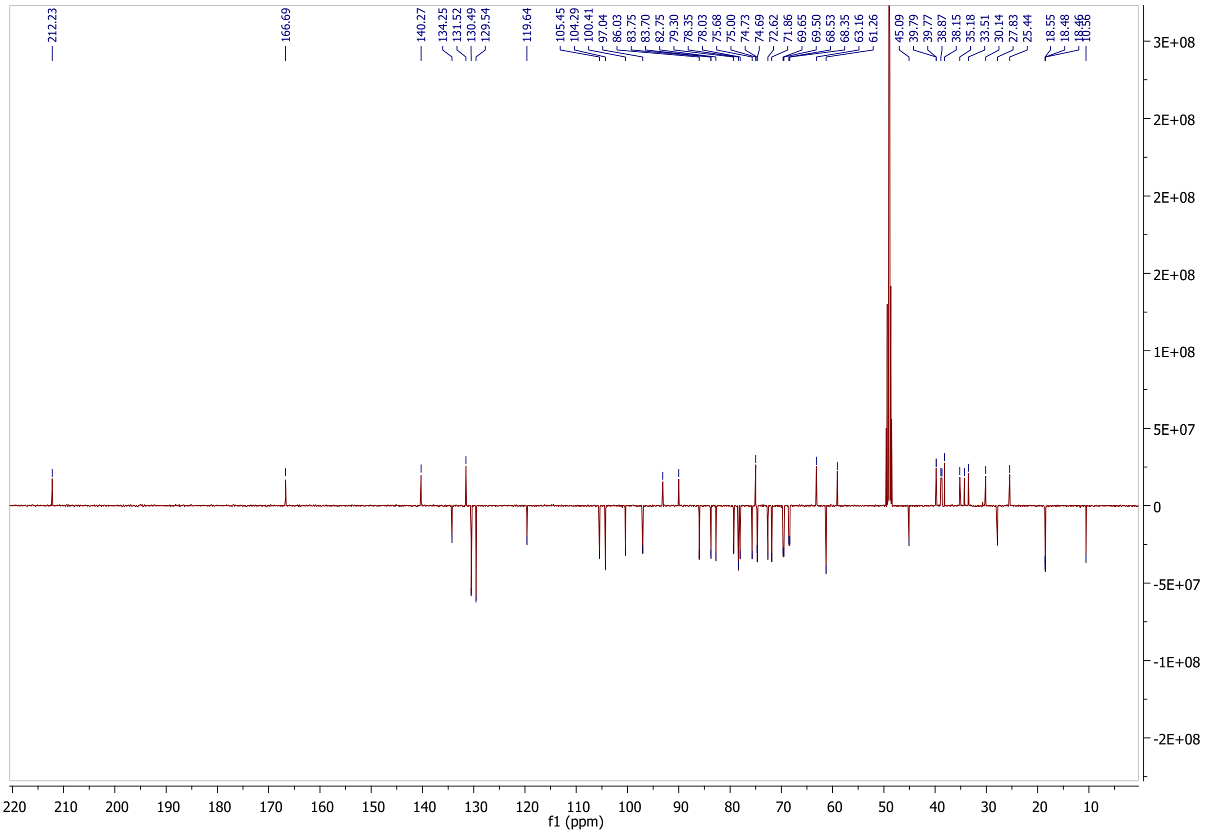

Figure S25. ^{13}C (JMOD) NMR spectrum of compound **5** (125 MHz, in CD_3OD).

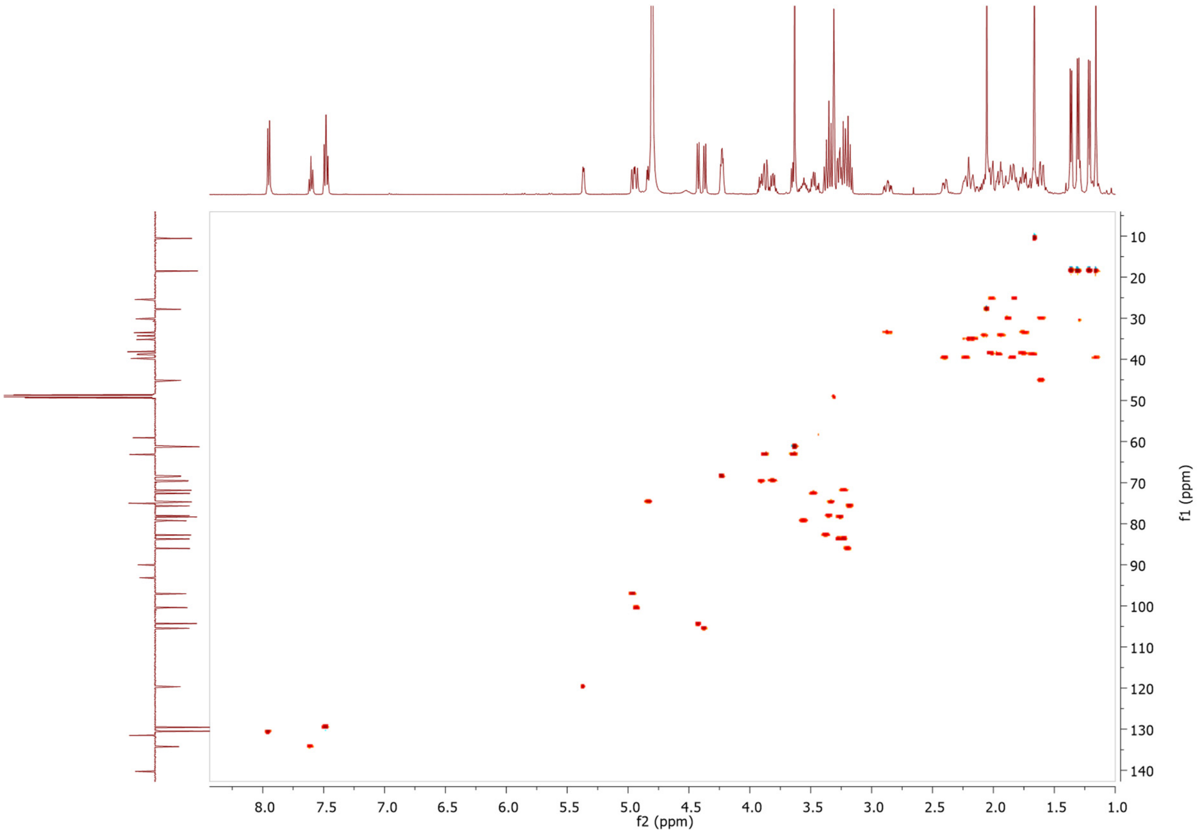

Figure S26. HSQC spectrum of compound **5** (in CD_3OD).

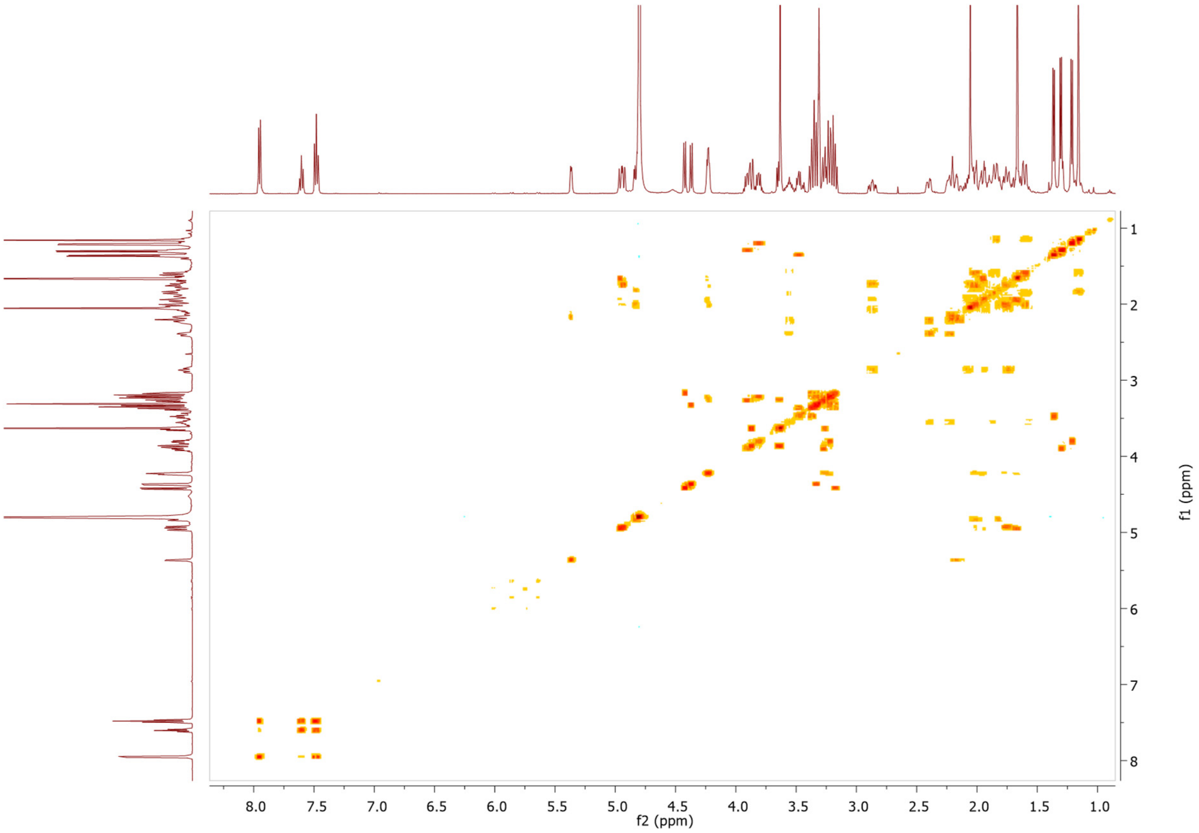

Figure S27. ^1H-^1H COSY spectrum of compound **5** (in CD_3OD).

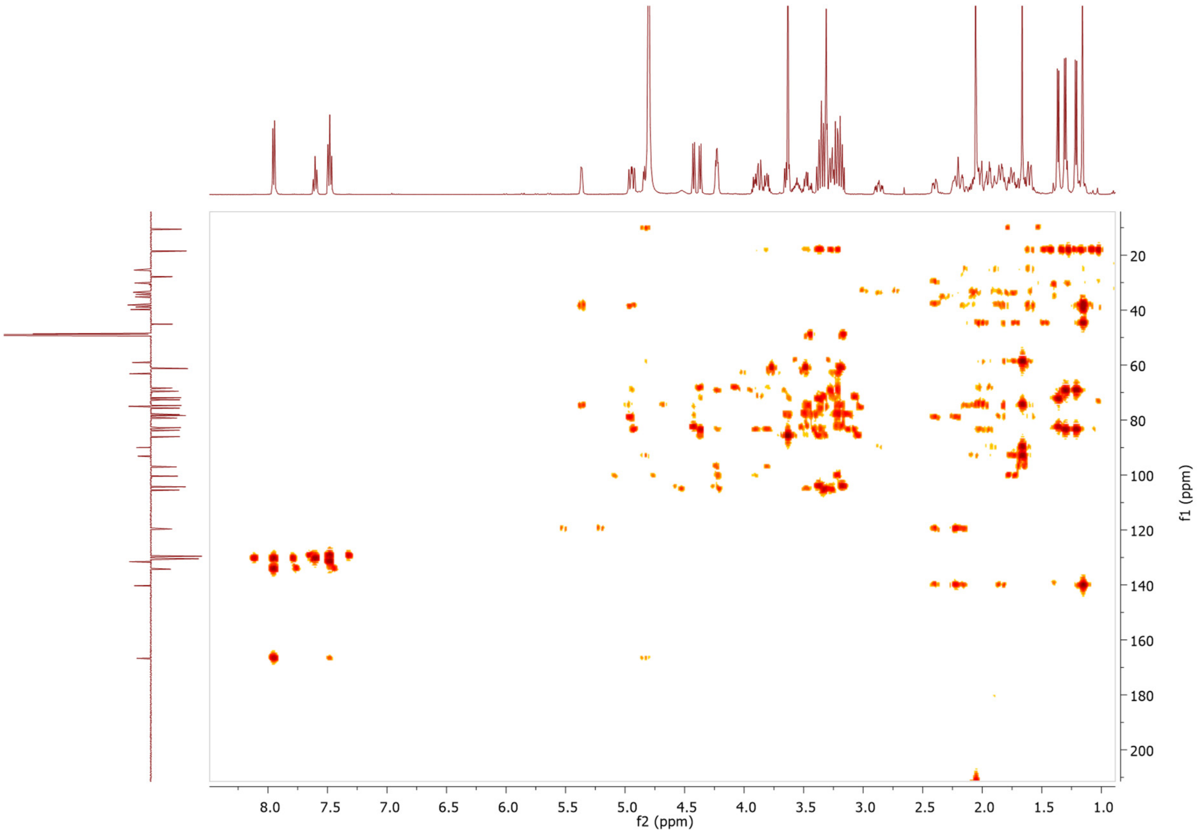

Figure S28. HMBC spectrum of compound **5** (in CD_3OD).

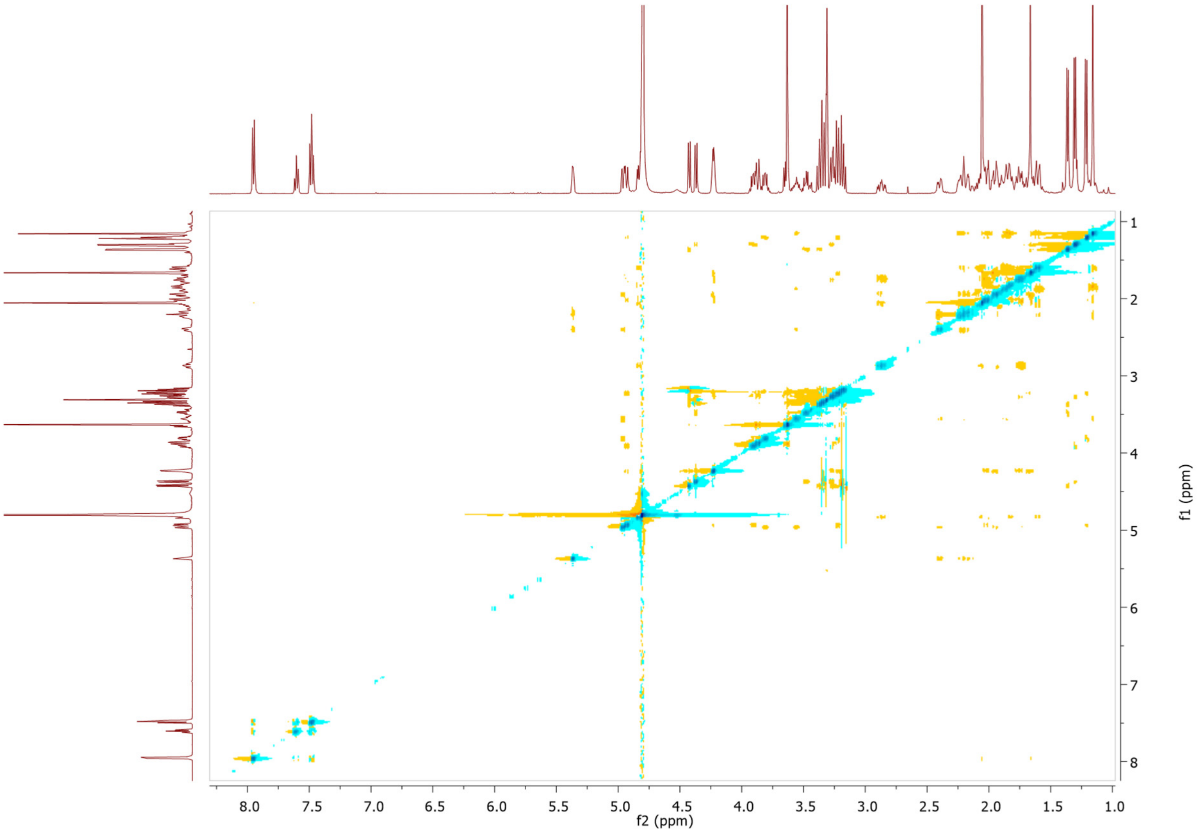

Figure S29. NOESY spectrum of compound **5** (in CD_3OD).

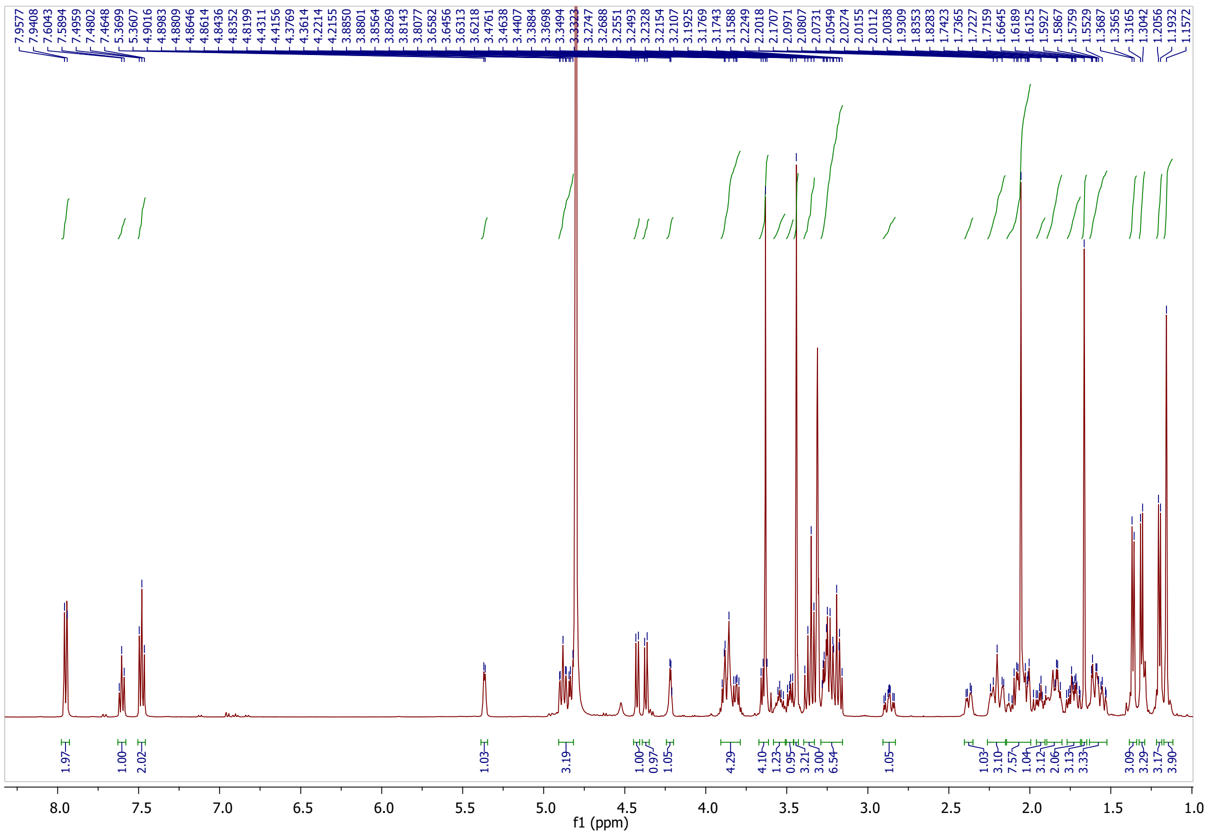

Figure S30. ^{1}H NMR spectrum of compound **6** (500 MHz, in CD_3OD).

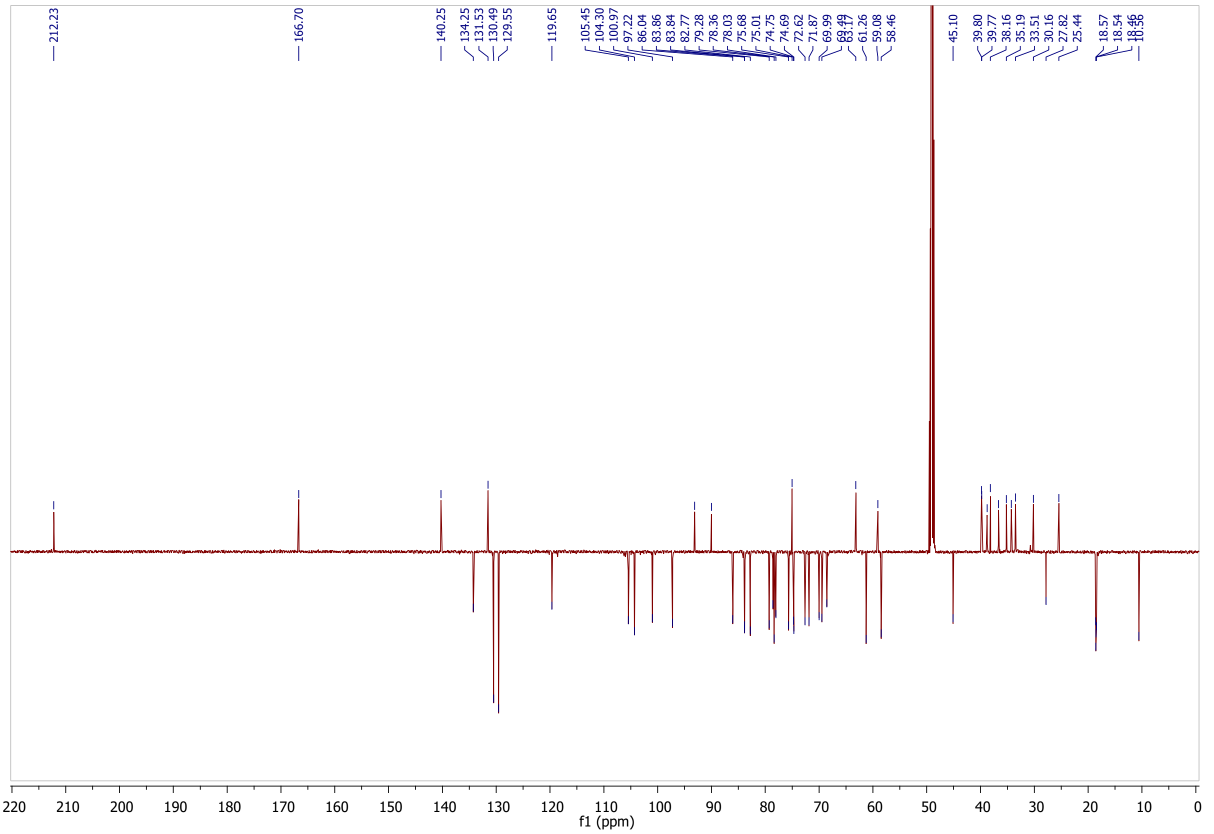

Figure S31. ^{13}C (JMOD) NMR spectrum of compound **6** (125 MHz, in CD_3OD).

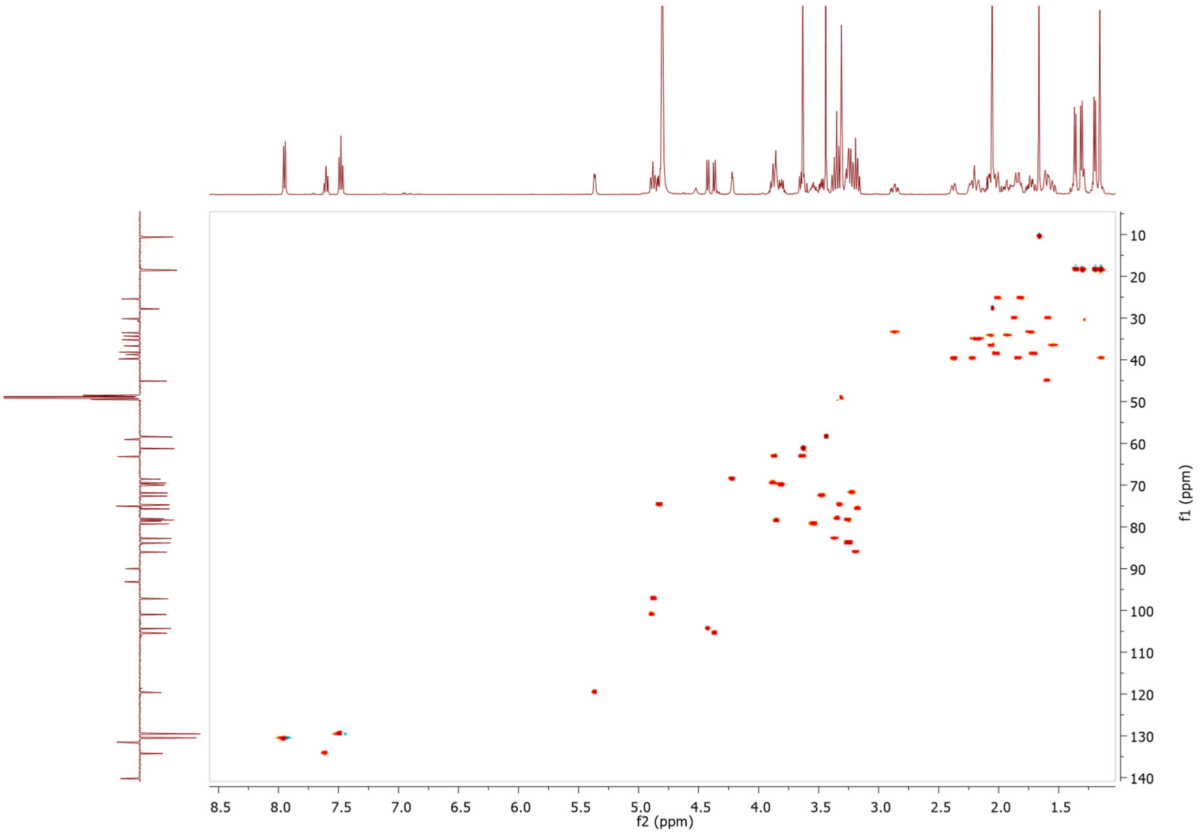

Figure S32. HSQC spectrum of compound **6** (in CD_3OD).

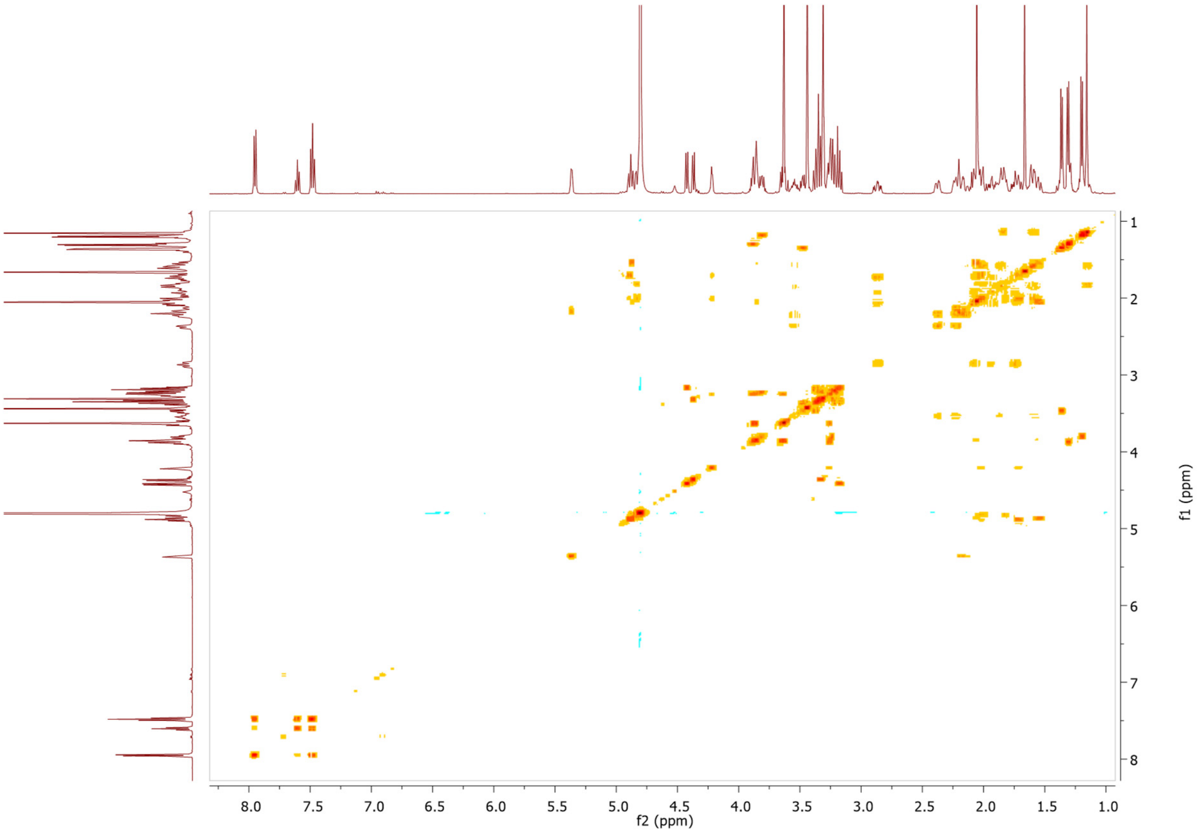

Figure S33. ^{1}H-^{1}H COSY spectrum of compound **6** (in CD_3OD).

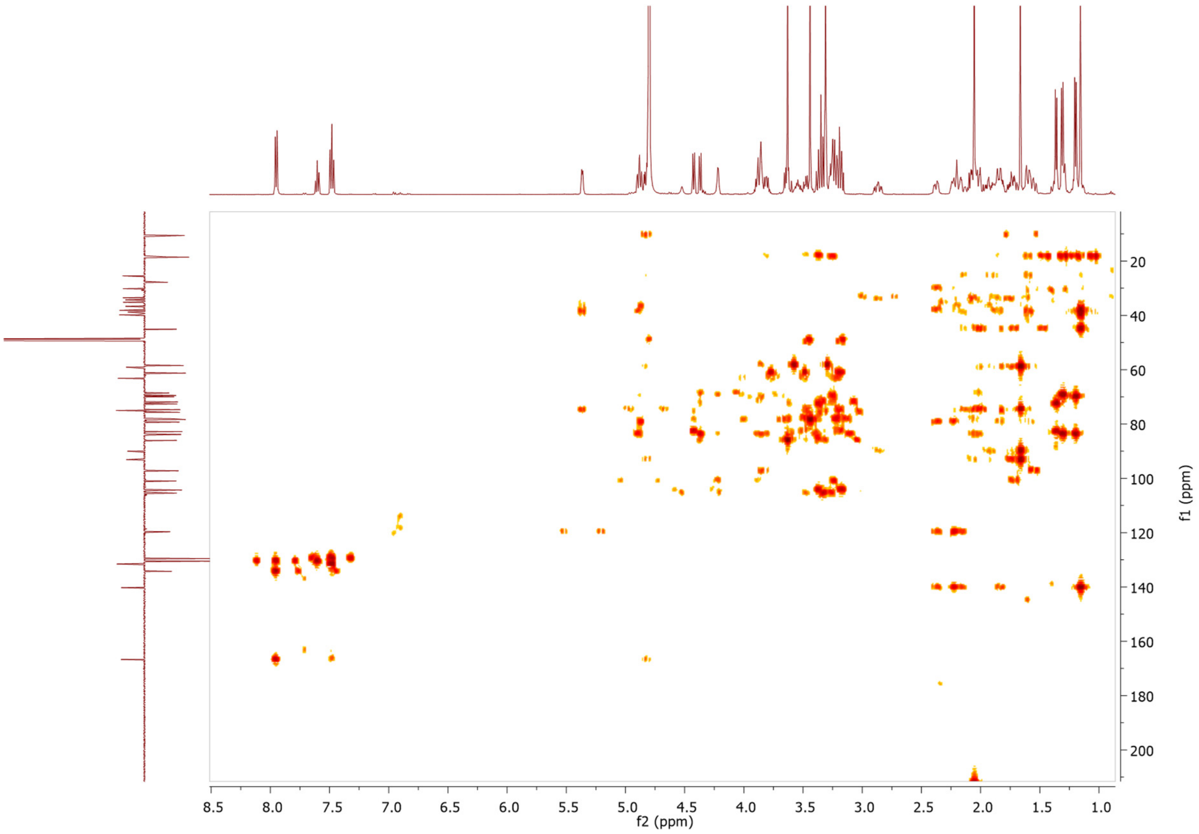

Figure S34. HMBC spectrum of compound **6** (in CD_3OD).

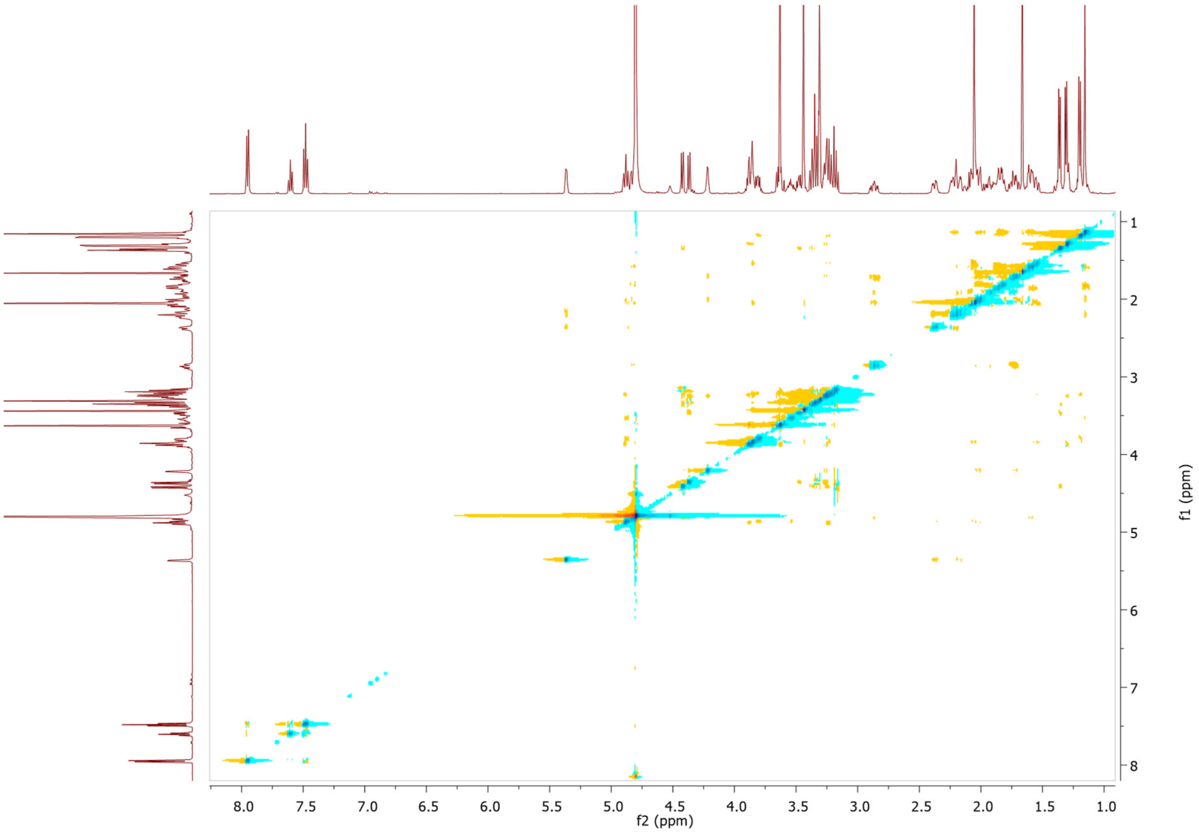

Figure S35. NOESY spectrum of compound **6** (in CD_3OD).

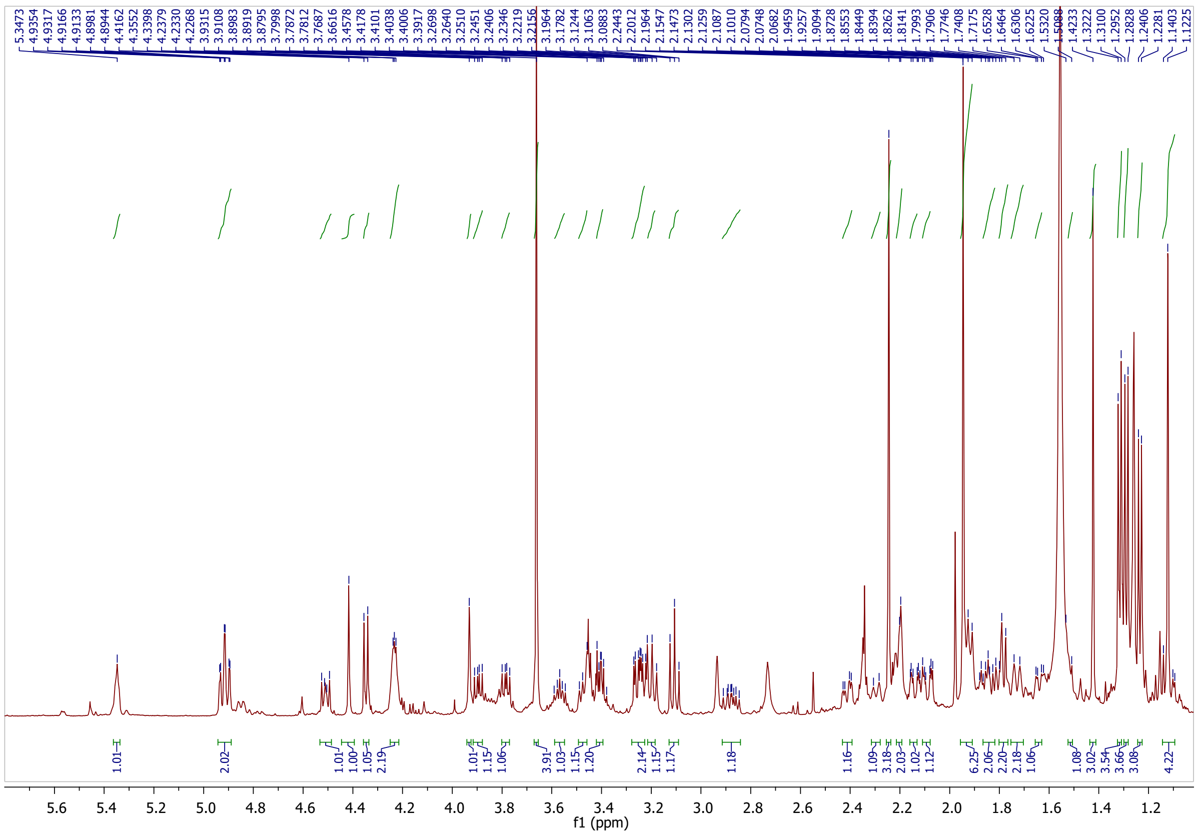

Figure S36. ^{1}H NMR spectrum of compound **7** (500 MHz, in $CDCl_3$).

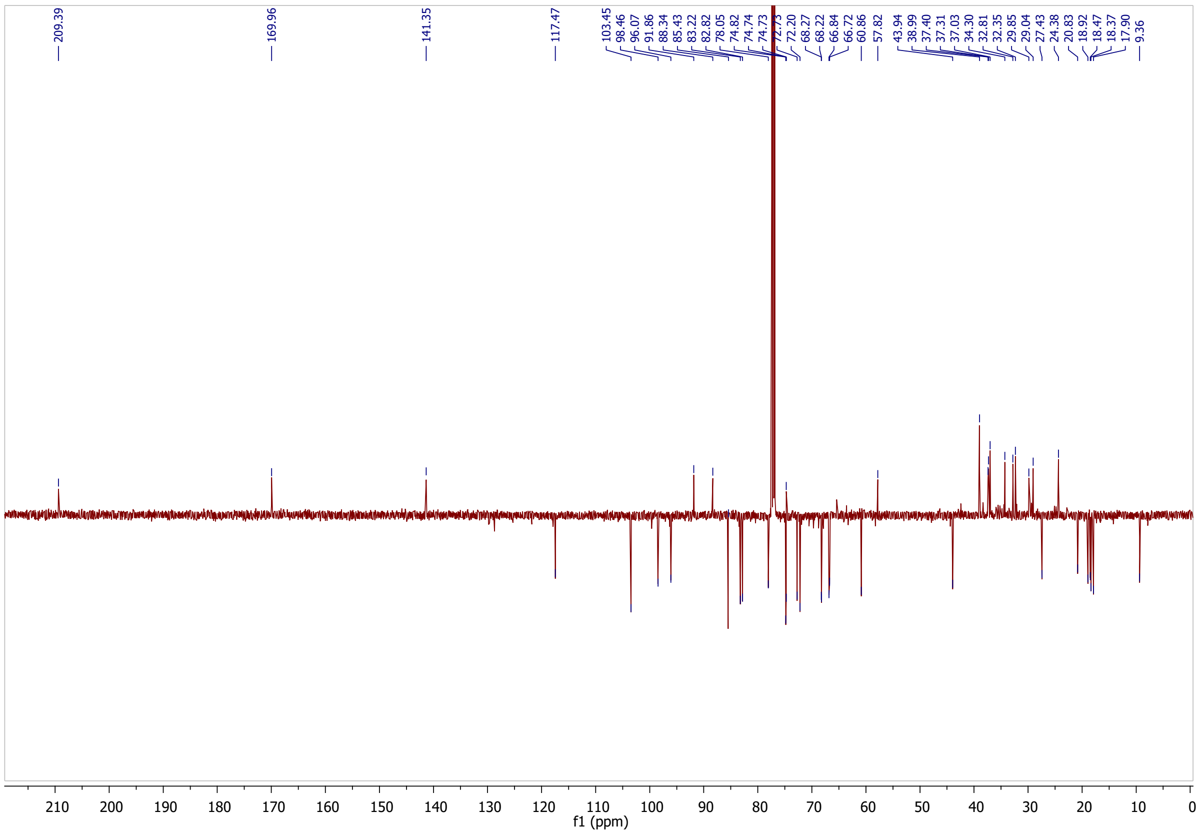

Figure S37. ^{13}C (JMOD) spectrum of compound **7** (125 MHz, in $CDCl_3$).

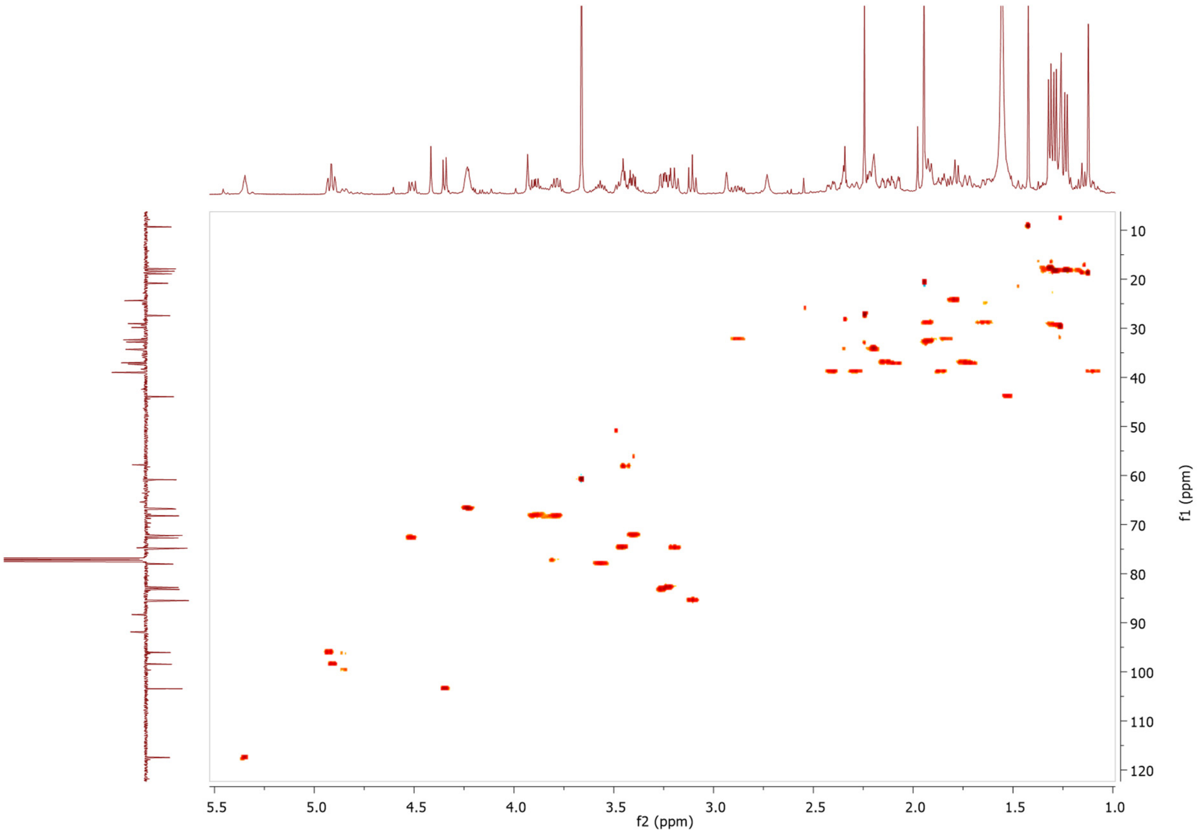

Figure S38. HSQC spectrum of compound **7** (in $CDCl_3$).

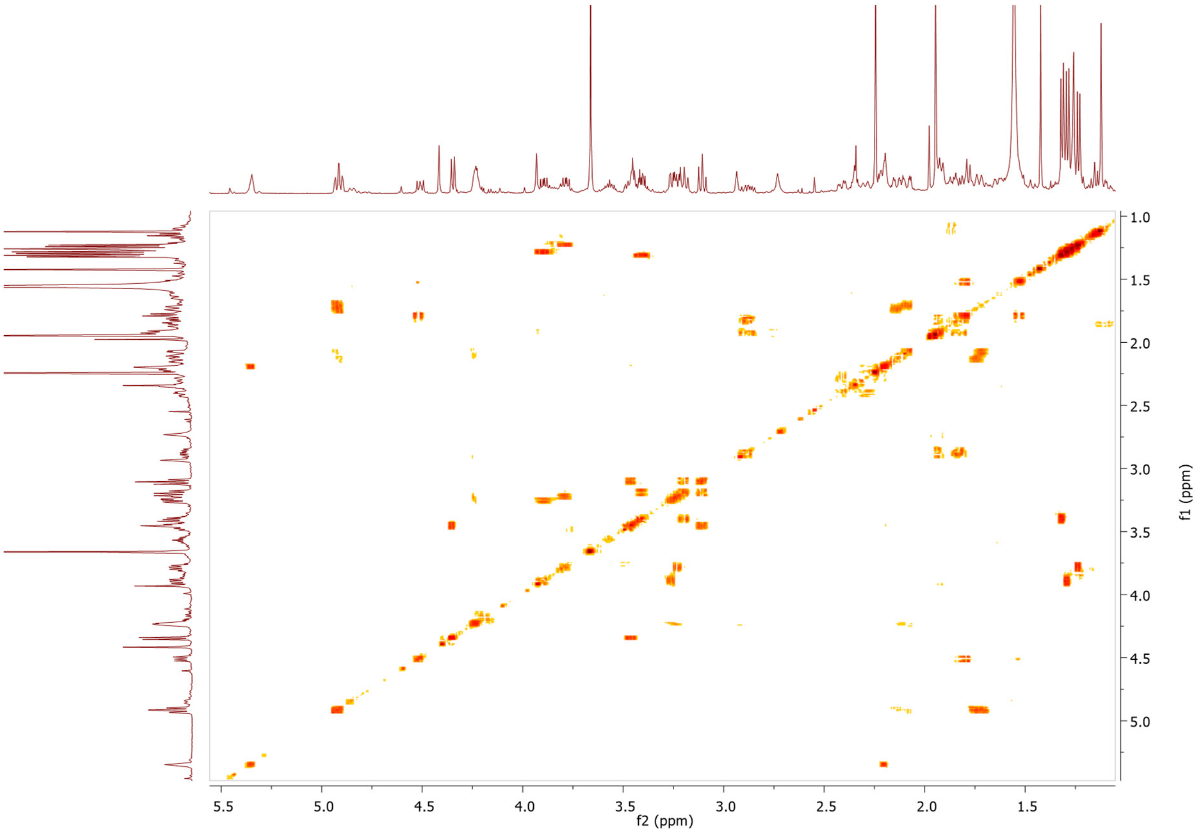

Figure S39. ^1H-^1H COSY spectrum of compound **7** (in $CDCl_3$).

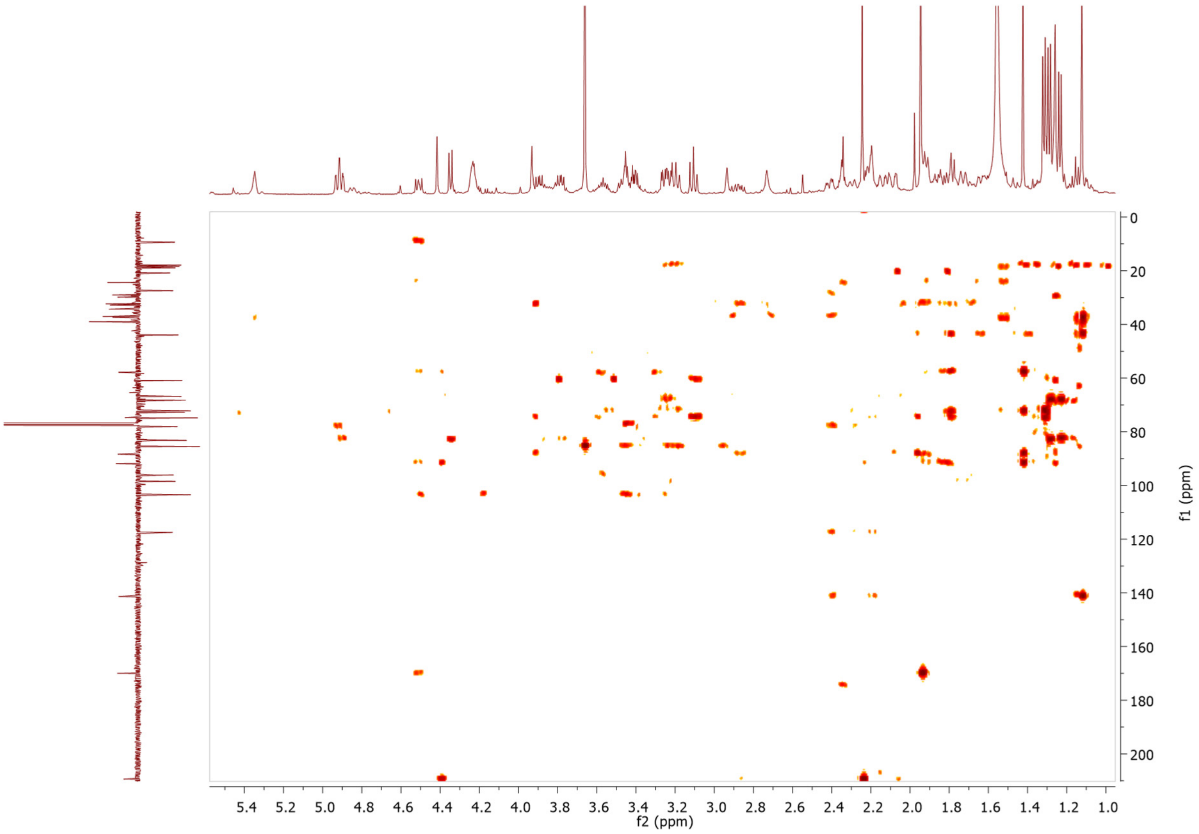

Figure S40. HMBC spectrum of compound **7** (in $CDCl_3$).

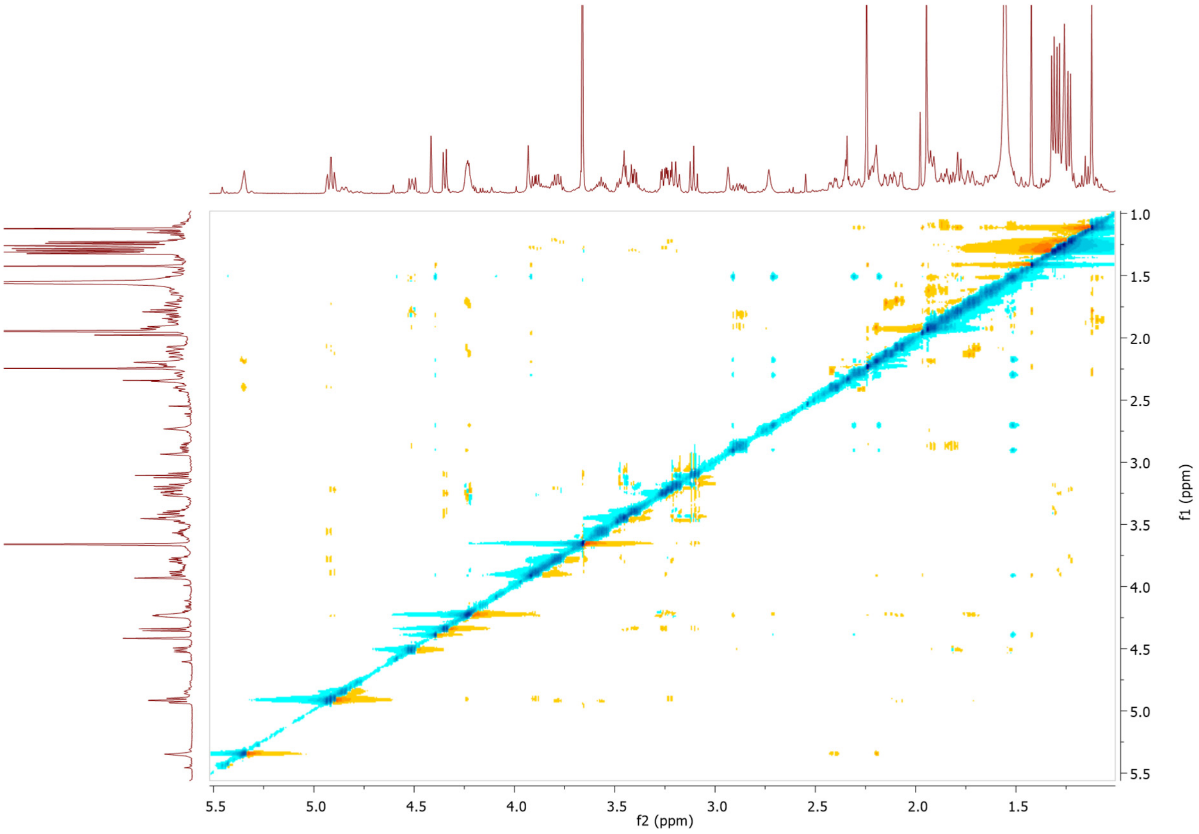

Figure S41. NOESY spectrum of compound **7** (in $CDCl_3$).

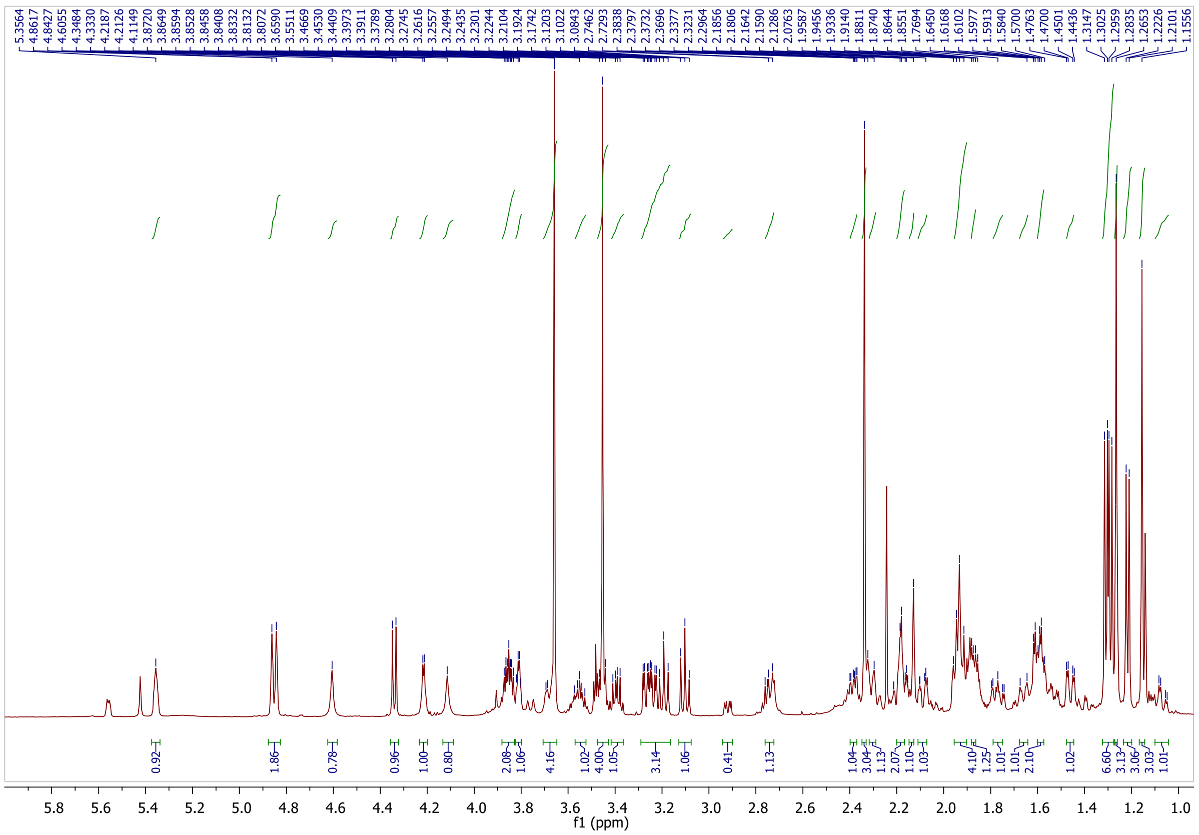

Figure S42. ^{1}H NMR spectrum of compound **8** (500 MHz, in $CDCl_3$).

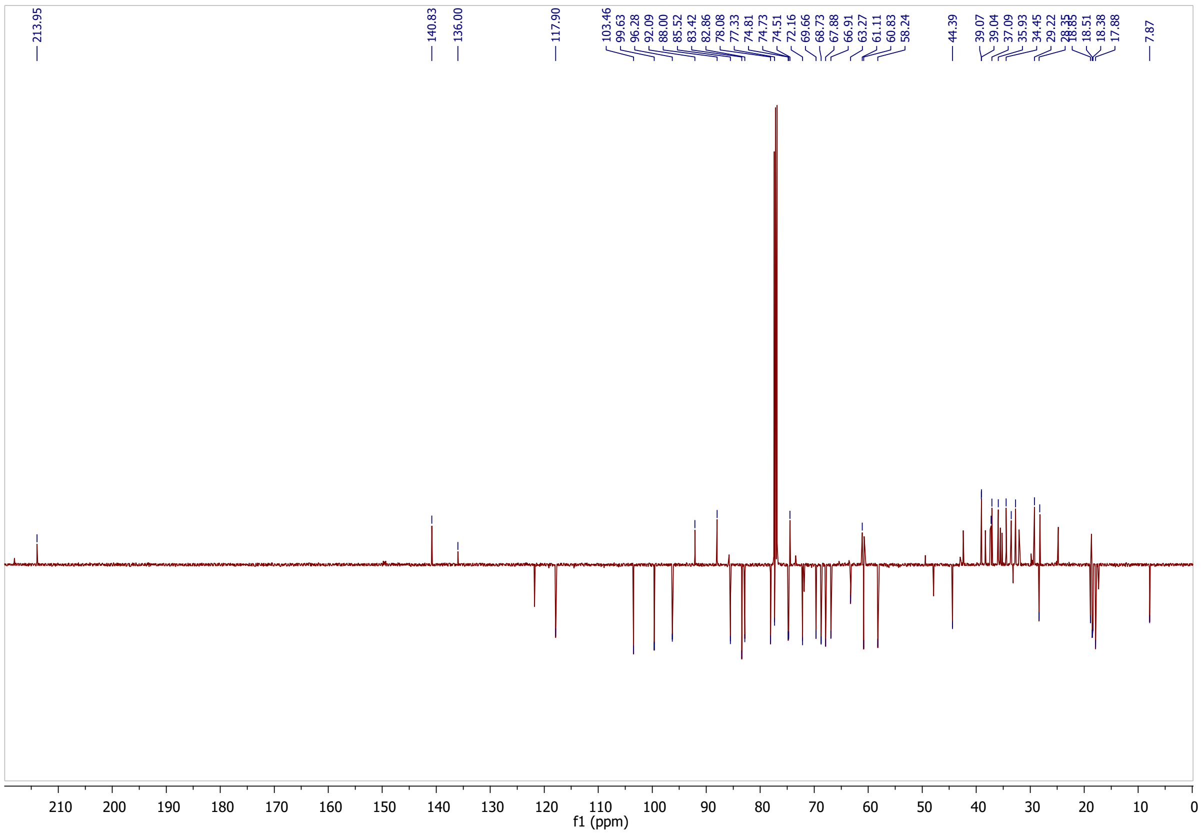

Figure S43. ^{13}C (JMOD) NMR spectrum of compound **8** (125 MHz, in $CDCl_3$).

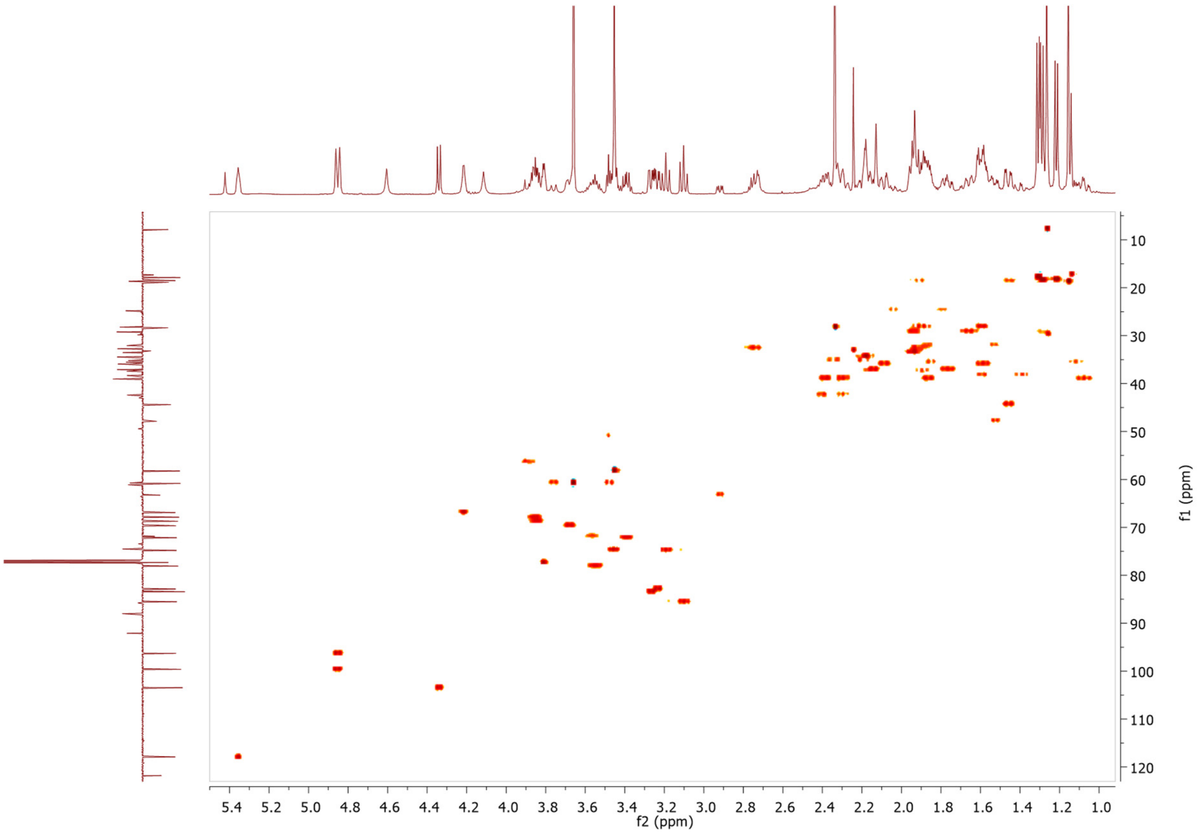

Figure S44. HSQC spectrum of compound **8** (in $CDCl_3$).

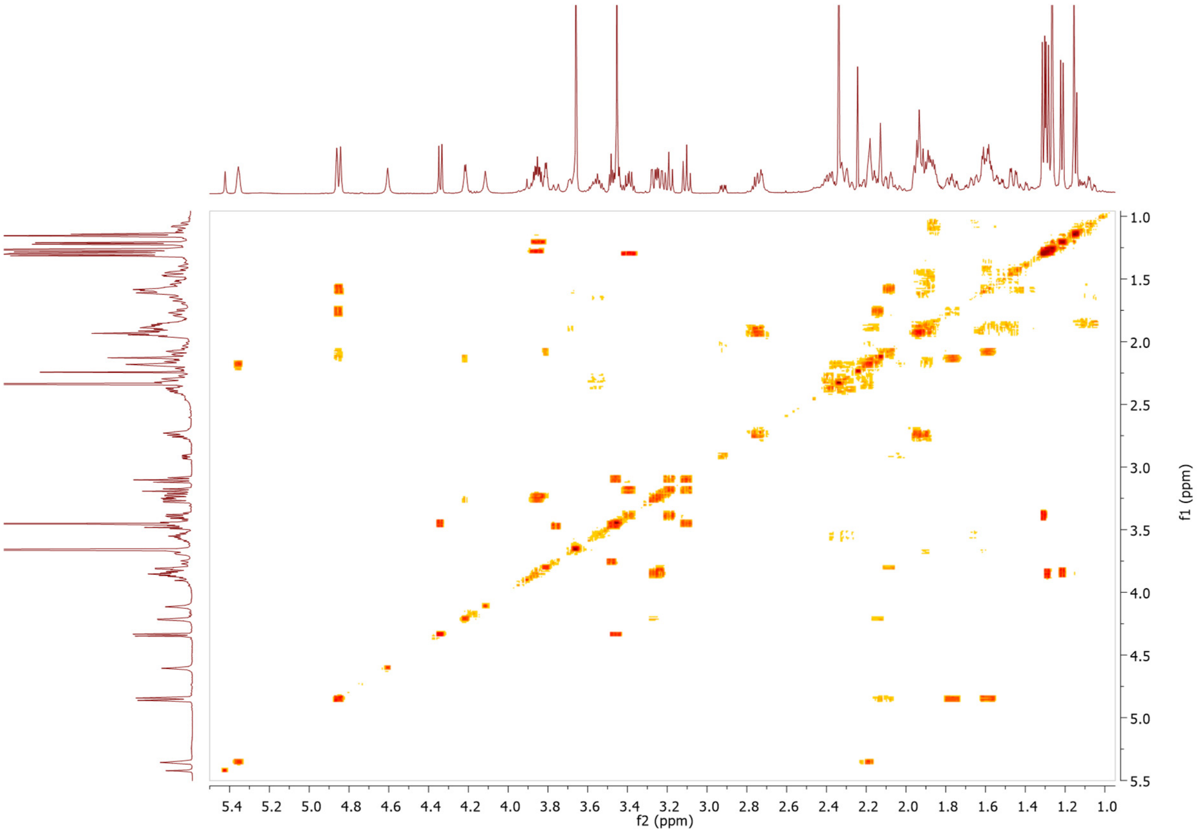

Figure S45. ^{1}H-^{1}H COSY spectrum of compound **8** (in $CDCl_3$).

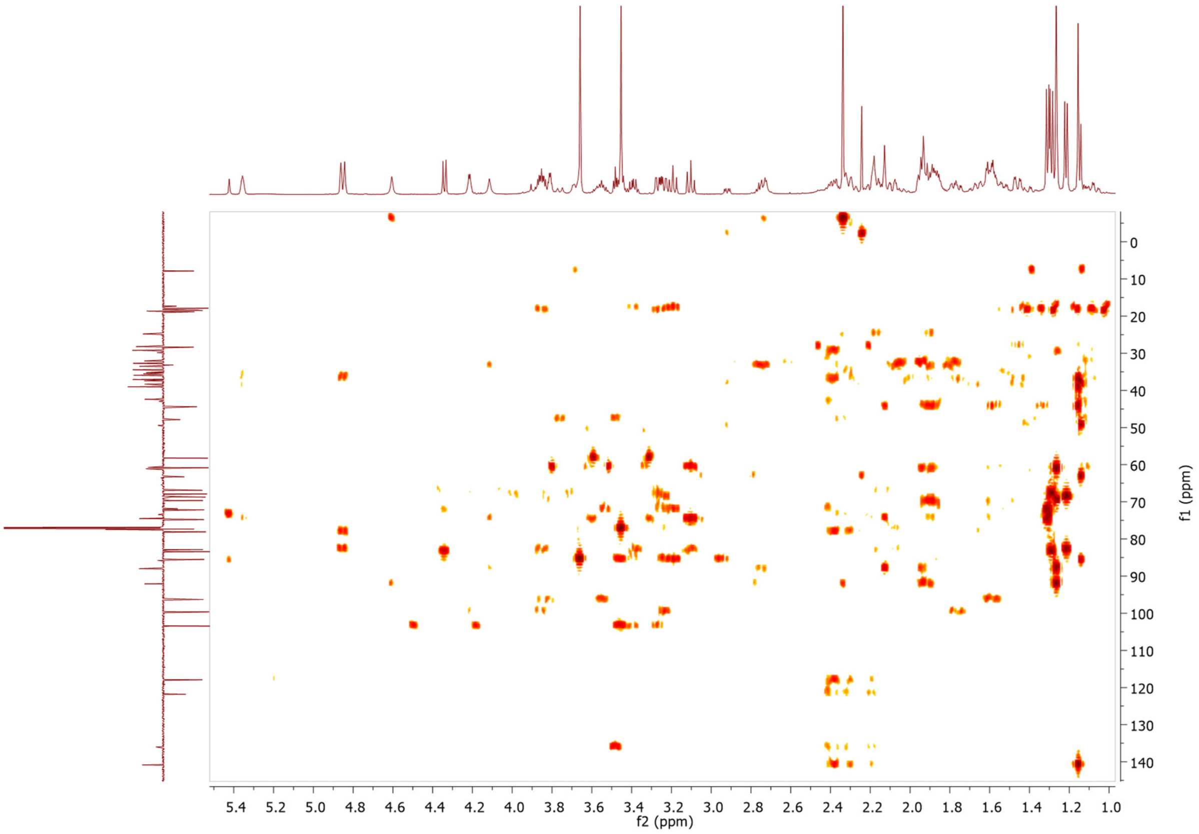

Figure S46. HMBC spectrum of compound **8** (in $CDCl_3$).

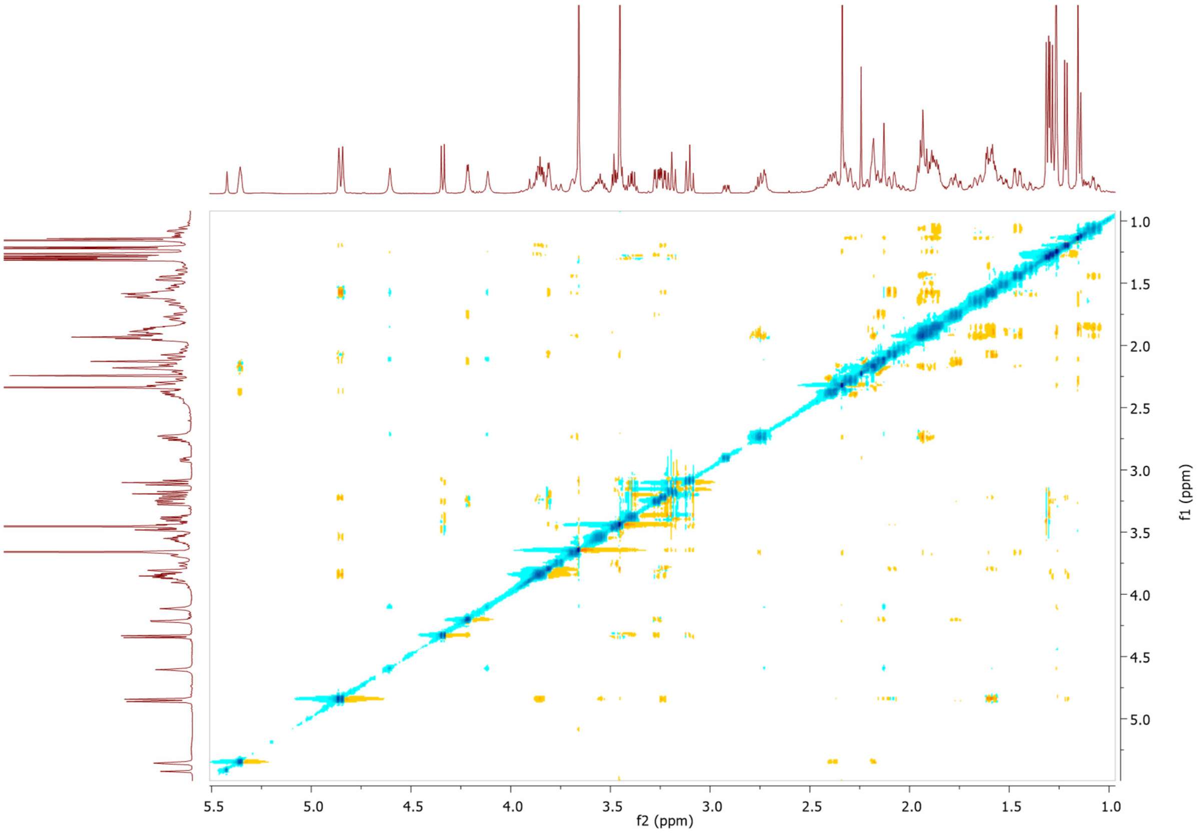

Figure S47. NOESY spectrum of compound **8** (in $CDCl_3$).

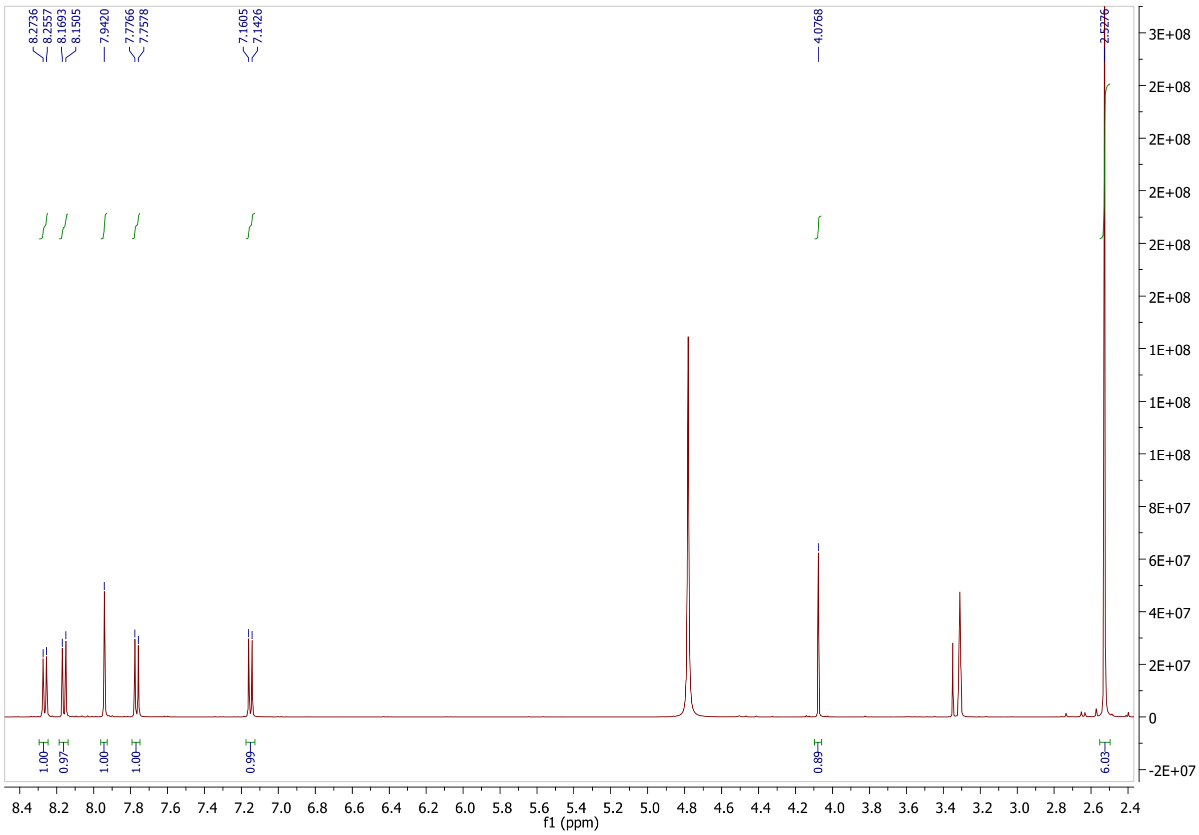

Figure S48. ^{1}H NMR spectrum of compound **9** (500 Mz, in CD_3OD).

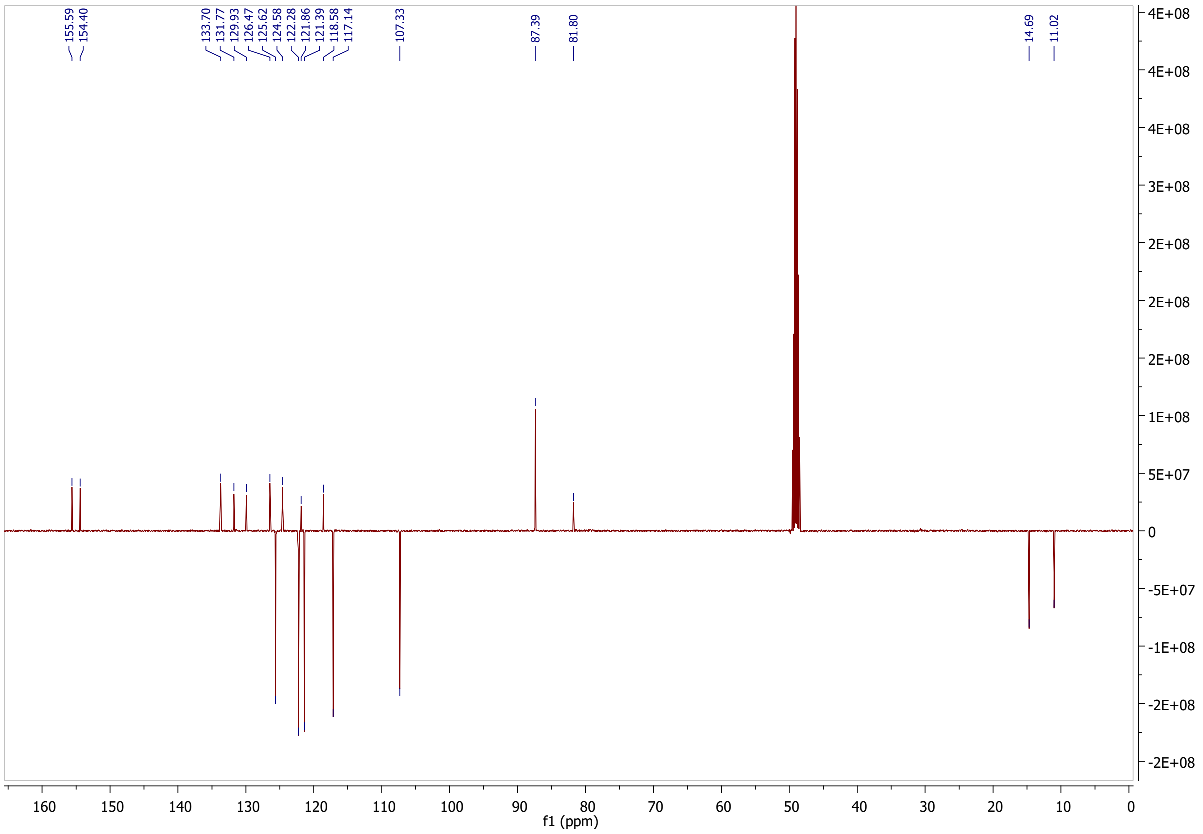

Figure S49. ^{13}C (JMOD) NMR spectrum of compound **9** (125 MHz, in CD_3OD).

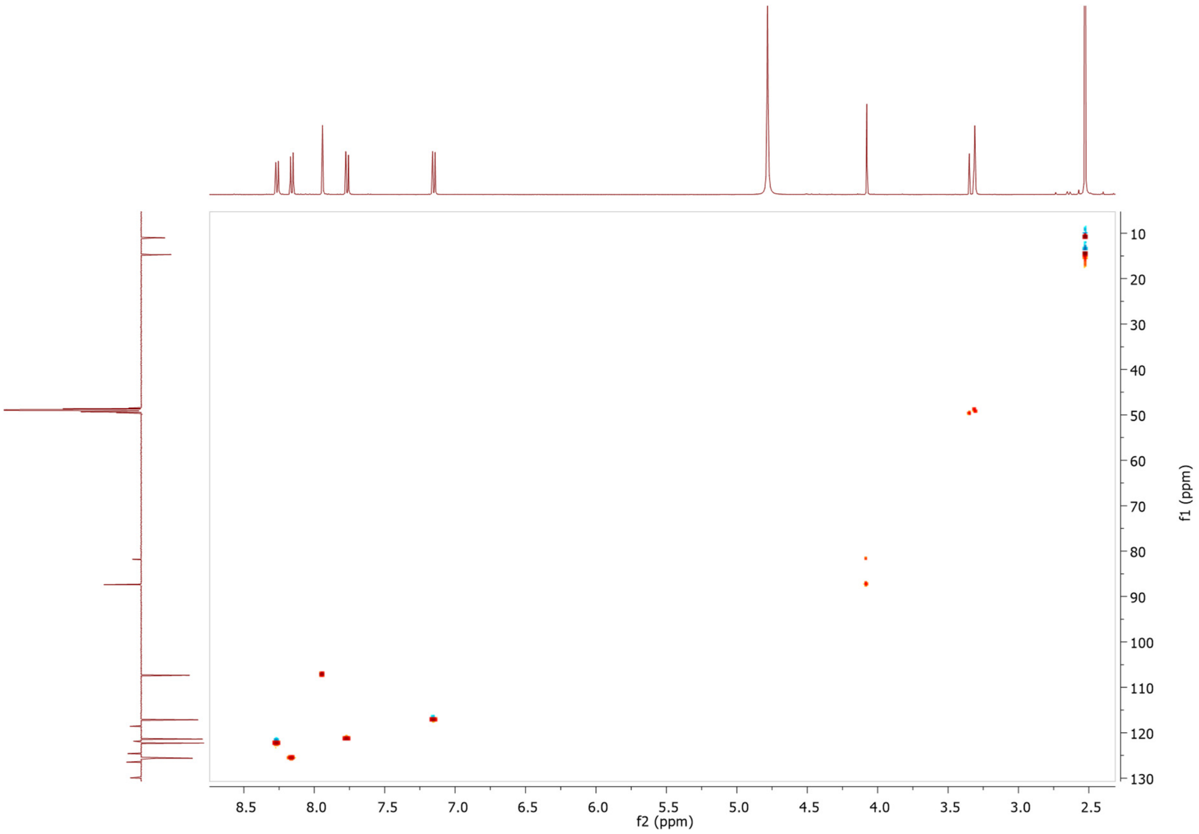

Figure S50. HSQC spectrum of compound **9** (in CD_3OD).

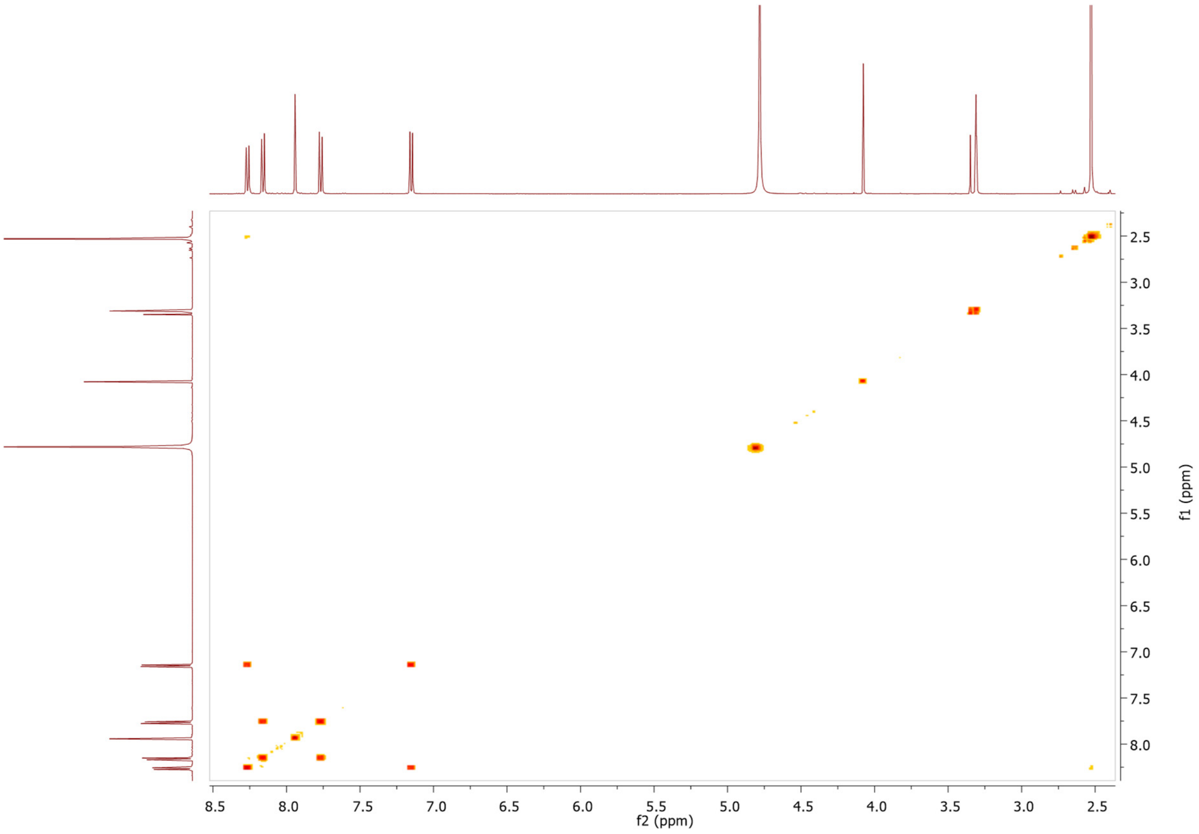

Figure S51. ^1H-^1H COSY spectrum of compound **9** (in CD_3OD).

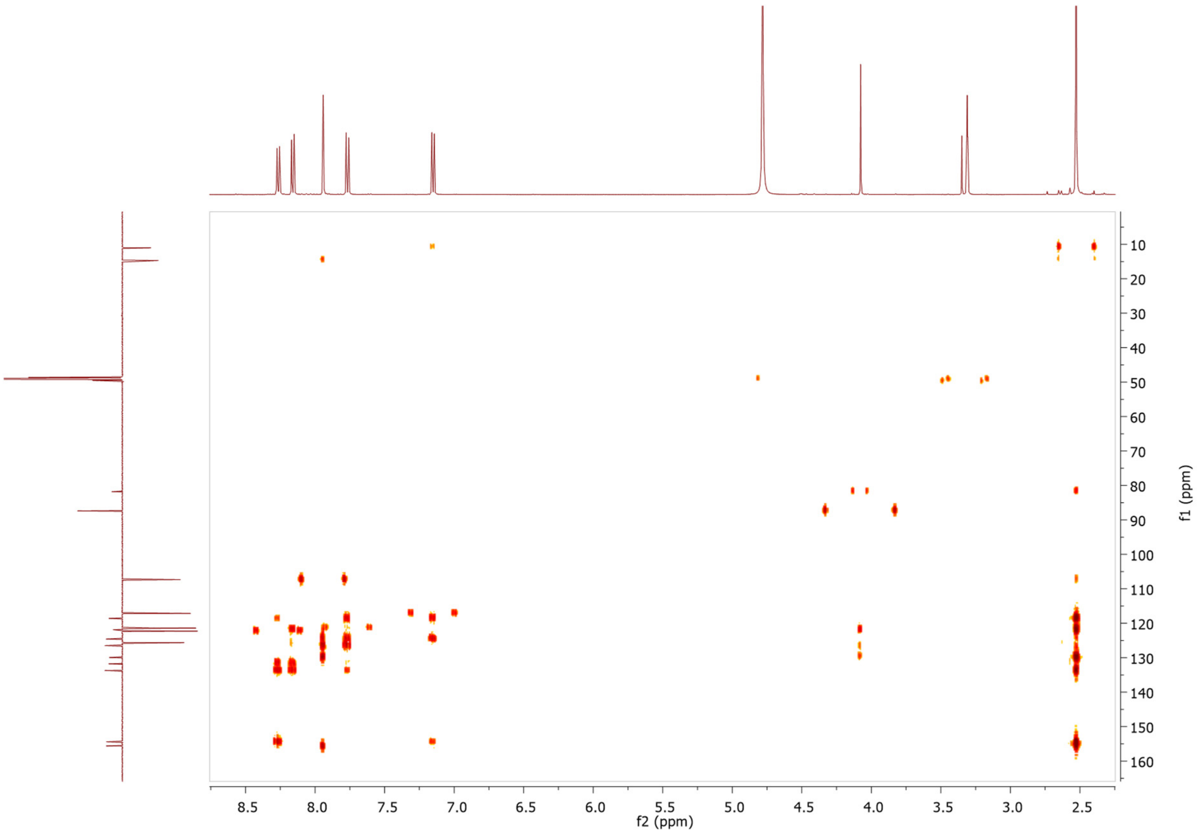

Figure S52. HMBC spectrum of compound **9** (in CD_3OD).

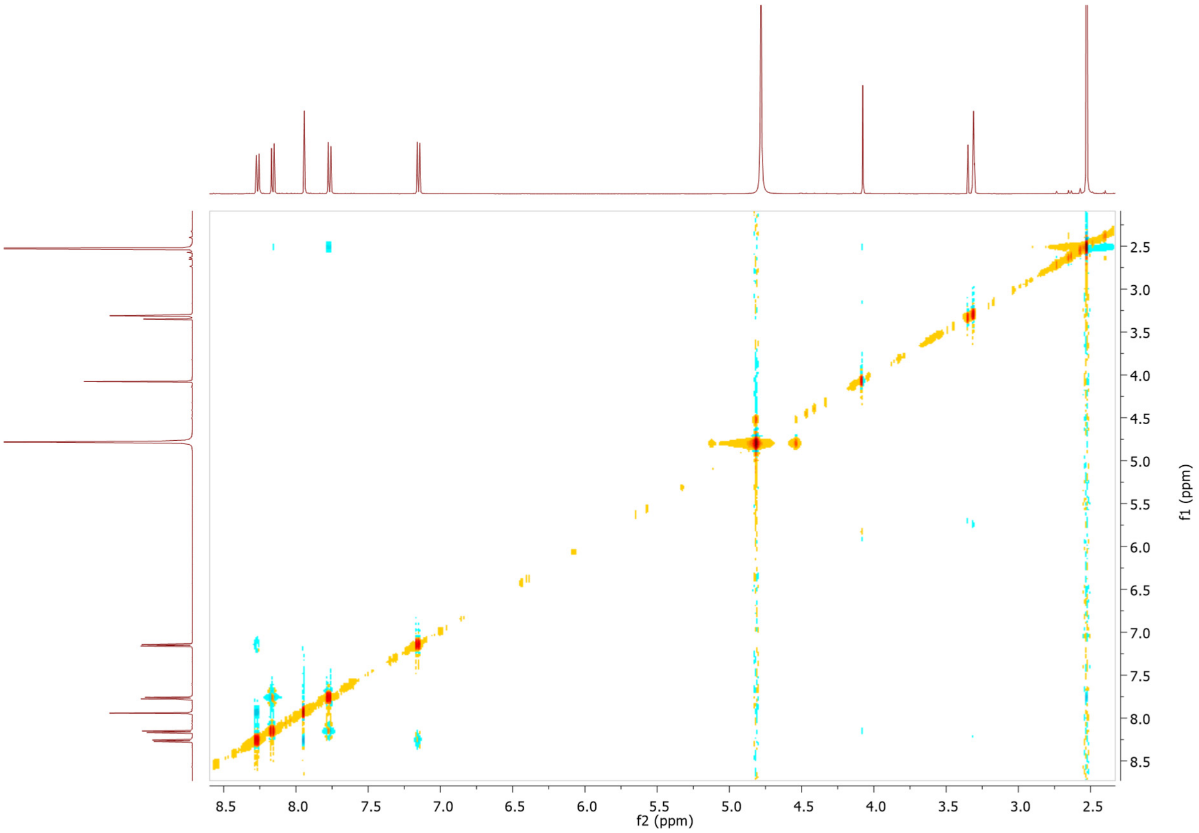

Figure S53. NOESY spectrum of compound **9** (in CD_3OD).

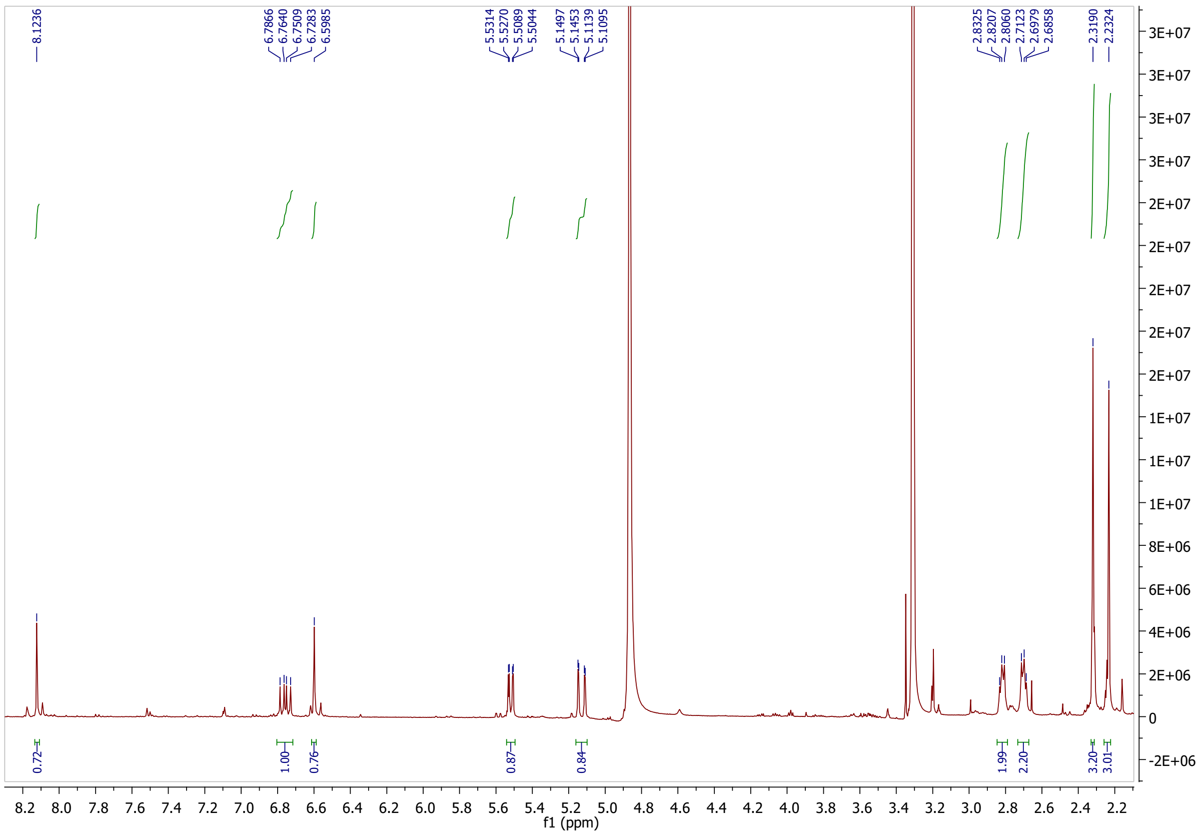

Figure S54. ^{1}H NMR spectrum of compound **10** (500 Mz, in CD_3OD).

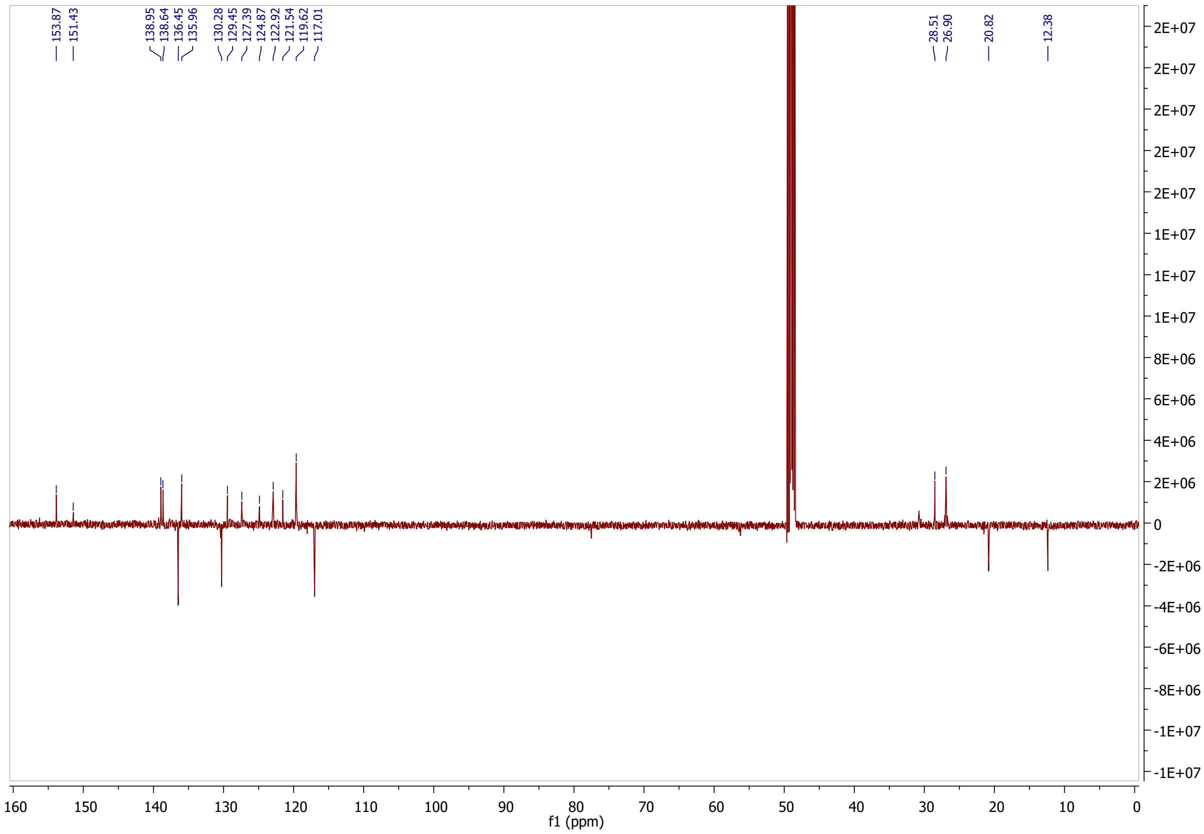

Figure S55. ^{13}C (JMOD) NMR spectrum of compound **10** (125 MHz, in CD_3OD).

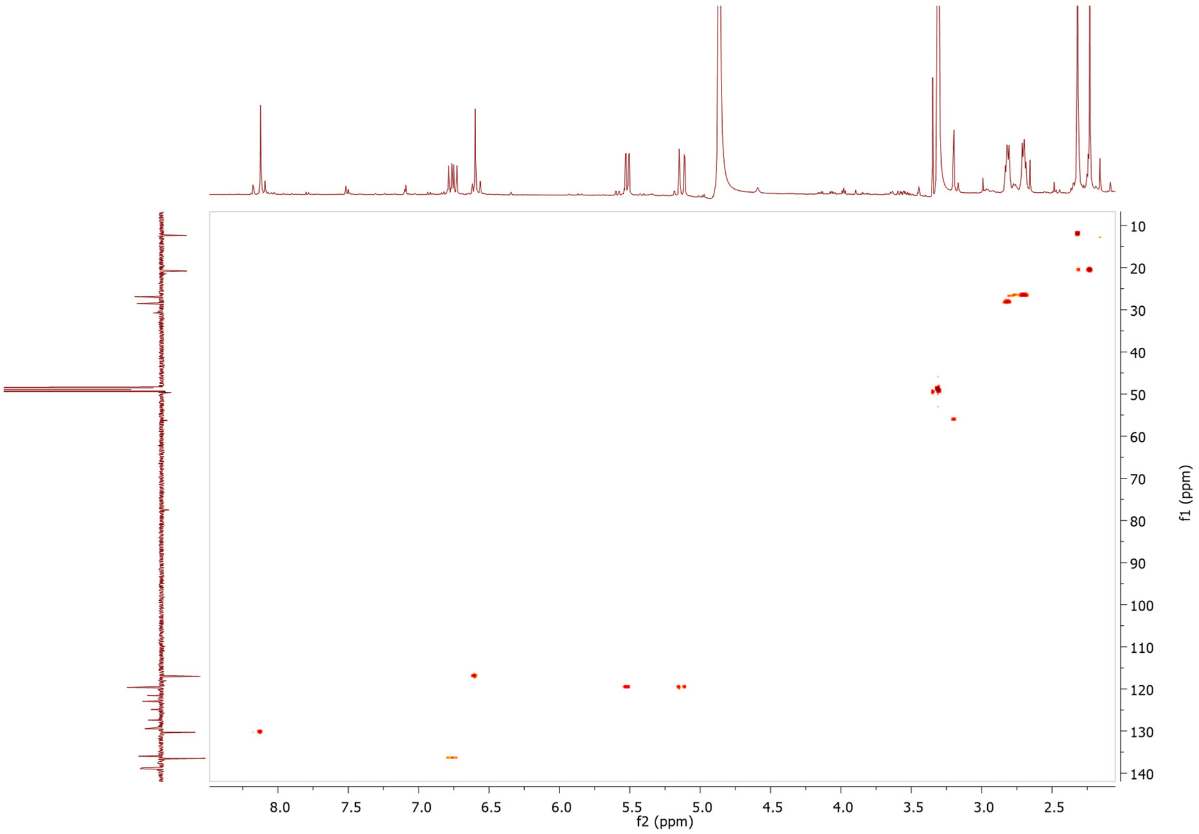

Figure S56. HSQC spectrum of compound **10** (in CD_3OD).

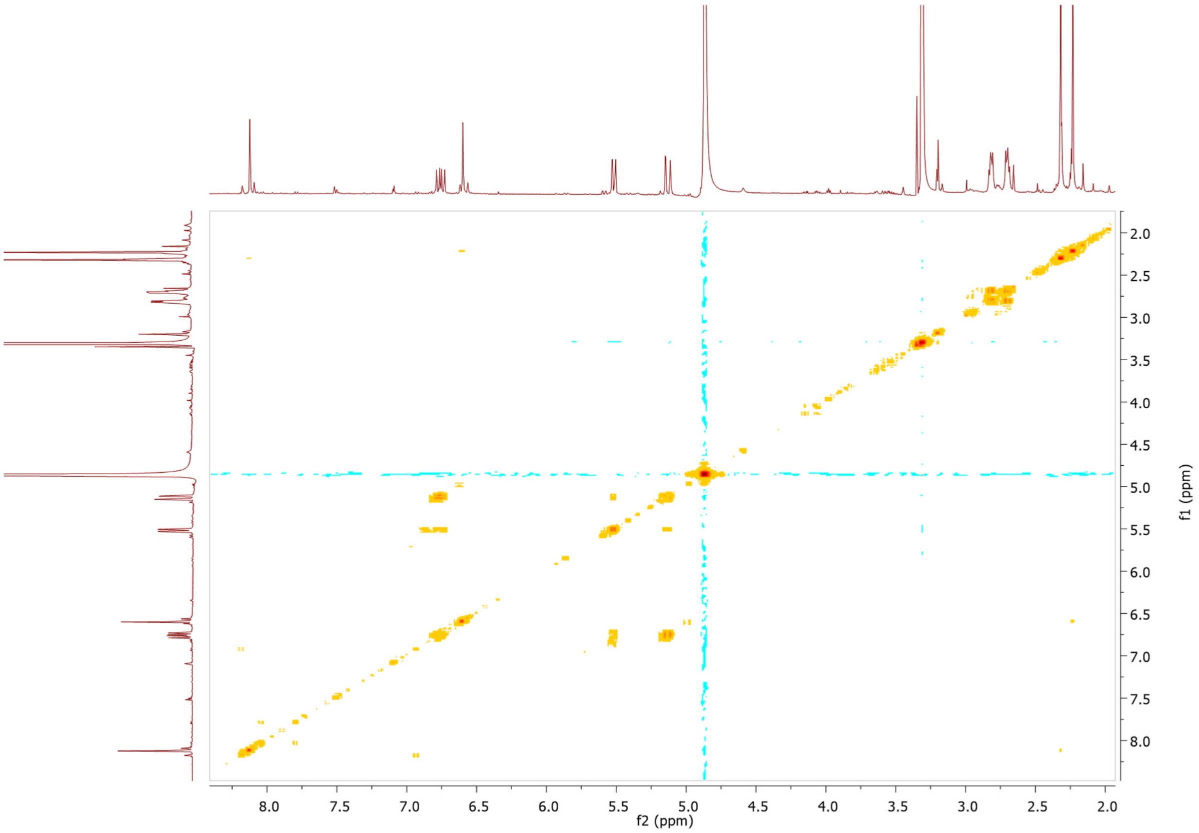

Figure S57. ^1H-^1H COSY spectrum of compound **10** (in CD_3OD).

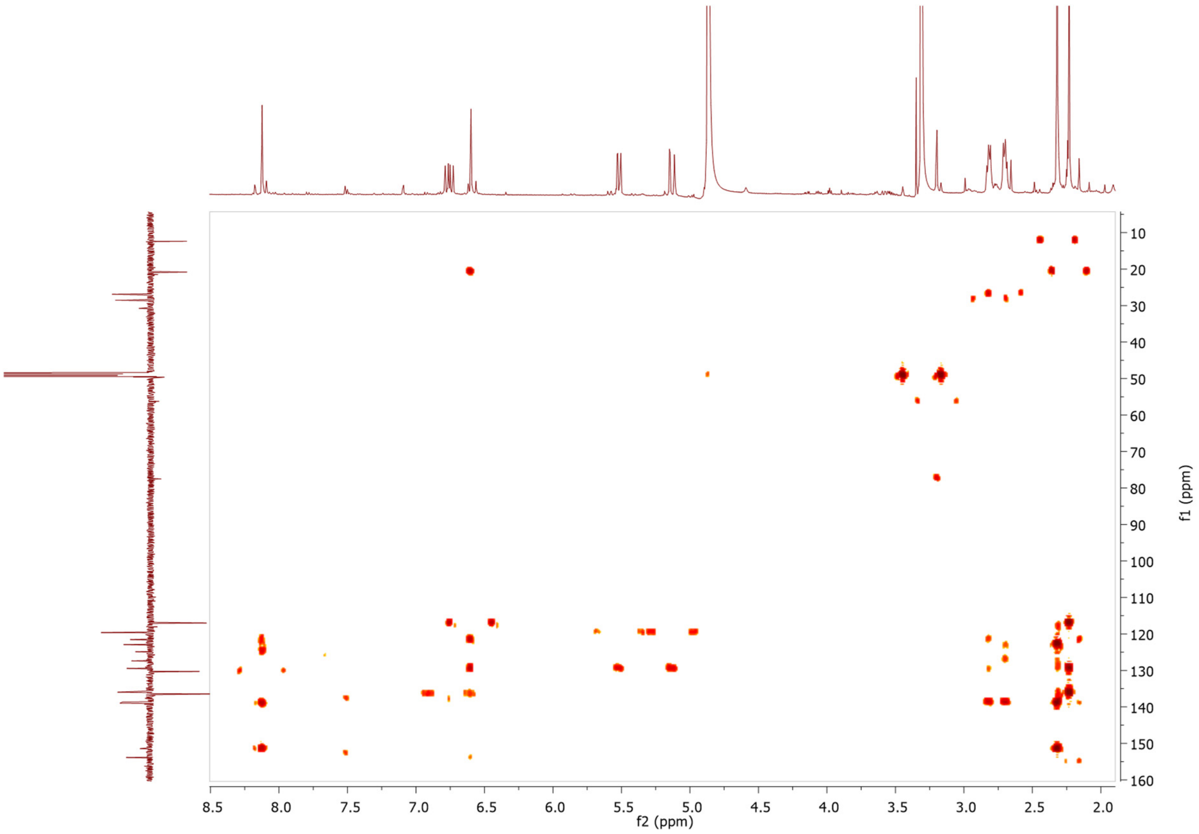

Figure S58. HMBC spectrum of compound **10** (in CD_3OD).

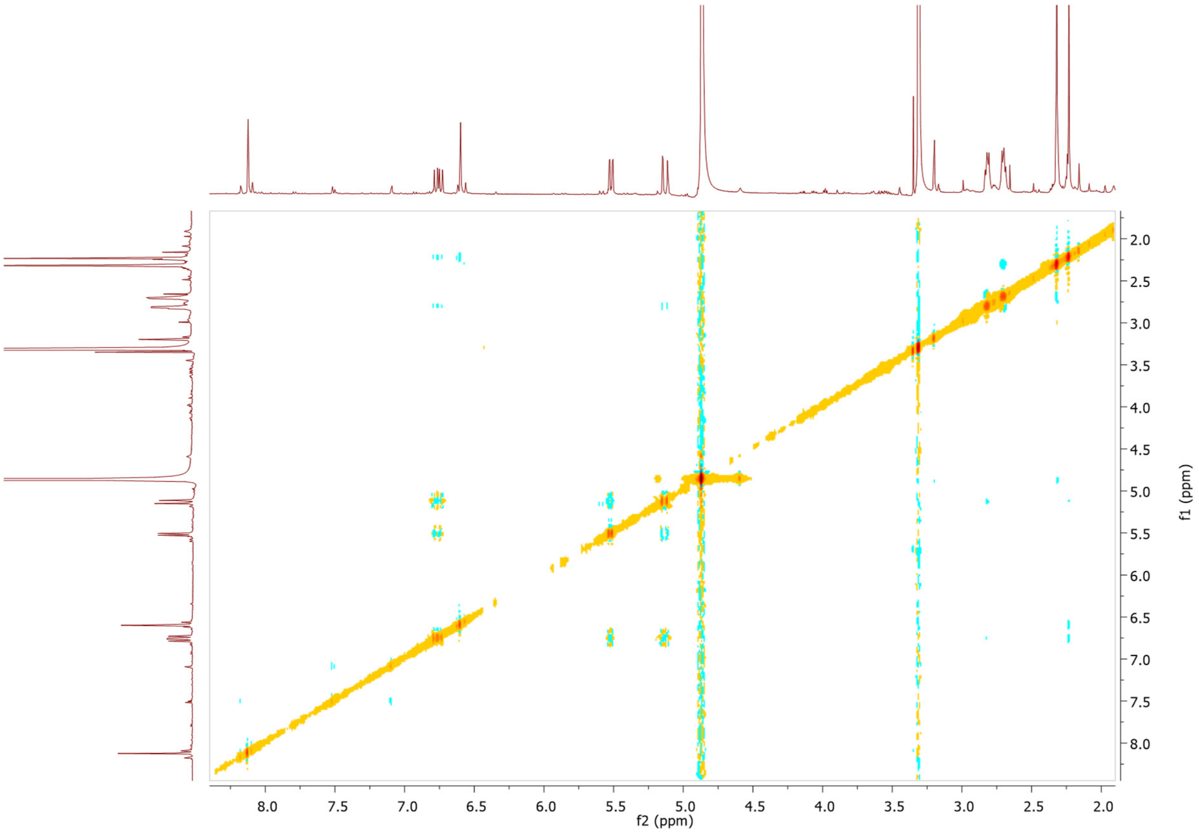

Figure S59. NOESY spectrum of compound **10** (in CD_3OD).

Table S1. Antiproliferative activity of the isolated compounds (**1**-**3**, **5**-**12**) in HeLa cell line.

Compounds	IC_{50} (µM)	SD (+/-)
**1**	52,38	0,23
**2**	>100	-
**3**	>100	-
**5**	>100	-
**6**	>100	-
**7**	>100	-
**8**	>100	-
**9**	>100	-
**10**	>100	-
**11**	>100	-
**12**	>100	-
**DOX**	0,02	0,003
**CIS**	2,07	0,07
**DMSO**	>2 V/V%	-
